# Facile Synthesis of *N*-vinylindoles via Knoevenagel Condensation: Molecular Features and Biological Activities

**DOI:** 10.3390/ijms262010149

**Published:** 2025-10-18

**Authors:** Anita Kornicka, Justyna Stefanowicz-Hajduk, Katarzyna Turecka, Christophe Furman, Maria Gdaniec, Łukasz Balewski

**Affiliations:** 1 Department of Chemical Technology of Drugs, Faculty of Pharmacy, Medical University of Gdansk, Gen. J. Hallera 107, 80-416 Gdansk, Poland; lukasz.balewski@gumed.edu.pl; 2 Department of Biology and Pharmaceutical Botany, Faculty of Pharmacy, Medical University of Gdansk, Gen. J. Hallera 107, 80-416 Gdansk, Poland; justyna.stefanowicz-hajduk@gumed.edu.pl; 3 Department of Pharmaceutical Microbiology, Faculty of Pharmacy, Medical University of Gdansk, Gen. J. Hallera 107, 80-416 Gdansk, Poland; katarzyna.turecka@gumed.edu.pl; 4 University of Lille, Inserm, CHU Lille, Institut Pasteur de Lille, UMR 1167—RID-AGE—Risk Factors and Molecular Determinants of Aging-Related Diseases, F-59000 Lille, France; christophe.furman@univ-lille2.fr; 5 Faculty of Chemistry, Adam Mickiewicz University, 61-614 Poznan, Poland; maria.gdaniec@amu.edu.pl

**Keywords:** *N*-vinylindoles, Knoevenagel condensation, structure, antiproliferative properties, antimicrobial activity, RAGE inhibition

## Abstract

*N*-vinylindoles have attracted attention for their promising role in medicinal chemistry. Therefore, developing new synthetic methods that enable access to diverse functionalized *N*-vinylindoles with potential pharmacological properties is highly valuable. 1-[2-aryl-1-(4,5-dihydro-1*H*-imidazol-2-yl)vinyl]-1*H*-indoles **2a**-**i** were prepared via Knoevenagel condensation promoted by 1*H*-benzotriazole, and characterized by IR, NMR, and MS spectroscopic data as well as a single-crystal X-ray diffraction-based study of the representative derivative **2g**. The obtained compounds **2a**-**i** were screened for their cytotoxic potency against human cancer cell lines (HeLa, SKOV-3, AGS) and non-cancerous cell line (HaCaT) using the MTT assay. Additional apoptosis analysis and cell cycle assay on SKOV-3 cells were conducted. Their antimicrobial activity was determined using reference strains of *S. aureus*, *E. coli*, *C. albicans*, and *C. glabrata*. The potent inhibitory activity against AGE2-BSA/sRAGE interaction of selected *N*-vinylindoles **2b**, **2d**-**f**, and **2h**-**i** was evaluated by ELISA assay. A facile approach has been developed for the synthesis of a novel class of *N*-vinylindoles. The preliminary structure–activity considerations indicated that the presence of substituents R, such as 4-bromophenyl (compound **2f**) or 2-naphthyl (compound **2i**) is optimal for anticancer activity and the AGE2-BSA/sRAGE interaction inhibition. The most prominent (*Z*)-1-[1-(4,5-dihydro-1*H*-imidazol-2-yl)-2-(naphthalen-2-yl)vinyl]-1*H*-indole (**2i**) was found to strongly arrest cell cycle in the SKOV-3 cell line in the subG0 phase, inducing apoptosis. Notably, derivative **2i** also exhibited the highest activity against *S. aureus* and *C. albicans* strains within the tested series. These findings highlight the substantial potential of *N*-vinylindole derivative **2i** as a lead compound for the development of anticancer drugs with additional inhibitory activity on the AGE/RAGE interaction.

## 1. Introduction

Indole serves as the core structure for numerous biologically active compounds that display a broad range of pharmacological effects, such as antibacterial [1,2], antiviral [3], anti-neurodegenerative [4], analgesic and anti-inflammatory [5,6] or antidepressant [7], as well as anticonvulsant [8] and antidiabetic [9] activities, etc. Examples include sumatriptan, a serotonin 5-HT_1B_/5-HT_1D_ receptor agonist used as an antimigraine drug [10], or tryptophan, an essential amino acid that serves as a precursor to serotonin and is a neurotransmitter crucial for regulating mood and sleep [11]. Due to its bioavailability, distinctive chemical properties, and notable pharmacological potential, the indole scaffold is regarded as one of the most promising frameworks for anticancer drug development [12]. It plays a key role in the design of compounds acting as apoptotic inducers [13], aromatase inhibitors [14], estrogen receptor modulators [15], tyrosine kinase inhibitors [16], and tubulin polymerization disruptors [17] or regulators of the NF-κB/PI3K/Akt/mTOR signaling pathway [18], as well as HDAC inhibitors [19].

**Figure 1:**
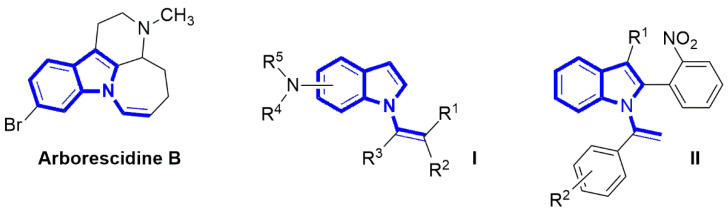
*N*-vinylindole moiety exemplified by the natural product Arborescidine B and synthetic *N*-vinylindoles **I**-**II**.

Among the indole derivatives, *N*-vinylindoles have attracted attention for their promising applications in medicinal chemistry and materials science. These compounds are commonly found as a core structure in various natural products, such as arborescidine B—an indole alkaloid with antiproliferative properties (Figure 1) [20]—and are used as intermediates in the synthesis of alkaloids [20,21], especially those of the vincane type, which are known for their anticancer and cardiovascular effects [22,23,24,25]. Additionally, *N*-vinylindoles of types **I** and **II** (Figure 1) have been found to possess anticancer activity. Their antiproliferative effect may be attributed to the inhibition of tubulin polymerization, which contributes to the suppression of cancer cell growth [26,27]. Finally, *N*-vinylindoles serve as monomers in the production of poly-*N*-vinylindole-based materials with semiconducting and photosensitive properties [28,29].

Various synthetic strategies have been developed for preparation of *N*-vinylindoles, including Pd-catalyzed stereocontrolled *N*-vinylation of indoles with alkenyl bromides [30], acid(TFA)-catalyzed one-step condensation of *NH*-indole derivatives and aldehydes [31], Ir-catalyzed tandem reaction of *o*-alkynylanilines with ketones [32], Cu-catalyzed cross-coupling reaction of *NH*-indole derivatives with alkenyl bromides [33], indirect *N*-vinylation of indoles via isomerization of their *N*-allyl derivatives [20], Pd-catalyzed oxidative cross-coupling between *NH*-indoles and *N*-tosylhyrazones [26,34,35], Cu-catalyzed intramolecular annulation of *N*-(2-alkynylphenyl)imine [36], base-mediated addition of *NH*-indoles to aromatic aldehydes in DMSO as a terminal carbon synthon [37], Ni-catalyzed [3 + 2]cycloaddition of unsaturated nitrones with allenoates [38], *N*-vinylation of *NH*-indole derivatives through addition/elimination of vinyl selenones in the presence of potassium hydroxide [39], and Cu-catalyzed cross-coupling of *NH*-indoles with vinyl halides [27] or propylphosphonic acid cyclic anhydride(T_3_P)-promoted condensation of 3-methylindole with ketones [40]. However, to the best of our knowledge, no method has been reported for the synthesis of *N*-vinylindoles bearing two heterocyclic units at the α-position of the vinyl group.

**Figure 2:**
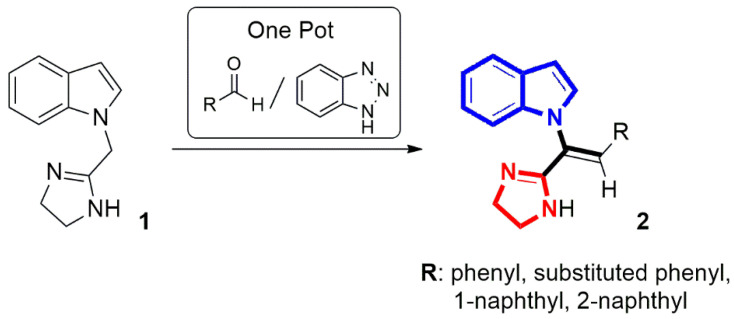
General structure of 1-[2-aryl-1-(4,5-dihydro-1*H*-imidazol-2-yl)vinyl]-1*H*-indole derivative **2,** studied in this work.

In this work, we present a mild, one-step synthesis of 1-[2-aryl-1-(4,5-dihydro-1*H*-imidazol-2-yl)vinyl]-1*H*-indoles of type **2**, containing two heterocyclic systems at the α-position of the vinyl group—indole and 4,5-dihydro-1*H*-imidazole, via Knoevenagel condensation of 1-[(4,5-dihydro-1*H*-imidazol-2-yl)methyl]-1*H*-indole (**1**) [41] with various aryl aldehydes in the presence of 1*H*-benzotriazole (Figure 2). It is also noteworthy that 4,5-dihydro-1*H*-imidazole derivatives, similar to indole-containing compounds, exhibit a broad spectrum of biological activities, such as antihypertensive, neuroprotective, antidepressant, anti-inflammatory, analgesic, anticancer properties, etc. [42].

Despite being valuable intermediates in the synthesis of pharmacologically active compounds [20,21,22,23,24,25], *N*-vinylindoles have not been extensively studied for their biological activities. To date, only a few reports on their antitumour properties could be found in the literature [26,27]. Accordingly, we carried out a preliminary assessment of the anticancer and antimicrobial activities of the newly synthesized compounds, which represent a new class of *N*-vinylindoles, along with an assessment of their potential as inhibitors of the RAGE–ligand interaction. RAGE (receptor for advanced glycation end-products) is a transmembrane receptor belonging to the immunoglobulin superfamily that can interact with a variety of both endogenous and exogenous ligands [43]. While RAGE and its ligands play a role in maintaining physiological homeostasis, they are also implicated in the promotion of inflammation and the progression of various diseases, including diabetes mellitus, atherosclerosis, neurodegenerative disorders, obesity, and cancer [43,44,45,46,47,48,49]. Consequently, blocking the RAGE–ligand interaction has emerged as a promising therapeutic approach for treating chronic inflammatory disorders and various types of cancer [49].

## 2. Results and Discussion

### 2.1. Chemistry

**Scheme 1:**
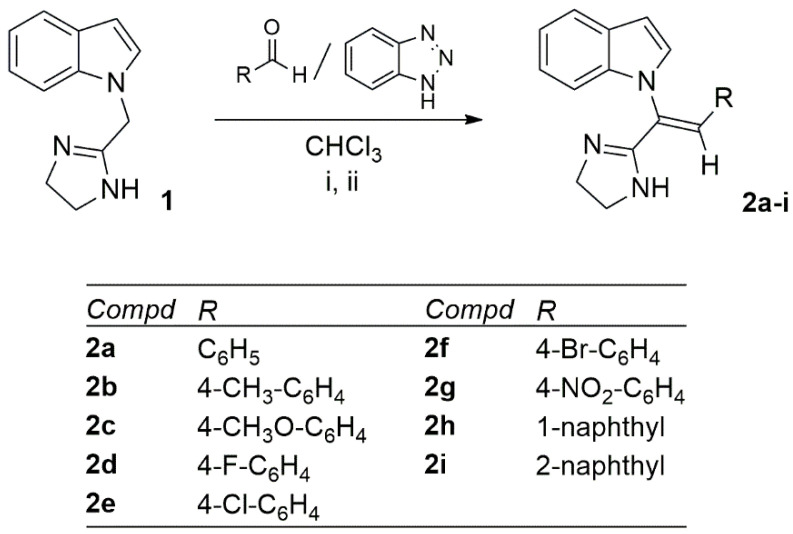
Route for the synthesis of *N*-vinylindoles **2a**-**i**. Reaction conditions: (i) 1-[(4,5-dihydro-1*H*-imidazol-2-yl)methyl]-1*H*-indole (1, 1.5 mmol), 1*H*-benzotriazole (1.8 mmol), CHCl_3_ (5 mL), r.t. for 30 min; and (ii) aromatic aldehydes (1.8 mmol), r.t. for 30 min, reflux for 6–12 h.

The pivotal intermediate 1-[(4,5-dihydro-1*H*-imidazol-2-yl)methyl]-1*H*-indole (**1**) for the synthesis of *N*-vinylindole analogs **2a**-**i** was prepared from 1*H*-indole and 2-chloromethylimidazoline according to the previously described procedure [41]. Then, the *N*-vinylindoles **2a**-**i** were synthesized via 1*H*-benzotriazole-promoted Knoevenagel condensation of compound **1** with suitable aromatic aldehydes (Scheme 1). The synthesis was performed in chloroform under reflux for a varying period, yielding the final products **2a**-**i** in yields ranging from 22% to 41%. Reaction progress was controlled by thin-layer chromatography (TLC).

**Scheme 2:**
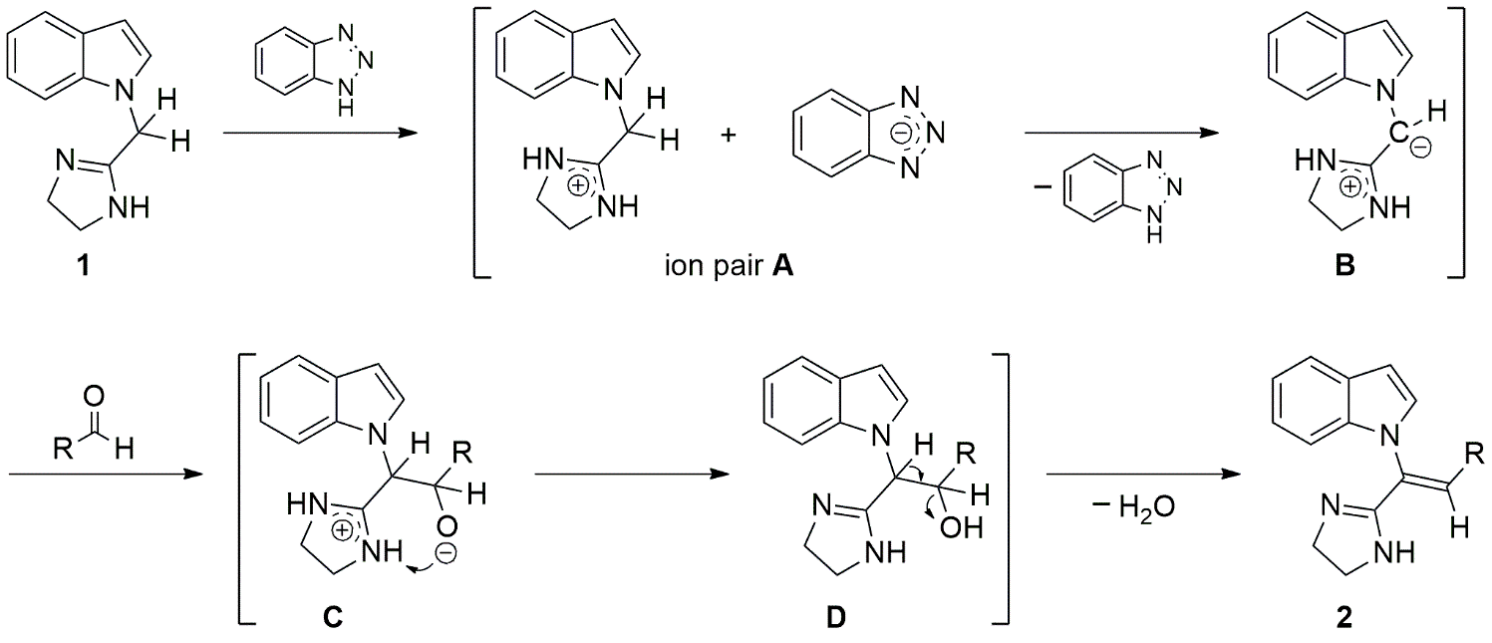
Plausible mechanism of Knoevenagel condensation cascade.

The proposed mechanism of the Knoevenagel condensation cascade leading to the formation of *N*-vinylindole derivatives **2a**-**i** is depicted in Scheme 2.

According to our previous work [50], we assumed that 1*H*-benzotriazole as an *N*H-acid (p*K_a_* = 8.2) protonates the imidazoline nitrogen atom of compound **1** which leads to the formation of ion pair **A**. In this route, a benzotriazole anion with strong nucleophilic properties is formed, whereas protonation of the imidazoline ring increases the electron deficit at the C-H methylene moiety of compound **1**, activating this position for a nucleophilic attack. Thus, the next step is the deprotonation of 1-[(4,5-dihydro-1*H*-imidazol-2-yl)methyl]-1*H*-indole cation in the presence of benzotriazole anion. The resulting intermediate zwitterion **B** upon reaction with the aldehyde forms derivative **C**. The process is completed by intramolecular hydrogen atom shift and the elimination of a water molecule in the intermediate **D** to form the final product **2**.

The structures of the newly synthesized *N*-vinylindoles **2a**-**i** were verified using elemental analysis, IR, 1D and 2D NMR spectroscopy, mass spectrometry, as well as X-ray crystallography studies, with the results detailed in the experimental section (Section 3.1. and Supplementary Materials, Figures S1–S41).

**Scheme 3:**
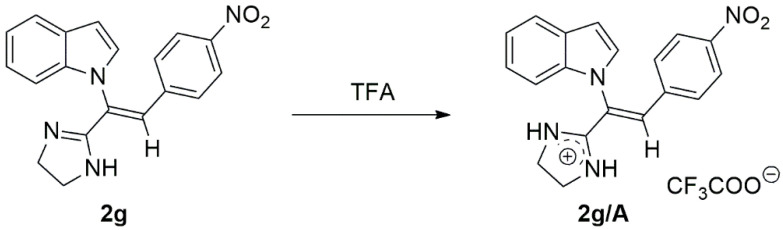
Protonation of compound **2g** upon treatment with TFA.

As an example, the ^1^H-NMR spectrum of compound **2g**, recorded in DMSO-*d_6_* at 400 MHz, exhibited a singlet at 3.59 ppm corresponding to the methylene protons of the 4,5-dihydro-1*H*-imidazole ring. The protons of the indole and substituted benzene rings were visible as distinct multiples at 6.66, 6.94–6.97, 7.00–7.06, 7.23, 7.61–7.62, and 7.98 ppm. A broad signal at 6.82 ppm was attributed to the NH proton of the imidazoline ring, while the vinyl proton resonated as a singlet at 7.60 ppm (Supplementary Materials, Figure S26). In turn, the ^13^C-NMR spectrum of compound **2g**, recorded in DMSO-*d_6_* at 100 MHz, revealed 14 signals in the range of 104.1–147.2 ppm, which were assigned to eight indole, six *p*-nitrophenyl and two vinyl carbon atoms. Additionally, one signal at 163.1 ppm can be attributed to the C2′ carbon atom of the 4,5-dihydro-1*H*-imidazole ring. On the other hand, the signals corresponding to the aliphatic carbon atoms of the 4,5-dihydro-1*H*-imidazole moiety were not observed (Supplementary Materials, Figure S27). Therefore, the ^1^H- and ^13^C-NMR spectra of *N*-vinylindole **2g** were recorded in DMSO-*d_6_* with addition of TFA. The protonation of the 4,5-dihydro-1*H*-imidazole moiety of **2g** upon treatment with TFA, as shown in Scheme 3, allowed for clarification of the NMR.

Some new features were observed in the NMR spectra of the protonated compound **2g**/**A**. Thus, in the ^1^H-NMR spectrum recorded at 500 MHz, a singlet corresponding to the NH protons of the imidazoline ring appeared at 10.43 ppm, while the vinyl proton gave rise to a singlet at 8.04 ppm (Supplementary Materials, Figure S28). The ^13^C-NMR spectrum of **2g**/**A**, recorded at 125 MHz, revealed a sharp signal at 45.3 ppm attributable to the C4′ and C5′ carbon atoms of the imidazoline ring (Supplementary Materials, Figure S29). For the structural analysis of the protonated form **2g**/**A**, heteronuclear single quantum coherence (HSQC, Supplementary Materials, Figure S30) and heteronuclear multiple bond correlation (HMBC, Supplementary Materials, Figure S31) spectra were also employed.

**Figure 3:**
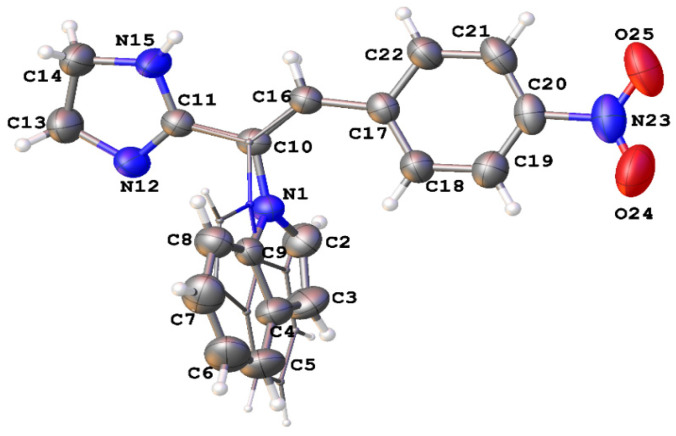
Molecular structure of **2g**. Displacement ellipsoids are shown at the 50% probability level. Atoms of the indole group in the minor position are shown as small spheres.

**Figure 4:**
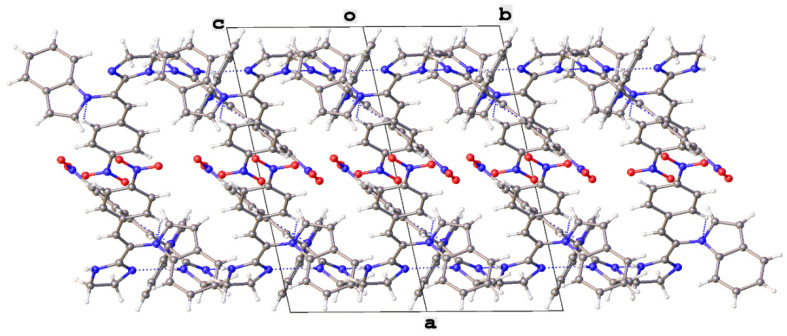
Crystal packing of **2g** viewed along the [011] direction. Hydrogen bonds and short contacts are shown with dotted lines.

Finally, the structure of *N*-vinylindole **2g** was unambiguously confirmed through X-ray crystallographic analysis (Figure 3 and Figure 4, see Supplementary Materials, Figure S41).

Compound **2g** crystallizes in the *P2_1_*/*c* monoclinic space group with one molecule in the asymmetric unit. The molecule of **2g** adopts a Z configuration at the C10-C16 double bond. The central part of the molecule consisting of C10, C16, C11, N1, and C17 atoms is virtually planar. The planes of imidazolidine and the nitrophenyl groups are only slightly twisted relative to the best plane through these atoms, forming dihedral angles of 7.55 and 12.15°, respectively. In turn, the indole group is oriented approximately perpendicular to this central plane with the dihedral angle between the planes of 78.96°. Interestingly, the indole group in the crystal exhibits disorder. It adopts two different nearly coplanar orientations resulting from the rotation by ca. 180° around the C10-N1 bond (Figure 3).

The crystal packing is mostly driven by intermolecular N-H⋯N hydrogen bond interaction (N15-H15⋯N12^i^: N15⋯N12^i^ 2.970(2) Å, H15⋯N12^i^ 2.12(2) Å, <N15-H15⋯N12^i^ 172(1)°, symmetry code i: x, -y + 1/2, z + 1/2) between the imidazoline fragments that assembles molecules into chains extending along the [001] direction. The π-π stacking interactions between the nitrophenyl groups (centroid–centroid distance 3.813 Å, interplanar distance 3.509 Å) and C-H⋯O interactions between the indole and nitro groups (C2-H2⋯O25^i^: C2⋯O25^i^ 3.359(2) Å, H2⋯O25^i^ 2.57 Å, < C2-H2⋯O25^i^ 143°, symmetry code i:

-x + 1, -y + 1, -z + 1; C7A-H7A⋯O24^ii^: C7A⋯O24^ii^ 3.387(2) Å, H7A⋯O24^ii^ 2.68 Å, < C7A-H7A⋯O24^ii^ 134°, symmetry code i: -x + 1, y-1/2, -z + 1/2) organize the chains into the (100) layers. There are no contacts shorter than the sum of van der Waals radii between the atoms from neighboring layers (Figure 4).

### 2.2. Biological Evaluation

#### 2.2.1. In Vitro Anticancer Activity

##### Cytotoxic Activity of *N*-vinylindole Derivatives on Cancer Cell Lines

The MTT assay was used to assess the cytotoxic effects of the newly synthesized *N*-vinylindoles **2a**-**i** on cancer cell lines (HeLa, SKOV-3, and AGS) and non-cancerous keratinocytes (HaCaT). As reported in Table 1, the obtained IC_50_ values were in the range from 2.03 µg/mL to 18.90 µg/mL. The cervical HeLa and ovarian SKOV-3 cancer cell lines were more sensitive to all the tested compounds than the gastric cancer AGS cell line. In the case of the HeLa cell line, the strongest effect was found for compounds **2f** and **2i** with IC_50_ values of 2.37 ± 0.15 µg/mL and 2.03 ± 0.05 µg/mL, respectively, while **2h** exhibited the weakest activity (IC_50_ = 10.09 ± 1.12 µg/mL). Similar trends were observed for the SKOV-3 cell line, with **2f** and **2i** being the most active (IC_50_ = 2.76 ± 0.18 µg/mL and 2.45 ± 0.13 µg/mL, respectively), and **2h** the least (IC_50_ = 10.39 ± 0.10 µg/mL). The tested compounds **2a**-**i** were moderately active on gastric cancer AGS cell line with the lowest IC_50_ value of 5.68 ± 0.41 µg/mL for **2i** and the highest IC_50_ value of 18.90 ± 1.81 µg/mL for **2h**.

Compared to anticancer drug *vinblastine sulfate*, which contains an indole scaffold, the investigated *N*-vinylindoles **2a**-**i** showed significantly lower potency against HeLa and SKOV-3 cell lines. However, these compounds were several times more potent than the broad-spectrum anticancer chemotherapeutic *oxaliplatin* [51] in the same cell lines (IC_50_ = 2.03–10.39 µg/mL vs. IC_50_ = 35.76–102.16 µg/mL) (Table 1).

The results obtained for compounds **2a**-**i** showed that the most active compound **2i** on all tested cancer cell lines possesses a 2-naphthyl group as the R substituent (IC_50_ = 2.03–5.68 µg/mL). Interestingly, replacement of the 2-naphthyl group in compound **2i** with 1-naphthyl substituent resulted in the least potent compound, **2h,** in this series, although it still displayed moderate activity (IC_50_ = 10.09–18.90 µg/mL). Moreover, the 4-bromophenyl substituted compound **2f** (R = 4-Br-C_6_H_4_) proved to be slightly less potent than derivative **2i** toward HeLa and SKOV-3 cell lines (IC_50_ = 2.37–2.76 µg/mL vs. IC_50_ = 2.03–2.45 µg/mL). Introduction of substituents R such as 4-chlorophenyl (compound **2e**, R = 4-Cl-C_6_H_4_) or 4-nitrophenyl groups (compound **2g**, R = 4-NO_2_-C_6_H_4_) resulted in a slight further reduction in activity against HeLa and SKOV-3 cell lines (IC_50_ = 3.13–4.40 µg/mL and 5.60–7.06 µg/mL, respectively). On the other hand, the presence of substituents R such as phenyl (**2a**, R = C_6_H_4_), *p*-tolyl (**2b**, R = 4-CH_3_-C_6_H_4_), 4-methoxyphenyl (**2c**, R = 4-CH_3_O-C_6_H_4_) or 4-fluorophenyl (**2d**, R = 4-F-C_6_H_4_) yielded less active compounds with a comparable level of ability to inhibit the growth of HeLa and SKOV-3 cells (IC_50_ values in the range of 7.86–8.96 µg/mL and 8.80–10.25 µg/mL, respectively). Nonetheless, the activity of the latter compounds remained favorable (Table 1).

**Figure 5:**
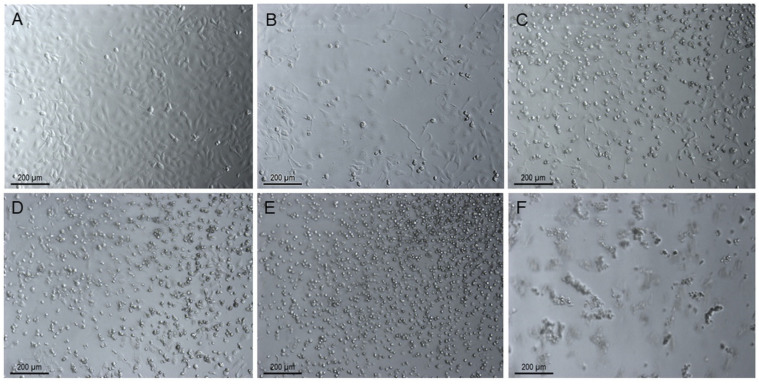
Microscopic observation of SKOV-3 cells treated with **2i** for 24 h: (**A**) ctrl (control cells with DMSO 0.3%, *v*/*v*) and (**B**–**F**) SKOV-3 cells treated with **2i** at concentrations of (**B**) 2, (**C**) 5, (**D**) 10, (**E**) 15, and (**F**) 30 µg/mL. Magnification × 100 (Leica DFC 310FX, Wetzlar, Germany).

Additionally, microscopic observation during the MTT assay confirmed the differential activity of the compounds **2a**-**i** on the tested cell lines. Notably, one of the most pronounced cytotoxic effects was observed for SKOV-3 cells and compound **2i,** which was documented by photography. Figure 5B–F shows the effect of derivative **2i** on the appearance of SKOV-3 cells compared to the non-treated control (Figure 5A). The morphological changes in these cells, as a result of treatment with compound **2i,** were clearly visible at higher concentrations of **2i**. The cells clearly shrank and detached from the bottom of the culture plate (Figure 5D–F).

Overall, the presented data suggest that either lipophilic or steric effects rather than electronic properties of the R substituents may contribute to the cytotoxic activity of this *N*-vinylindole series on cancer cells, as evidenced by comparing the activity of compounds **2c** or **2d** and **2f** as well as **2h** and **2i**. In particular, the introduction of a bulky lipophilic 2-naphthyl group led to compound **2i**, which showed optimal properties. On the other hand, the examined compounds **2a**-**i** generally exhibited modest selectivity toward HeLa and SKOV-3 cell lines compared to non-cancerous HaCaT cells, while their selectivity for AGS cell line was even worse (Table 1).

Typically, compounds with potent anticancer activity demonstrate similar effects on non-cancerous cells in biological assays. It is worth emphasizing that in vitro results do not necessarily have to be identical to in vivo results. In this study, only one non-cancerous HaCaT cell line was used, but in future studies we plan to conduct tests with a few normal cell lines to further explore the potential selectivity of these compounds.

##### Estimation of the Apoptotic Effect of **2i** in SKOV-3 Cells

**Figure 6:**
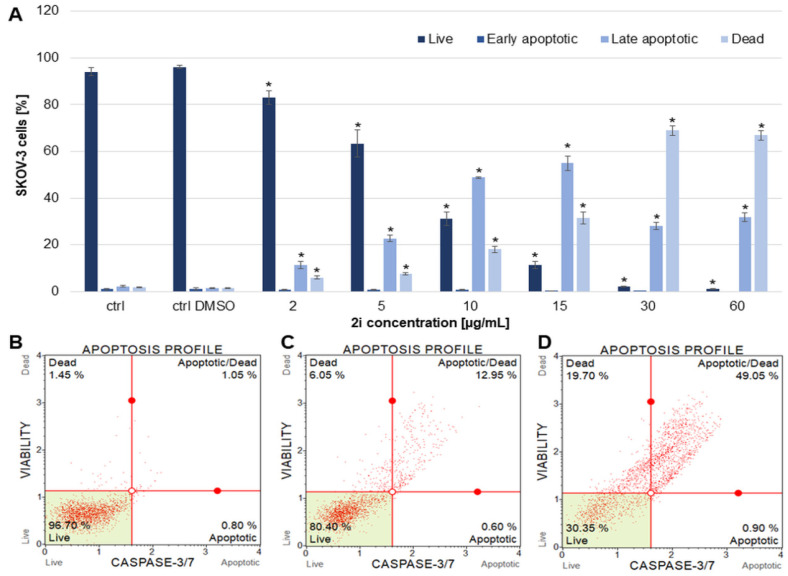
The apoptotic effect of **2i** in SKOV-3 cells after 24 h of treatment. The live, early apoptotic, late apoptotic, and dead cells are presented as percentage values (**A**) obtained from two independently repeated experiments (in three repetitions). The results were obtained by flow cytometry (Muse Cell Analyzer). (**B**) The cells treated with DMSO (0.3%, *v*/*v*); (**C**) the cells treated with the compound **2i** at a concentration of 2 µg/mL; and (**D**) the cells treated with the compound **2i** at a concentration of 10 µg/mL. Error bars indicate standard deviations. Significant differences relative to the control are marked with an asterisk “*” (*p* < 0.05).

Since compound **2i** proved to be the most effective at inhibiting the growth of cancer cells, its ability to trigger apoptosis in the representative SKOV-3 cell line was examined. The obtained results showed that compound **2i** significantly induced apoptosis from a concentration of 2 µg/mL to 60 µg/mL. At the lowest concentration used, the percentage of late apoptotic and dead cells was 11.52 ± 1.53% and 6.07 ± 0.58%, respectively. These populations increased and constituted 22.72 ± 1.37%, 48.85 ± 0.39%, 54.94 ± 3.12%, 28.21 ± 1.61%, and 31.87 ± 2.01% of late apoptotic cells for the compound concentrations of 5, 10, 15, 30, and 60 µg/mL, respectively. Dead cell populations were simultaneously 7.65 ± 0.61%, 18.11 ± 1.45%, 31.52 ± 2.68%, 68.95 ± 2.09%, and 66.85 ± 1.96% for the compound concentrations of 5, 10, 15, 30, and 60 µg/mL, respectively (Figure 6).

##### Cell Cycle Arrest Analysis of SKOV-3 Cells Treated with **2i**

**Figure 7:**
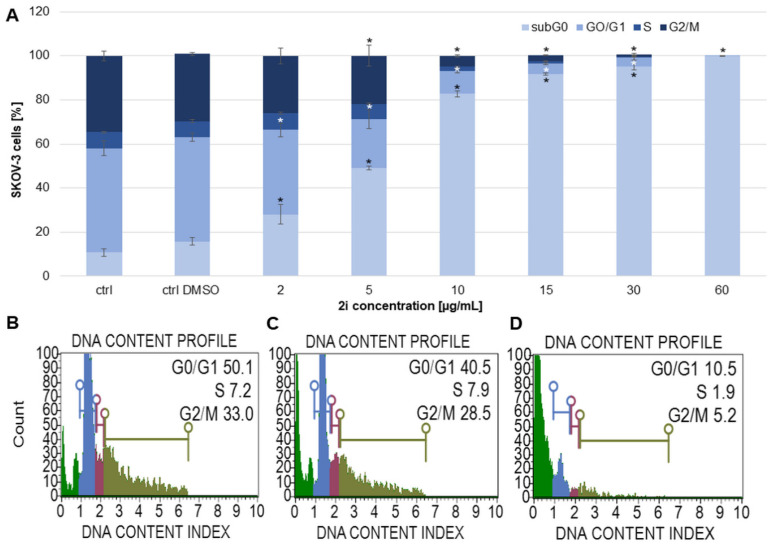
(**A**) Cell cycle distribution in a population of SKOV-3 cells treated with **2i** for 48 h. (**B**) The cells incubated with DMSO (0.3%, *v*/*v*); (**C**) the cells treated with the compound **2i** at a concentration of 2 µg/mL; and (**D**) the cells treated with the compound **2i** at a concentration of 10 µg/mL. The stained cells were analyzed by flow cytometry. The experiment was performed in two independently performed repeats (in three repetitions). Error bars represent standard deviations. Significant differences relative to the control are marked with an asterisk “*” (*p* < 0.05). Green—subG0, blue—G0/G1, red—S, khaki—G2/M.

To analyze cell cycle arrest of the SKOV-3 cells, flow cytometry was used after 48 h of treating the cells with the compound **2i** in a range of concentrations 2–60 µg/mL. The obtained results indicated that compound **2i** strongly induced cell cycle arrest in subG0 phase. This effect was observed at all used compound concentrations. The percentage of the cells in subG0 phase was 28.10 ± 4.49, 49.00 ± 0.85, 82.73 ± 1.33, 91.67 ± 0.57, 95.30 ± 1.67, and 99.83 ± 0.15% for the concentrations of 2, 5, 10, 15, 30, and 60 µg/mL, respectively. The amount of the cells in G0/G1, S, and M phases significantly decreased with increasing concentrations of the compound **2i** (Figure 7).

Consequently, the apoptotic effect of the newly synthesized *N*-vinylindole derivative **2i** on the SKOV-3 cells might result from cell cycle arrest in the subG0 phase. Nevertheless, a more detailed investigation is required to elucidate the molecular mechanism by which compound **2i** induces apoptosis.

#### 2.2.2. In Vitro Antimicrobial Activity

The antimicrobial properties of the synthesized *N*-vinylindoles **2a**-**i** were investigated on reference strains of Gram-positive bacteria—*Staphylococcus aureus* ATCC 6535 and methicillin-resistant *Staphylococcus aureus* (MRSA 12673)—and Gram-negative bacteria *Escherichia coli* ATCC 8739, as well as yeast species *Candida albicans* ATCC 10231 and *Candida glabrata* ATC 2001. The tests were conducted using the microbroth dilution method to determine the minimum inhibitory concentration (MIC), minimum bactericidal or fungicidal concentration (MBC/MFC). The obtained results are summarized in Table 2.

Among the tested microorganisms, *S. aureus* ATCC 6535 and *S. aureus* MRSA 12673 appeared to be the most susceptible to the synthesized compounds **2a**-**i**. It should be noted that *S. aureus* is a major bacterial human pathogen responsible for a broad range of clinical conditions. It is frequently associated with both community-acquired and hospital-acquired infections, and the treatment is often challenging due to the rise in multi-drug resistant strains, including methicillin-resistant *S. aureus* (MRSA) [52]. Notably, the 4-chlorophenyl derivative **2e** (R = 4-Cl-C_6_H_4_) and the 4-bromophenyl derivative **2f** (R = 4-Br-C_6_H_4_) exhibited relatively good bacteriostatic potency against these two strains, with MIC values ranging from 27.5 to 60 μg/mL. Nevertheless, their bactericidal effect was moderate or weak, as evidenced by MBC values between 55 μg/mL and 240 μg/mL. In turn, analogs **2b** and **2g** bearing *p*-tolyl and 4-nitrophenyl groups as the R substituents, respectively, showed moderate activity against *S. aureus* ATCC 6535 (MIC = 55 μg/mL, MBC = 110 μg/mL), whereas the activity of *N*-vinylindole derivatives with phenyl (compound **2a**, R = C_6_H_5_), 4-methoxyphenyl (compound **2c**, R = 4-CH_3_O-C_6_H_4_) or 4-fluorophenyl (compound **2d**, R = 4-F-C_6_H_4_) substituents R toward the two strains of *S. aureus* was low (MIC = 110–220 μg/mL, MBC = 220–240 μg/mL). Among all the compounds evaluated, the highest antibacterial activity was observed for compound **2i**, which features a 2-naphthyl substituent R. This derivative demonstrated pronounced potential against both *S. aureus* strains, ATCC 6535 and MRSA 12673, with MIC values of 15 μg/mL and 30 μg/mL, respectively, and MBC values of 30 μg/mL. In addition, *N*-vinylindole **2i** was characterized by relatively good antifungal activity against *C. glabrata* ATCC 2001, with MIC and MFC values of 60 μg/mL. Notably, its fungicidal effect was more potent than that of the standard drug *ketoconazole* (MFC = 60 μg/mL vs. 128 μg/mL). On the other hand, its isomer **2h** bearing a 1-naphtyl group as the R substituent showed mild antimicrobial activity (MIC = 120–>240 μg/mL, MBC/MFC ≥ 240 μg/mL).

It can be concluded that the structure–activity relationship (SAR) requirements for antimicrobial activity of this class of *N*-vinylindoles **2a**-**i** are not easily predictable, and there appears to be no direct correlation between anticancer and antimicrobial properties. However, compound **2i**, with the most potent antiproliferative activity toward cancer cells, also exhibited the highest antimicrobial properties. It is worth noting that cancer patients frequently suffer from immunodeficiency, which increases their risk of bacterial infections. In this context, developing a drug that combines anticancer and antibacterial effects could offer therapeutic advantages [53,54,55]. On the other hand, this observed relationship may arise from a non-specific mechanism of action, involving interactions with essential cellular structures such as the cytoplasmic membrane, enzymes, or nucleic acids. Such mechanisms are often shared by both prokaryotic and eukaryotic cells, which may explain the concurrent significant antibacterial activity and pronounced cytotoxicity toward mammalian cells. Therefore, the antibacterial effect of compound **2i** may result from its ability to disrupt biological membranes or induce oxidative stress, leading to the loss of cellular integrity. Consequently, the lack of selectivity suggests that further structural optimization and mechanistic studies are required to explore the compound’s safety profile and mechanism of action toward microbial and cancer cells.

#### 2.2.3. Effect on the AGE2-BSA/sRAGE Interaction

Targeting signaling pathways involving advanced glycation end-products (AGEs) and their receptor RAGE has recently emerged as a promising therapeutic approach for the treatment of diseases associated with the AGEs-RAGE axis, such as diabetes, atherosclerosis, neurodegenerative diseases, and obesity [56,57]. Moreover, the RAGE/ligand binding results in either downregulation or upregulation of key signaling pathways that are essential for proliferation of tumor cells, angiogenesis, and invasion [58]. Thus, AGEs-RAGE interaction triggers the mitogen-activated protein kinase (MAPK), nuclear factor kappa B (NF-κB) or phosphatidylinositide 3-kinase (PI3K)/protein kinase B (Akt) signaling pathways, which play a vital role in malignant progression of various types of tumors, including oral, colon, gastric, breast, prostate, ovarian, and cervical cancers [59,60,61,62,63,64,65,66]. Therefore, inhibiting the interaction between AGEs and RAGE could not only serve as a therapeutic target for chronic diseases, but also offer a promising approach for cancer treatment [43,58]. In this context, it seemed interesting to investigate the impact of a new class of antiproliferative compounds on RAGE/ligand interaction, especially since, despite significant research on drugs targeting RAGE inhibition, progress in their development is rather limited [49,67]. The effect of selected *N*-vinylindoles **2b**, **2d**-**f** and **2h**-**i** on the interaction between AGE2 (glyceraldehyde-modified AGE) and soluble RAGE (sRAGE) (AGE2-BSA/sRAGE) is reported in Table 3.

In general, the tested compounds displayed weak to moderate inhibition of the AGE2-BSA/sRAGE interaction (inhibition ranging from 12% to 49.4%). The most active compounds, **2f** and **2i**, possessed 4-Br-phenyl (compound **2f**, inhibition of 46.6%) or 2-naphthyl (compound **2i**, inhibition of 49.4%) substituent R, respectively. When a bromine atom at position 4 of the phenyl ring (compound **2f**, R = 4-Br-C_6_H_4_) or 2-naphthyl group (compound **2i**, R = 2-naphthyl) was substituted with a chlorine atom (compound **2e**, R = 4-Cl-C_6_H_4_) or 1-naphthyl moiety (compound **2h**, R = 1-naphthyl), respectively, the activity was slightly reduced (compound **2e**, inhibition of 34.7% and compound **2h**, inhibition of 36.3%). A noticeable decrease in potency was observed when a bromine atom at position 4 of the phenyl ring in compound **2f** was replaced with a fluorine atom in analog **2d** (inhibition of 46.6% vs. inhibition of 12%). Interestingly, derivative **2b** with a methyl group at position 4 of the phenyl ring (R = 4-CH_3_-C_6_H_4_) was devoid of activity. Thus the following trend was noticed for substituents R: 2-naphthyl (**2i**) ≥ 4-Br-C_6_H_4_ (**2f**) > 1-naphthyl (**2h**) ≥ 4-Cl-C_6_H_4_ (**2e**) > 4-F-C_6_H_4_ (**2d**) >> 4-CH_3_-C_6_H_4_ (**2b**). This suggests that the nature of a substituent at position 4 of the phenyl ring along with the steric properties of substituents R may affect the inhibitory efficiency of the tested compounds, and the presence of R substituents, such as 4-Br-C_6_H_4_, 4-Cl-C_6_H_4_, 1-naphthyl, and 2-naphtyl appear to be more favorable for the inhibition activity of this class of compounds.

Regarding the anticancer potential of the examined compounds, it is worth noting that the most potent derivatives **2f** and **2i** toward cancer cell lines (IC_50_ = 2.37–11.12 μg/mL and IC_50_ = 2.03–5.58 μg/mL, respectively) also demonstrated the highest activity against AGE2-BSA/sRAGE interaction (inhibition of 46.6% and 49.4%, respectively). On the other hand, compounds **2b** and **2d,** which showed good activity toward cancer cells (IC_50_ = 8.19–16.23 μg/mL and 8.65–15.48 μg/mL, respectively), exhibited lack (compound **2b**) or modest potency (compound **2d**, inhibition of 12%) on the AGE2-BSA/sRAGE interaction, while the least active on the cancer cells (IC_50_ = 10.09–18.90 μg/mL), compound **2h**, was found to be more potent against AGE2-BSA/sRAGE than **2b** and **2d** (inhibition of 36.4%).

From comparison of the relevant data, it can be assumed that there is no direct correlation between anticancer and AGE2-BSA/sRAGE inhibitory properties. The exception to this is compounds **2f** and **2i**. Although *N*-vinylindoles **2f** and **2i** displayed moderate RAGE-inhibitory properties, their pronounced cytotoxic activity against cancer cell lines suggests their potential as candidates for further development of anticancer agents targeting the AGE/RAGE pathway.

## 3. Materials and Methods

### 3.1. Experimental Section

#### 3.1.1. General Information

All chemicals were purchased from Sigma-Aldrich (Schellendorf, Germany), Merck (Darmstadt, Germany), Chemat (Gdańsk, Poland) or Avantor Performance Materials Poland S.A. (Gliwice, Poland), and used without further purification.

Melting points were determined using a Boetius apparatus (VEB Analytik Dresden, using a Nicolet 380 FT-IR 1600 spectrometer (Thermo, Waltham, MA, USA). ^1^H- and ^13^C-NMR spectra were recorded on a Varian Inova 500 (500 MHz) spectrometer (, Agilent, Santa Clara, CA, USA) and Bruker Avance III HD 400 (400 MHz) spectrometer (Billerica, MA, USA) using deuterated dimethyl sulfoxide (DMSO-*d*_6_) as the solvent. The values of ^1^H and ^13^C chemical shifts (δ) are expressed in parts per million (ppm), referenced to the residual solvent signals at 2.50 ppm and 39.5 ppm (DMSO-*d*_6_). Coupling constants (*J*) are reported in hertz (Hz). Mass spectra were obtained using a Shimadzu LCMS-2010 EV spectrometer (Tokyo, Japan) with an electrospray ionization (ESI) source. ESI-MS spectra were acquired in positive ionization modes.

Elemental analyses for carbon, hydrogen, and nitrogen were found to be within ±0.4% of the calculated theoretical values for all compounds.

Preparative thin-layer chromatography was performed on silica gel 60 PF254 containing gypsum (Merck KGaA, Darmstadt, FRG) with the aid of a Chromatotron^®^ (Harrison Research, CA, USA). Thin-layer chromatography (TLC) was carried out on Merck silica gel 60 F254, and the spots were visualized with UV light at λ = 254 nm.

1-[(4,5-dihydro-1*H*-imidazol-2-yl)methyl]-1*H*-indole (**1**) was prepared following a previously reported procedure [41].

The reported yields refer to the amounts obtained following the isolation and purification of the compounds.

#### 3.1.2. General Procedure for the Synthesis of (Z)-1-[2-aryl-1-(4,5-dihydro-1H-imidazol-2-yl)vinyl]-1H-indoles 2a-f

To a stirred solution of 1-[(4,5-dihydro-1*H*-imidazol-2-yl)methyl]-1*H*-indole (**1**) (0.299 g, 1.5 mmol) in chloroform (5 mL), 1*H*-benzotriazole was added (0.214 g, 1.8 mmol) and the reaction mixture was stirred for 30 min at ambient temperature (20–22 °C). After this time the appropriate aryl aldehyde (1.8 mmol) was added. The stirring was continued for 30 min at ambient temperature, and then under reflux for 6–12 h (TLC control using methanol/methylene chloride 0.5:9.5, *v*/*v* as the eluent). After this time, water was added (15 mL) to the resulting suspension, followed by extraction with methylene chloride (3 × 15 mL). The collected organic layers were dried with anhydrous sodium sulfate, filtered and evaporated under vacuum. The oily residue thus obtained was separated through preparative thin-layer chromatography using methanol/methylene chloride (0.5:9.5, *v*/*v*) as eluent. The unreacted aromatic aldehyde was eluted first followed by the product. The obtained product was subjected to crystallization from a suitable solvent if necessary. According to this procedure, the following title compounds were obtained.

(*Z*)-1-[1-(4,5-dihydro-1*H*-imidazol-2-yl)-2-(phenyl)vinyl]-1*H*-indole (**2a**)

For the reaction, 0.191 g (0.182 mL, 1.8 mmol) of benzaldehyde was used. Yield: 0.129 g (30%); m.p. 156–158 °C (acetone), white/beige powder; IR (KBr) v [cm^−1^]: 3180, 3064, 2923, 2856, 1645, 1613, 1565, 1512, 1475, 1461, 1407, 1357, 1319, 1267, 1222, 1194, 987, 927, 885, 758, 739, 689; ^1^H-NMR (DMSO-*d_6_*, 400 MHz) δ [ppm]: 3.54 (s, 4H, CH_2_), 6.63 (d, *J* = 3.2 Hz, 1H, CH), 6.70–6.72 (m, 2H, CH), 6.86 (br s, 1H, NH), 7.00–7.03 (m, 2H, CH), 7.11–7.14 (m, 2H, CH), 7.18–7.20 (m, 2H, CH), 7.52 (s, 1H, CH), 7.59–7.61 (m, 1H, CH); ^13^C-NMR (DMSO-*d_6_*, 100 MHz) δ [ppm]: 50.4 (two overlapping signals), 111.1, 120.1, 120.9, 122.1, 127.6, 128.7, 129.0 (two overlapping signals), 129.2, 129.4 (three overlapping signals), 129.5, 131.8, 133.5, 135.4, 163.5; MS (ESI, CH_3_OH:CH_3_CN + 0.1% CH_3_COOH, 1:1, *v*/*v*) *m*/*z* = 288 [M + H]^+^. Calculated for C_19_H_17_N_3_ (287.36): C, 79.41; H, 5.96; N, 14.62. Found: C, 79.34; H, 5.92; N, 14.74.

(*Z*)-1-[1-(4,5-dihydro-1*H*-imidazol-2-yl)-2-(*p*-tolyl)vinyl]-1*H*-indole (**2b**)

For the reaction, 0.216 g (0.212 mL, 1.8 mmol) of 4-methylbenzaldehyde was used. Yield: 0.140 g (31%); m.p. 184–186 °C (acetonitrile), white/beige powder; IR (KBr) v [cm^−1^]: 3146, 3058, 2976, 2922, 2855, 1648, 1609, 1565, 1514, 1476, 1461, 1418, 1319, 1269, 1220, 1194, 987, 885, 813, 736, 710; ^1^H-NMR (DMSO-*d_6_*, 400 MHz) δ [ppm]: 2.17 (s, 3H, CH_3_), 3.54 (br s, 4H, CH_2_), 6.58 (d, *J* = 8.3 Hz, 2H, CH), 6.62 (d, *J* = 3.2 Hz, 1H, CH), 6.67 (br s, 1H, NH), 6.93–6.97 (m, 3H, CH), 7.01–7.04 (m, 2H, CH), 7.18 (d, *J* = 3.1 Hz, 1H, CH), 7.48 (s, 1H, CH), 7.58–7.62 (m, 1H, CH); ^13^C-NMR (DMSO-*d_6_*, 100 MHz) δ [ppm]: 21.3, 120.1, 120.9, 122.1, 126.8, 128.6, 129.2, 129.4 (two overlapping signals), 129.6 (two overlapping signals), 130.7, 131.8, 135.4, 139.4, 163.6; MS (ESI, CH_3_OH:CH_3_CN + 0.1% CH_3_COOH, 1:1, *v*/*v*) *m*/*z* = 302 [M + H]^+^. Calculated for C_20_H_19_N_3_ (301.38): C, 79.70; H, 6.35; N, 13.94. Found: C, 79.44; H, 6.54; N, 14.02.

(*Z*)-1-[1-(4,5-dihydro-1*H*-imidazol-2-yl)-2-(4-methoxyphenyl)vinyl]-1*H*-indole (**2c**)

For the reaction, 0.245 g (0.219 mL, 1.8 mmol) of 4-methoxybenzaldehyde was used. Yield: 0.129 g (27%); m.p. 162–164 °C (acetonitrile), beige powder; IR (KBr) v [cm^−1^]: 3182, 3059, 2928, 2858, 2835, 1646, 1605, 1568, 1512, 1459, 1369, 1306, 1257, 1219, 1179, 1033, 984, 886, 828, 741, 729; ^1^H-NMR (DMSO-*d_6_*, 400 MHz) δ [ppm]: 3.52 (br s, 4H, CH_2_), 3.66 (s, 3H, OCH_3_), 6.60–6.63 (m, 4H, CH and NH), 6.70 (d, *J* = 9.0 Hz, 2H, CH), 6.96–6.98 (m, 1H, CH), 7.02–7.05 (m, 2H, CH), 7.19 (d, *J* = 3.2 Hz, 1H, CH), 7.47 (s, 1H, CH), 7.60–7.62 (m, 1H, CH); ^13^C-NMR (DMSO-*d_6_*, 100 MHz) δ [ppm]: 55.6, 103.1, 111.0, 114.5 (two overlapping signals), 120.1, 120.9, 122.1, 125.4, 125.9, 128.6, 129.2, 131.2 (two overlapping signals), 131.7, 135.4, 160.3, 163.7; MS (ESI, CH_3_OH:CH_3_CN + 0.1% CH_3_COOH, 1:1, *v*/*v*) *m*/*z* = 318 [M + H]^+^. Calculated for C_20_H_19_N_3_O (317.38): C, 75.69; H, 6.03; N, 13.24. Found: C, 75.58; H, 6.08; N, 13.02.

(*Z*)-1-[1-(4,5-dihydro-1*H*-imidazol-2-yl)-2-(4-fluorophenyl)vinyl]-1*H*-indole (**2d**)

For the reaction, 0.223 g (0.193 mL, 1.8 mmol) of 4-fluorobenzaldehyde was used Yield: 0.165 g (36%); m.p. 186–188 °C (2-propanol), white/beige powder; IR (KBr) v [cm^−1^]: 3142, 3011, 2923, 2861, 1650, 1612, 1660, 1567, 1510, 1475, 1460, 1301, 1268, 1232, 1218, 1195, 1163, 987, 829, 762, 738; ^1^H-NMR (DMSO-*d_6_*, 400 MHz) δ [ppm]: 3.55 (br s, 4H, CH_2_), 6.64 (d, *J* = 4.0 Hz, 1H, CH), 6.67 (br s, 1H, NH), 6.72–6.75 (m, 2H, CH), 6.94–7.06 (m, 5H, CH), 7.20 (d, *J* = 4.2 Hz, 1H, CH), 7.58–7.62 (m, 1H, CH); ^13^C-NMR (DMSO-*d_6_*, 100 MHz) δ [ppm]: 103.5, 111.0, 115.9, 116.1, 120.2, 121.0, 122.2, 127.4 (d, *J*_C-F_ = 2.0 Hz), 128.7, 129.1, 130.1 (d, *J*_C-F_ = 3.3 Hz), 130.4, 131.6 (d, *J*_C-F_ = 8.5 Hz, two overlapping signals), 135.3, 161.2, 163.6 (d, *J*_C-F_ = 25.9 Hz); MS (ESI, CH_3_OH:CH_3_CN + 0.1% CH_3_COOH, 1:1, *v*/*v*) *m*/*z* = 306 [M + H]^+^. Calculated for C_19_H_16_FN_3_ (305.35): C, 74.74; H, 5.28; N, 13.76. Found: C, 74.64; H, 5.38; N, 13,62.

(*Z*)-1-[2-(4-chlorophenyl)-1-(4,5-dihydro-1*H*-imidazol-2-yl)vinyl]-1*H*-indole (**2e**)

For the reaction, 0.253 g (1.8 mmol) of 4-chlorobenzaldehyde was used. Yield: 0.198 g (41%); m.p. 198–201 °C (acetonitrile), beige powder; IR (KBr) v [cm^−1^]: 3147, 3015, 2979, 2922, 2858, 1648, 1612, 1563, 1510, 1491, 1476, 1460, 1415, 1367, 1317, 1267, 1220, 1195, 1090, 1013, 987, 885, 822, 737, 709; ^1^H-NMR (DMSO-*d_6_*, 400 MHz) δ [ppm]: 3.55 (br s 4H, CH_2_), 6.62–6.63 (m, 1H, CH), 6.69–6.71 (m, 3H, NH, CH), 6.92–6.96 (m, 1H, CH), 7.02–7.05 (m, 2H, CH), 7.20–7.22 (m, 3H, CH), 7.49 (br s, 1H, CH), 7.58–7.62 (m, 1H, CH); ^13^C-NMR (DMSO-*d_6_*, 100 MHz) δ [ppm]: 103.6, 111.1, 120.3, 121.0, 122.2, 128.3, 128.7, 129.0 (two overlapping signals), 129.1, 130.1, 131.0 (two overlapping signals), 132.5, 133.9, 135.2, 163.4; MS (ESI, CH_3_OH:CH_3_CN + 0.1% CH_3_COOH, 1:1, *v*/*v*) *m*/*z* = 322, and 324 [M + H]^+^. Calculated for C_19_H_16_ClN_3_ (321.80): C, 70.91; H, 5.01; N, 13.06. Found: C, 70.82; H, 5.12; N, 12.98.

(*Z*)-1-[2-(4-bromophenyl)-1-(4,5-dihydro-1*H*-imidazol-2-yl)vinyl]-1*H*-indole (**2f**)

For the reaction, 0.333 g (1.8 mmol) of 4-bromobenzaldehyde was used. Yield: 0.171 g (31%); m.p. 147–149 °C (2-propanol), beige powder; IR (KBr) v [cm^−1^]: 3178, 2963, 2922, 2859, 1646, 1612, 1566, 1509, 1488, 1460, 1413, 1318, 1266, 1220, 1194, 1077, 1010, 985, 885, 817, 737, 706; ^1^H-NMR (DMSO-*d_6_*, 400 MHz) δ [ppm]: 3.35 (s, 4H, CH_2_), 6.62–6.65 (m, 3H, CH), 6.70 (br s, 1H, NH), 6.92–6.96 (m, 1H, CH), 7.01–7.06 (m, 2H, CH), 7.20 (d, *J* = 3.2 Hz, 1H, CH), 7.34 (d, *J* = 8.6 Hz, 2H, CH), 7.47 (s, 1H, CH), 7.58–7.61 (m, 1H, CH); ^13^C-NMR (DMSO-*d_6_*, 100 MHz) δ [ppm]: 103.6, 111.1, 120.3, 121.0, 122.2, 122,7, 128.4, 128.7, 129.1, 130.2, 131.2 (two overlapping signals), 132.0 (two overlapping signals), 132.9, 135.2, 163.4; MS (ESI, CH_3_OH:CH_3_CN + 0.1% CH_3_COOH, 1:1, *v*/*v*) *m*/*z* = 366, and 368 [M + H]^+^. Calculated for C_19_H_16_BrN_3_ (366.25): C, 62.31; H, 4.40; N, 11.47. Found: C, 62.22; H, 4.44; N, 11.38.

(*Z*)-1-[1-(4,5-dihydro-1*H*-imidazol-2-yl)-2-(4-nitrophenyl)vinyl]-1*H*-indole (**2g**)

For the reaction, 0.272 g (1.8 mmol) of 4-nitrobenzaldehyde was used. Yield: 0.110 g (22%); m.p. 192–194 °C (acetonitrile), orange powder; IR (KBr) v [cm^−1^]: 3114, 3083, 2971, 2932, 2858, 1642, 1593, 1568, 1514, 1460, 1343, 1316, 1269, 1219, 1194, 1110, 1060, 987, 914, 849, 742; ^1^H-NMR (DMSO-*d_6_*, 400 MHz) δ [ppm]: 3.59 (s, 4H, CH_2_), 6.66 (d, *J* = 2.8 Hz, 1H, CH), 6.82 (br s, 1H, NH), 6.94–6.96 (m, 3H, CH), 6.99–7.06 (m, 2H, CH), 7.23 (d, *J* = 3.3 Hz, 1H, CH), 7.60 (s, 1H, CH), 7.61–7.62 (m, 1H, CH), 7.98 (d, *J* = 8.9 Hz, 2H, CH); ^13^C-NMR (DMSO-*d_6_*, 100 MHz) δ [ppm]: 104.1, 111.1, 120.5, 121.1, 122.4, 124.0 (two overlapping signals), 128.6, 128.9, 129.2, 130.2 (two overlapping signals), 130.8, 135.2, 140.5, 147.2, 163.1; ^1^H-NMR (DMSO-*d_6_* + TFA, 500 MHz) δ [ppm]: 3.97 (s, 4H, CH_2_), 6.82 (d, *J* = 3.3 Hz, 1H, CH), 6.89 (d, *J* = 8.8 Hz, 2H, CH), 7.04–7.12 (m, 3H, CH), 7.37 (d, *J* = 3.3 Hz, 1H, CH), 7.66 (d, *J* = 7.1 Hz, 1H, CH), 8.01 (d, *J* = 8.8 Hz, 2H, CH), 8.04 (s, 1H, CH), 10.43 (s, 2H, NH); ^13^C-NMR (DMSO-*d_6_* + TFA, 125 MHz) δ [ppm]: 45.4 (two overlapping signals), 106.7, 110.9, 121.6 two overlapping signals), 123.1, 123.4, 124.2 (two overlapping signals), 128.3, 129,4, 131.2 (two overlapping signals), 134.9, 136.7, 138.0, 148.3, 164.1; MS (ESI, CH_3_OH:CH_3_CN + 0.1% CH_3_COOH, 1:1, *v*/*v*) *m*/*z* = 333 [M + H]^+^, and *m*/*z* = 374 [M + CH_3_CN]^+^. Calculated for C_19_H_16_N_4_O_2_ (332.36): C, 68.66; H, 4.85; N, 16.86. Found: C, 68.72; H, 4.82; N, 16.92.

(*Z*)-1-[1-(4,5-dihydro-1*H*-imidazol-2-yl)-2-(naphthalen-1-yl)vinyl]-1*H*-indole (**2h**)

For the reaction, 0.281 g (1.8 mmol) of naphthalene-1-carbaldehyde was used. Yield: 0.175 g (35%); m.p. 76–78 °C, dark-beige powder; IR (KBr) v [cm^−1^]: 3056, 2933, 2870, 1637, 1610, 1573, 1510, 1458, 1364, 1308, 1286, 1216, 1011, 799, 777, 745; ^1^H-NMR (DMSO-*d_6_*, 400 MHz) δ [ppm]: 3.63 (s, 4H, CH_2_), 6.49 (d, *J* = 3.1 Hz, 1H, CH), 6.60 (d, *J* = 7.3 Hz, 1H, CH), 6.84–6.94 (m, 3H, CH and NH), 7.07 (t, *J* = 7.8 Hz, 1H, CH), 7.19 (d, *J* = 3.1 Hz, 1H, CH), 7.43–7.45 (m, 1H, CH), 7.49 (d, *J* = 7.7 Hz, 1H, CH), 7.55–7.59 (m, 1H, CH), 7.63–7.66 (m, 1H, CH), 7.73 (d, *J* = 8.2 Hz, 1H, CH), 7.89–7.93 (m, 2H, CH), 8.08 (s, 1H, CH), 8.31 (d, *J* = 8.4 Hz, 1H, CH); ^13^C-NMR (DMSO-*d_6_*, 100 MHz) δ [ppm]: 103.0, 111.0, 115.4, 120.0, 120.7, 121.9, 124.4, 125.6, 125.7, 125.9, 126.6, 127.3, 128.3, 128.5, 129.0, 129.4, 129.7, 130.1, 130.7, 133.5 163.4; MS (ESI, CH_3_OH:CH_3_CN + 0.1% CH_3_COOH, 1:1, *v*/*v*) *m*/*z* = 338 [M + H]^+^. Calculated for C_23_H_19_N_3_ (337.42): C, 81.87; H, 5.68; N, 12.45. Found: C, 81.94; H, 5.58; N, 12.48.

(*Z*)-1-[1-(4,5-dihydro-1*H*-imidazol-2-yl)-2-(naphthalen-2-yl)vinyl]-1*H*-indole (**2i**)

For the reaction, 0.281 g (1.8 mmol) of naphthalene-2-carbaldehyde was used. Yield: 0.142 g (28%); m.p. 131–133 °C (ethyl acetate/diethyl ether), beige powder; IR (KBr) v [cm^−1^]: 3151, 2924, 2861, 1649, 1612, 1589, 1567, 1509, 1475, 1460, 1419, 1402, 1302, 1269, 1232, 1219, 1195, 1010, 829, 762, 739, 720; ^1^H-NMR (DMSO-*d_6_*, 400 MHz) δ [ppm]: 3.58 (s, 4H, CH_2_), 6.64 (dd, *J*_1_ = 1.5 Hz, *J*_2_ = 8.8 Hz, 1H, CH), 6.67 (d, *J* = 3.2 Hz, 1H, CH), 6.99–7.04 (m, 3H, CH and NH), 7.27 (d, *J* = 3.2 Hz, 1H, CH), 7.45–7.48 (m, 2H, CH), 7.52–7.54 (m, 2H, CH), 7.62–7.68 (m, 3H, CH), 7.73–7.75 (m, 1H, CH); ^13^C-NMR (DMSO-*d_6_*, 100 MHz) δ [ppm]: 103.5, 111.1, 120.2, 120.9, 122.2, 125.5, 127.0, 127.5, 127.7, 127.8, 128.3, 128.6, 128.7, 129.4, 130.4, 131.2, 131.8, 133.0, 133.2, 135.6, 163.6; MS (ESI, CH_3_OH:CH_3_CN + 0.1% CH_3_COOH, 1:1, *v*/*v*) *m*/*z* = 338 [M + H]^+^. Calculated for C_23_H_19_N_3_ (337.42): C, 81.87; H, 5.68; N, 12.45. Found: C, 81.68; H, 5.76; N, 12.56.

#### 3.1.3. Single-Crystal X-Ray Diffraction Experiments

Diffraction experiments were carried out at room temperature with an Oxford Diffraction Xcalibur E diffractometer using Mo Kα radiation. Diffraction data were processed with CrysAlisPro software [68]. The structure was solved with the program SHELXT [69] and refined by full-matrix least-squares method on F^2^ with SHELXL-2019/3 [70] using the Olex2 software [71]. Hydrogen atoms were placed in calculated positions and refined as riding on their carriers, except the N-H group H atom which was freely refined. The difference Fourier map showed several residual electron density peaks in the region of the indole substituent. A careful analysis indicated that in the crystal this substituent adopts two sites related approximately by 180° rotation around the C10-N1 bond. The occupancy factor of the minor position refined at 0.057(2). CCDC 2477393 contains the supplementary crystallographic data for this paper. These data can be obtained free of charge via http://www.ccdc.cam.ac.uk/conts/retrieving.html accessed on 1 September 2025 (or from the CCDC, 12 Union Road, Cambridge CB2 1EZ, UK; Fax: +44 1223 336033; E-mail: deposit@ccdc.cam.ac.uk).

Crystal data for **2g**: C_19_H_16_N_4_O_2_ (M =332.36 g/mol): monoclinic, space group *P2_1_/c* (no. 14), α = 16.4478(7) Å, *b* = 10.7316(4) Å, *c* = 9.8905(4) Å, *β* = 105.885(4)°, *V* = 1679.12(12) Å^3^, *Z* = 4, T = 295(2) K, μ(MoKα) = 0.089 mm^−1^, *D*_calc_ = 1.315 g/cm^3^, 24611 reflections measured (6.398° ≤ 2Θ ≤ 51.364°), 3175 unique (*R*_int_ = 0.0306, *R*_sigma_ = 0.0219) which were used in all calculations. The final *R*_1_ was 0.0458 (I > 2σ(I)) and *wR*_2_ was 0.1105 (all data).

### 3.2. Biological Studies

#### 3.2.1. Anticancer Studies

##### Cell Lines

The human ovarian SKOV-3, gastric adenocarcinoma AGS, cervical HeLa cell lines and non-cancerous keratinocytes HaCaT were obtained from the American Type Culture Collection (ATCC, Manassas, VA, USA). The cell lines were cultured in McCoy’s Medium, Dulbecco’s Modified Eagle’s Medium/F12, and DMEM, respectively (Merck Millipore, Burlington, MA, USA). Penicillin (100 units/mL), streptomycin (100 mg/mL), and 10% (*v*/*v*) fetal bovine serum (FBS) were added to all the media. The cells were incubated at 37 °C and 5% CO_2_.

##### MTT Assay

To estimate the cytotoxic activity of the obtained compounds **2a**-**i**, MTT assay was used. The cells (5 × 10^3^ cells/well) were treated with the compounds in a range of concentrations 1–150 µg/mL. Concentration of the compound’s solvent, DMSO (dimethyl sulfoxide), did not exceed 0.75% (*v*/*v*). After 24 h, MTT solution (0.5 mg/mL) was added to the cells. Formazan crystals were dissolved in DMSO and the absorbance was measured with a microtiter plate reader (Epoch, BioTek Instruments, Winooski, VT, USA). GraFit software v.7 (Erithacus Software, East Grinstead, West Sussex, UK) was used to analyze the obtained results which were expressed as IC_50_ values calculated from two independent experiments (in six repetitions, n = 12).

##### Caspase-3/7 Assay

The SKOV-3 cells (5 × 10^5^ cells/well) were seeded and incubated with compound **2i** at concentrations of 2, 5, 10, 15, 30, and 60 µg/mL for 24 h. Then, the cells were stained with Muse Caspase-3/7 Kit, according to the manufacturer’s instructions. Muse Cell Analyzer and Muse 1.4 Analysis Software (Merck Millipore, Burlington, MA, USA) were used to calculate the amount of live, early apoptotic, late apoptotic, and dead cells. The results are shown as a percentage of the cells in each population obtained from two independently repeated experiments (in three repeats).

##### Cell Cycle Assay

The SKOV-3 cells (5 × 10^5^ cells/well) were seeded and incubated with compound **2i** at concentrations of 2, 5, 10, 15, 30, and 60 µg/mL for 48 h. After treatment, Muse Cell Cycle Assay Kit and Muse Cell Analyzer (Merck Millipore, Burlington, MA, USA) were used to analyze the stained cells, according to the manufacturer’s instruction. The results are shown as percentage of the cells in each phase of cell cycle: subG0, G0/G1, S, and G2/M. The experiment was repeated in two independent repeats (in three repetitions).

##### Statistical Analysis

The STATISTICA 12.0 software package (StatSoft. Inc., Tulsa, OK, USA) was used in statistical analysis. The data relating to cytotoxic activity of the compounds are expressed as mean values ± standard deviation (±SD). To compare the results with the control sample, Student’s *t*-test was used (the statistical significance was set at *p* < 0.05).

#### 3.2.2. Microbroth Dilution Assay

The minimum inhibitory concentration (MIC) and minimum bactericidal/fungicidal concentration (MBC/MFC) of the tested compounds were determined by the microbroth dilution technique using 96-well plates based on the methodology described in the EUCAST (European Committee on Antimicrobial Susceptibility Testing) standards [72,73]. Reference and clinical strains of Gram-positive (*Staphylococcus aureus* ATCC 6538, *Staphylococuccus aureus* MRSA 6347) and Gram-negative (*Escherichia coli* ATCC 8739) bacteria, and yeast (*Candida albicans* ATCC 10231, *Candida glabrata* ATCC 2001) were used for the assay. The studies were carried out in the Mueller–Hinton (MH) broth for bacteria and RPMI 1640 broth + MOPS (Sigma-Aldrich, Poland) buffer in the case of yeast. The dry test samples were dissolved in DMSO (dimethyl sulfoxide), resulting in a final concentration of 15 mg/mL, and then diluted in a geometric progression. *Ampicillin* (Sigma-Aldrich, Poland) acted as a control sample (512 to 4 µg/mL) for bacteria and *ketoconazole* (128 to 1 µg/mL) (Sigma-Aldrich, Poland) acted as a control sample for yeast. The cultures of microorganisms were prepared by transferring cells from the stock cultures to tubes with the appropriate broth and incubated with agitation in an aerobic atmosphere for 18 ± 2 h at 35 ± 1 °C; next, they were diluted to achieve an optical density corresponding to 5 × 105 (bacteria) or 2 × 105 (yeast) colony-forming units per mL (cfu/mL). An appropriate media (96 µL), along with the tested compounds with a final concentration range of 300–3.1 µg/mL (4 µL) and next microbial suspension (100 µL) were added to each well. Plates with samples were incubated in adequate conditions described above and microbial growth was assessed. The MIC was considered as the lowest sample’s concentration that completely prevented visible growth of bacteria [72] (>90%; *ketoconazole*) but 50% (for other compounds) inhibits visible growth of yeasts [73]. Next, as a control of microbial viability, 100 µL of the samples from each well without growth was inoculated on the MH or RPMI agar plates and incubated for 18 ± 2 h at 35 ± 1 °C and finally, the CFU was counted. The MBC/MFC was determined as the minimum concentration of compounds required to kill 99.9% the organisms [74,75].

#### 3.2.3. ELISA AGE2-BSA/sRAGE Inhibitory Screening

The in vitro inhibitor screening of AGE2 (glyceraldehyde-modified AGE)-sRAGE interaction (soluble RAGE) was performed using a 96-well AGE-BSA-coated plate (Creative BioMart^®^ ELISA kit, Shirley, NY, USA), according to the manufacturer’s instructions. 100 UA/mL soluble RAGE (sRAGE) was incubated with 100 μM of the tested compounds or 0.05% DMSO as a negative control on an AGE2-BSA-coated plate at room temperature for 60 min, shaking at 300 rpm on an orbital microplate shaker. After incubation, the wells were washed 4 times with wash buffer and horseradish peroxidase (HRP)-labeled anti-RAGE antibody was added and the plate was further incubated at room temperature for 60 min, shaking at 300 rpm on an orbital microplate shaker. After incubation, the wells were washed 4 times with wash buffer and the Substrate Reagent was added to each well. After an incubation at room temperature for 5 min, the HRP-labeled antibody sRAGE-AGE complex was then detected by measuring the absorbance at 450 nm using the microplate reader Varioskan (Thermo Fischer Scientific). These experiments were repeated two times.

## 4. Conclusions

The facile one-pot synthesis of 1-[2-aryl-1-(4,5-dihydro-1*H*-imidazol-2-yl)vinyl]-1*H*-indoles **2a**-**i** was achieved by means of Knoevenagel condensation in the presence of 1*H*-benzotriazole, leading to a novel class of *N*-vinylindole derivatives. It is worth emphasizing that the development method enabled the synthesis of *N*-vinylindoles containing two heterocyclic systems at the α-position of the vinyl functionality. The structures of the newly prepared compounds were confirmed by IR, 1D NMR spectroscopy, and MS spectrometry. For compound **2g**, additional 2D NMR experiments were conducted. Moreover, X-ray crystallographic analysis of the representative *N*-vinylindole **2g** revealed the *Z* configuration at the double bond of the vinyl moiety.

The anticancer potential of all synthesized compounds was assessed in vitro against three human cancer cell lines: HeLa, SKOV-3, and AGS, as well as non-cancerous HaCaT cell line. In general, the tested compounds **2a**-**i** demonstrated significant growth inhibitory effect on the human cancer cell lines used, especially HeLa and SKOV-3. Their cytotoxicity toward these two cell lines was higher than the standard anticancer agent, *oxaliplatin* (IC_50_ = 2.03–10.39 μg/mL vs. IC_50_ = 35.76–102.16 μg/mL). Nevertheless, the tested derivatives also exhibited cytotoxic effects on the non-cancerous cell line, HaCaT. Among *N*-vinylindole derivatives **2a**-**i**, compounds **2f** and **2i** containing 4-bromophenyl or 2-naphthyl group as the R substituent, respectively, were found to be the most active. Particularly, the presence of the 2-napthyl group seems to be essential for the cytotoxic activity against cancer cell lines, as evidenced by compound **2i** with the highest cytotoxic effect on HeLa, SKOV-3, and AGS cell lines (IC_50_ = 2.03–5.68 μg/mL). Based on the results of the analysis of apoptosis and cell cycle inhibition in SKOV-3 ovarian cancer cells treated with derivative **2i**, it can be assumed that the apoptotic effect of **2i** on SKOV-3 cells results from cell cycle arrest in the subG0 phase. However, further work is needed to elucidate the mechanism of action leading to the cell death.

Interestingly, the presence of either 4-bromophenyl or 2-naphthyl groups as the R substituent also appears to be effective at inhibiting the AGE2-BSA/sRAGE interaction. This feature seems to be beneficial in terms of promising anticancer properties of *N*-vinylindoles **2f** and **2i**, and makes them of interest for further development of antitumor activity related to RAGE inhibition.

In turn, the results of microbiological tests revealed that the majority of the investigated compounds **2a**-**i** possess rather poor activities against the examined bacterial and yeast strains, with MIC, MBC or MFC values ranging from 110 to ≥240 μg/mL. On the other hand, the derivative **2i**, which demonstrated the highest potency toward the tested cancer cell lines, exhibited good antibacterial activity on *S. aureus* ATCC 6535 and MRSA 12673 strains (MIC = 15–30 μg/mL, MBC = 30 μg/mL). While the antiproliferative activity against cancer cells is more pronounced, the antibacterial effect can be considered an advantageous property, when the drug can also inhibit infections that may occur due to immunodeficiency caused by anticancer treatment. Furthermore, within the tested compounds, only *N*-vinylindole **2i** exhibited moderate antifungal activity against *C. albicans* (MIC and MFC = 60 μg/mL).

In summary, we have developed a facile method for the synthesis of a novel class of *N*-vinylindoles bearing an imidazoline scaffold at the α-position of the vinyl functionality. The results of our preliminary biological studies revealed that the effect on cancer cells and AGE/RAGE interaction from these types of compounds should be further developed. In particular, compound **2i** was proven to be a potential candidate for future development of anticancer agents that may address the RAGE target.

## Figures and Tables

**Table 1:** Cytotoxic effects of the investigated *N*-vinylindoles **2a**-**i** on human cancer cell and non-cancerous cell lines.

Compound	IC_50_ ± SD (μg/mL) ^a^			
	Cell Line			
	HeLa	SKOV-3	AGS	HaCaT
**2a**	8.96 ± 0.33	9.81 ± 0.45	17.25 ± 0.68	11.70 ± 1.31
**2b**	8.19 ± 0.40	9.69 ± 0.62	16.23 ± 1.20	12.09 ± 0.55
**2c**	7.86 ± 0.59	8.80 ± 0.46	10.29 ± 0.08	9.11 ± 1.58
**2d**	8.65 ± 0.68	10.25 ± 0.45	15.48 ± 0.72	7.37 ± 0.28
**2e**	3.13 ± 0.30	4.40 ± 0.16	11.37 ± 0.74	3.43 ± 0.05
**2f**	2.37 ± 0.15	2.76 ± 0.18	11.12 ± 1.24	3.13 ± 0.09
**2g**	5.60 ± 0.57	7.06 ± 0.62	10.14 ± 0.08	9.15 ± 0.74
**2h**	10.09 ± 1.12	10.39 ± 0.10	18.90 ± 1.81	17.95 ± 1.93
**2i**	2.03 ± 0.05	2.45 ± 0.13	5.68 ± 0.41	3.02 ± 0.18
***Oxaliplatin*** ^b^	35.76 ± 1.72	102.16 ± 9.26	17.90 ± 1.20	NT ^c^
***Vinblastine sulfate*** ^b^	0.005 ± 0.0004	0.008 ± 0.0003	NT ^c^	NT ^c^

^a^ The IC_50_ values were obtained with MTT assay from two experiments, in six repetitions (n = 12). ^b^ *Oxaliplatin* and *vinblastine sulfate* were used as positive controls. ^c^ NT—not tested.

**Table 2:** Antimicrobial activity of *N*-vinylindoles **2a**-**i** expressed as minimal inhibitory concentration (MIC) and minimal bactericidal/fungicidal concentration (MBC/MFC) against reference strains of bacteria and yeast ^a^.

Compound ^b^	Microorganism									
	***S. aureus*** ATCC 6535		***S. aureus*** MRSA 12673		***E. coli*** ATCC 8739		***C. albicans*** ATCC 10231		***C. glabrata*** ATCC 2001	
	MIC	MBC	MIC	MBC	MIC	MBC	MIC	MFC	MIC	MFC
	μg/mL									
**2a**	110	220	220	>220	220	220	>220	>220	>220	>220
**2b**	55	110	220	>220	220	>220	220	>220	220	>220
**2c**	110	110	>220	>220	220	>220	>220	>220	>220	>220
**2d**	120	240	120	>240	240	>240	>240	>240	>240	>240
**2e**	30	60	60	240	120	240	120	240	240	240
**2f**	27.5	55	55	110	110	110	110	110	220	220
**2g**	55	110	110	>220	220	>220	>220	>220	>220	>220
**2h**	120	240	120	240	>240	>240	>240	>240	240	240
**2i**	15	30	30	30	>240	>240	120	120	60	60
***Ampicillin***	8	8	512	512	128	128	-	-	-	-
***Ketoconazole***	-	-	-	-	-	-	>128	>128	64	128

^a^ The tests were performed in triplicate, and the results obtained were identical. ^b^ *Ampicillin* for bacteria, and *ketoconazole* for yeast were used as standards.

**Table 3:** AGE2-BSA/sRAGE interaction inhibition by *N*-vinylindoles **2b**, **2d**-**f**, and **2h**-**i** at a 100 µM concentration determined by ELISA assay ^a^.

Compound	R	Inhibition (%)
**2b**	4-CH_3_-C_6_H_4_	NA ^b^
**2d**	4-F-C_6_H_4_	12
**2e**	4-Cl-C_6_H_4_	34.7
**2f**	4-Br-C_6_H_4_	46.6
**2h**	1-naphthyl	36.3
**2i**	2-naphthyl	49.4

^a^ AGE2-BSA—a ligand of RAGE. ^b^ NA—non active compound.

## Data Availability

The original contributions presented in this study are included in the article/Supplementary Material. Further inquiries can be directed to the corresponding author.

## Supplementary Materials

The following supporting information can be downloaded at https://www.mdpi.com/article/10.3390/ijms262010149/s1.
