# Supplementary material for: Facile Synthesis of N-vinylindoles via Knoevenagel Condensation: Molecular Features and Biological Activities
Source: Int J Mol Sci. 2025 Oct 18;26(20):10149. doi: 10.3390/ijms262010149 (PMC12562379; doi:10.3390/ijms262010149)
Supplement: Supplementary file 1 [file ijms-26-10149-s001.zip › ijms-3921341-supplementary.pdf]

Supplementary Materials

Facile Synthesis of *N*-vinylindoles via Knoevenagel Condensation: Molecular Features and Biological Activities

Anita Kornicka [1,*], Justyna Stefanowicz-Hajduk [2], Katarzyna Turecka [3], Christophe Furman [4], Maria Gdaniec [5], and Łukasz Balewski

1 Department of Chemical Technology of Drugs, Faculty of Pharmacy, Medical University of Gdansk, Gen. J. Hallera 107, 80-416 Gdansk, Poland; anita.kornicka@gumed.edu.pl (A.K.); lukasz.balewski@gumed.edu.pl (Ł.B.)
2 Department of Biology and Pharmaceutical Botany, Faculty of Pharmacy, Medical University of Gdansk, Gen. J. Hallera 107, 80-416 Gdańsk, Poland; justyna.stefanowicz-hajduk@gumed.edu.pl (J.S.-H.)
3 Department of Pharmaceutical Microbiology, Faculty of Pharmacy, Medical University of Gdansk, Gen. J. Hallera 107, 80-416 Gdańsk, Poland; katarzyna.turecka@gumed.edu.pl (K.T.)
4 University of Lille, Inserm, CHU Lille, Institut Pasteur de Lille, UMR 1167—RID-AGE—Risk Factors and Molecular Determinants of Aging-Related Diseases, F-59000 Lille, France; chris-tophe.furman@univ-lille2.fr (C.F.)
5 Faculty of Chemistry, Adam Mickiewicz University, 61-614 Poznań, Poland; maria.gdaniec@amu.edu.pl (M.G.)

* Correspondence: lukasz.balewski@gumed.edu.pl (Ł.B.); anita.kornicka@gumed.edu.pl (A.K.)

TABLE OF CONTENTS

1. *IR, 1D and 2D NMR, and MS spectra of N-vinylindoles 2a-i*

Figure S1. IR of (*Z*)-1-[1-(4,5-dihydro-1*H*-imidazol-2-yl)-2-(phenyl)vinyl]-1*H*-indole (**2a**) **S4**
Figure S2. ^{1}H-NMR (400 MHz, DMSO-d_6) of (*Z*)-1-[1-(4,5-dihydro-1*H*-imidazol-2-yl)-2-(phenyl)vinyl]-1*H*-indole (**2a**) **S5**
Figure S3. ^{13}C-NMR (100 MHz, DMSO-d_6) of (*Z*)-1-[1-(4,5-dihydro-1*H*-imidazol-2-yl)-2-(phenyl)vinyl]-1*H*-indole (**2a**) **S6**
Figure S4. MS of (*Z*)-1-[1-(4,5-dihydro-1*H*-imidazol-2-yl)-2-(phenyl)vinyl]-1*H*-indole (**2a**) **S7**
Figure S5. IR of (*Z*)-1-[1-(4,5-dihydro-1*H*-imidazol-2-yl)-2-(*p*-tolyl)vinyl]-1*H*-indole (**2b**) **S8**
Figure S6. ^{1}H-NMR (400 MHz, DMSO-d_6) of (*Z*)-1-[1-(4,5-dihydro-1*H*-imidazol-2-yl)-2-(*p*-tolyl)vinyl]-1*H*-indole (**2b**) **S9**
Figure S7. ^{13}C-NMR (100 MHz, DMSO-d_6) of (*Z*)-1-[1-(4,5-dihydro-1*H*-imidazol-2-yl)-2-(*p*-tolyl)vinyl]-1*H*-indole (**2b**) **S10**
Figure S8. MS of (*Z*)-1-[1-(4,5-dihydro-1*H*-imidazol-2-yl)-2-(*p*-tolyl)vinyl]-1*H*-indole (**2b**) **S11**
Figure S9. IR of (*Z*)-1-[1-(4,5-dihydro-1*H*-imidazol-2-yl)-2-(4-methoxyphenyl)vinyl]-1*H*-indole (**2c**) **S12**
Figure S10. ^{1}H-NMR (400 MHz, DMSO-d_6) of (*Z*)-1-[1-(4,5-dihydro-1*H*-imidazol-2-yl)-2-(4-methoxyphenyl)vinyl]-1*H*-indole (**2c**) **S13**
Figure S11. ^{13}C-NMR (100 MHz, DMSO-d_6) of (*Z*)-1-[1-(4,5-dihydro-1*H*-imidazol-2-yl)-2-(4-methoxyphenyl)vinyl]-1*H*-indole (**2c**) **S14**
Figure S12. MS of (*Z*)-1-[1-(4,5-dihydro-1*H*-imidazol-2-yl)-2-(4-methoxyphenyl)vinyl]-1*H*-indole (**2c**) **S15**
Figure S13. IR of (*Z*)-1-[1-(4,5-dihydro-1*H*-imidazol-2-yl)-2-(4-fluorophenyl)vinyl]-1*H*-indole (**2d**) **S16**
Figure S14. ^{1}H-NMR (400 MHz, DMSO-d_6) of (*Z*)-1-[1-(4,5-dihydro-1*H*-imidazol-2-yl)-2-(4-fluorophenyl)vinyl]-1*H*-indole (**2d**) **S17**

Figure S15. ^{13}C-NMR (100 MHz, DMSO-d_6) of (*Z*)-1-[1-(4,5-dihydro-1*H*-imidazol-2-yl)-2-(4-fluorophenyl)vinyl]-1*H*-indole (**2d**) **S18**
Figure S16. MS of (*Z*)-1-[1-(4,5-dihydro-1*H*-imidazol-2-yl)-2-(4-fluorophenyl)vinyl]-1*H*-indole (**2d**) **S19**
Figure S17. IR of (*Z*)-1-[2-(4-chlorophenyl)-1-(4,5-dihydro-1*H*-imidazol-2-yl)vinyl]-1*H*-indole (**2e**) **S20**
Figure S18. ^{1}H-NMR (400 MHz, DMSO-d_6) of (*Z*)-1-[2-(4-chlorophenyl)-1-(4,5-dihydro-1*H*-imidazol-2-yl)vinyl]-1*H*-indole (**2e**) **S21**
Figure S19. ^{13}C-NMR (100 MHz, DMSO-d_6) of (*Z*)-1-[2-(4-chlorophenyl)-1-(4,5-dihydro-1*H*-imidazol-2-yl)vinyl]-1*H*-indole (**2e**) **S22**
Figure S20. MS of (*Z*)-1-[2-(4-chlorophenyl)-1-(4,5-dihydro-1*H*-imidazol-2-yl)vinyl]-1*H*-indole (**2e**) **S23**
Figure S21. IR of (*Z*)-1-[2-(4-bromophenyl)-1-(4,5-dihydro-1*H*-imidazol-2-yl)vinyl]-1*H*-indole (**2f**) **S24**
Figure S22. ^{1}H-NMR (400 MHz, DMSO-d_6) of (*Z*)-1-[2-(4-bromophenyl)-1-(4,5-dihydro-1*H*-imidazol-2-yl)vinyl]-1*H*-indole (**2f**) **S25**
Figure S23. ^{13}C-NMR (100 MHz, DMSO-d_6) of (*Z*)-1-[2-(4-bromophenyl)-1-(4,5-dihydro-1*H*-imidazol-2-yl)vinyl]-1*H*-indole (**2f**) **S26**
Figure S24. MS of (*Z*)-1-[2-(4-bromophenyl)-1-(4,5-dihydro-1*H*-imidazol-2-yl)vinyl]-1*H*-indole (**2f**) **S27**
Figure S25. IR of (*Z*)-1-[1-(4,5-dihydro-1*H*-imidazol-2-yl)-2-(4-nitrophenyl)vinyl]-1*H*-indole (**2g**) **S28**
Figure S26. ^{1}H-NMR (400 MHz, DMSO-d_6) of (*Z*)-1-[1-(4,5-dihydro-1*H*-imidazol-2-yl)-2-(4-nitrophenyl)vinyl]-1*H*-indole (**2g**) **S29**
Figure S27. ^{13}C-NMR (100 MHz, DMSO-d_6) of (*Z*)-1-[1-(4,5-dihydro-1*H*-imidazol-2-yl)-2-(4-nitrophenyl)vinyl]-1*H*-indole (**2g**) **S30**
Figure S28. ^{1}H-NMR (500 MHz, DMSO-d_6 + TFA) of (*Z*)-1-[1-(4,5-dihydro-1*H*-imidazol-2-yl)-2-(4-nitrophenyl)vinyl]-1*H*-indole (**2g/A**) **S31**
Figure S29. ^{13}C-NMR (125 MHz, DMSO-d_6 + TFA) of (*Z*)-1-[1-(4,5-dihydro-1*H*-imidazol-2-yl)-2-(4-nitrophenyl)vinyl]-1*H*-indole (**2g/A**) **S32**
Figure S30. HSQC (DMSO-d_6 + TFA) of (*Z*)-1-[1-(4,5-dihydro-1*H*-imidazol-2-yl)-2-(4-nitrophenyl)vinyl]-1*H*-indole (**2g/A**) **S33**
Figure S31. HMBC (DMSO-d_6 + TFA) of (*Z*)-1-[1-(4,5-dihydro-1*H*-imidazol-2-yl)-2-(4-nitrophenyl)vinyl]-1*H*-indole (**2g/A**) **S34**
Figure S32. MS of (*Z*)-1-[1-(4,5-dihydro-1*H*-imidazol-2-yl)-2-(4-nitrophenyl)vinyl]-1*H*-indole (**2g**) **S35**
Figure S33. IR of (*Z*)-1-[1-(4,5-dihydro-1*H*-imidazol-2-yl)-2-(naphthalen-1-yl)vinyl]-1*H*-indole (**2h**) **S36**
Figure S34. ^{1}H-NMR (400 MHz, DMSO-d_6) of (*Z*)-1-[1-(4,5-dihydro-1*H*-imidazol-2-yl)-2-(naphthalen-1-yl)vinyl]-1*H*-indole (**2h**) **S37**
Figure S35. ^{13}C-NMR (100 MHz, DMSO-d_6) of (*Z*)-1-[1-(4,5-dihydro-1*H*-imidazol-2-yl)-2-(naphthalen-1-yl)vinyl]-1*H*-indole (**2h**) **S38**
Figure S36. MS of (*Z*)-1-[1-(4,5-dihydro-1*H*-imidazol-2-yl)-2-(naphthalen-1-yl)vinyl]-1*H*-indole (**2h**) **S39**
Figure S37. IR of (*Z*)-1-[1-(4,5-dihydro-1*H*-imidazol-2-yl)-2-(naphthalen-2-yl)vinyl]-1*H*-indole (**2i**) **S40**
Figure S38. ^{1}H-NMR (100 MHz, DMSO-d_6) of (*Z*)-1-[1-(4,5-dihydro-1*H*-imidazol-2-yl)-2-(naphthalen-2-yl)vinyl]-1*H*-indole (**2i**) **S41**
Figure S39. ^{13}C-NMR (100 MHz, DMSO-d_6) of (*Z*)-1-[1-(4,5-dihydro-1*H*-imidazol-2-yl)-2-(naphthalen-2-yl)vinyl]-1*H*-indole (**2i**) **S42**

Figure S40. MS of (*Z*)-1-[1-(4,5-dihydro-1*H*-imidazol-2-yl)-2-(naphthalen-2-yl)vinyl]-1*H*-indole (**2i**) **S43**

2. ***X-ray Crystallographic Studies***

Figure S41. CheckCIF/PLATON report for (*Z*)-1-[1-(4,5-dihydro-1*H*-imidazol-2-yl)-2-(4-nitrophenyl)vinyl]-1*H*-indole (**2g**) **S44**

IR, 1D and 2D NMR, and MS spectra of N-vinylindoles 2a-i

Figure S1. IR of (*Z*)-1-[1-(4,5-dihydro-1*H*-imidazol-2-yl)-2-(phenyl)vinyl]-1*H*-indole (**2a**).

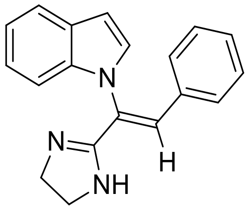

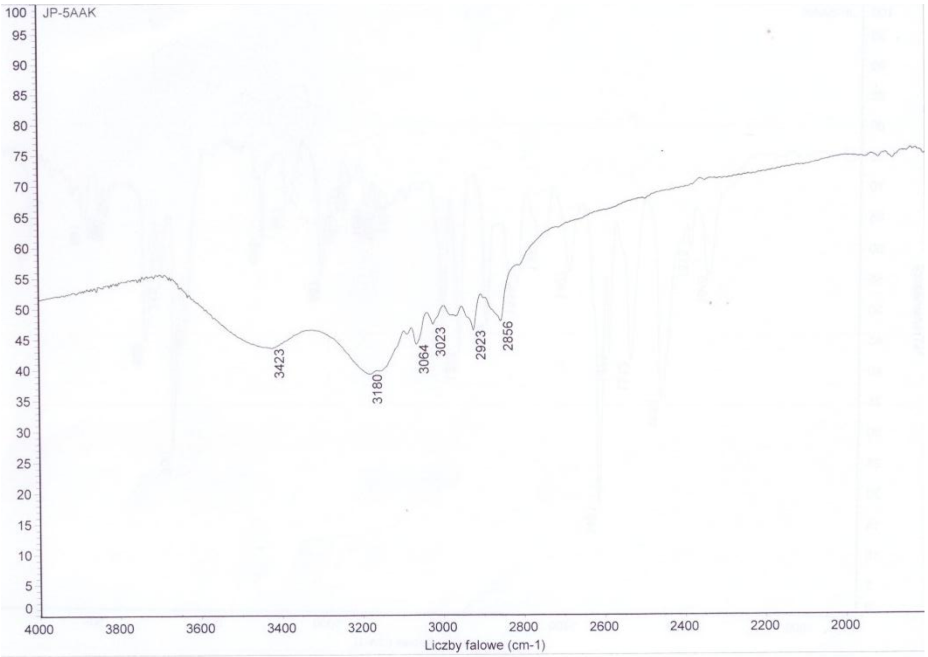

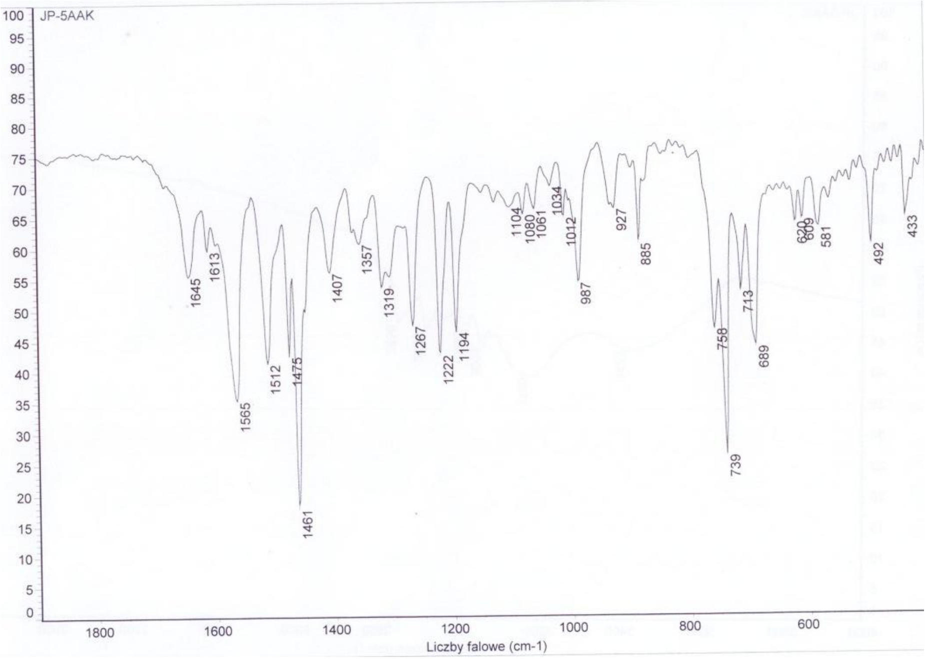

Figure S2. ^{1}H-NMR (400 MHz, DMSO-d_6) of (Z)-1-[1-(4,5-dihydro-1H-imidazol-2-yl)-2-(phenyl)vinyl]-1H-indole (**2a**).

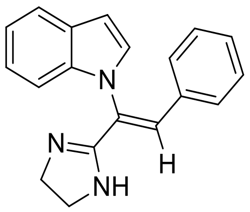

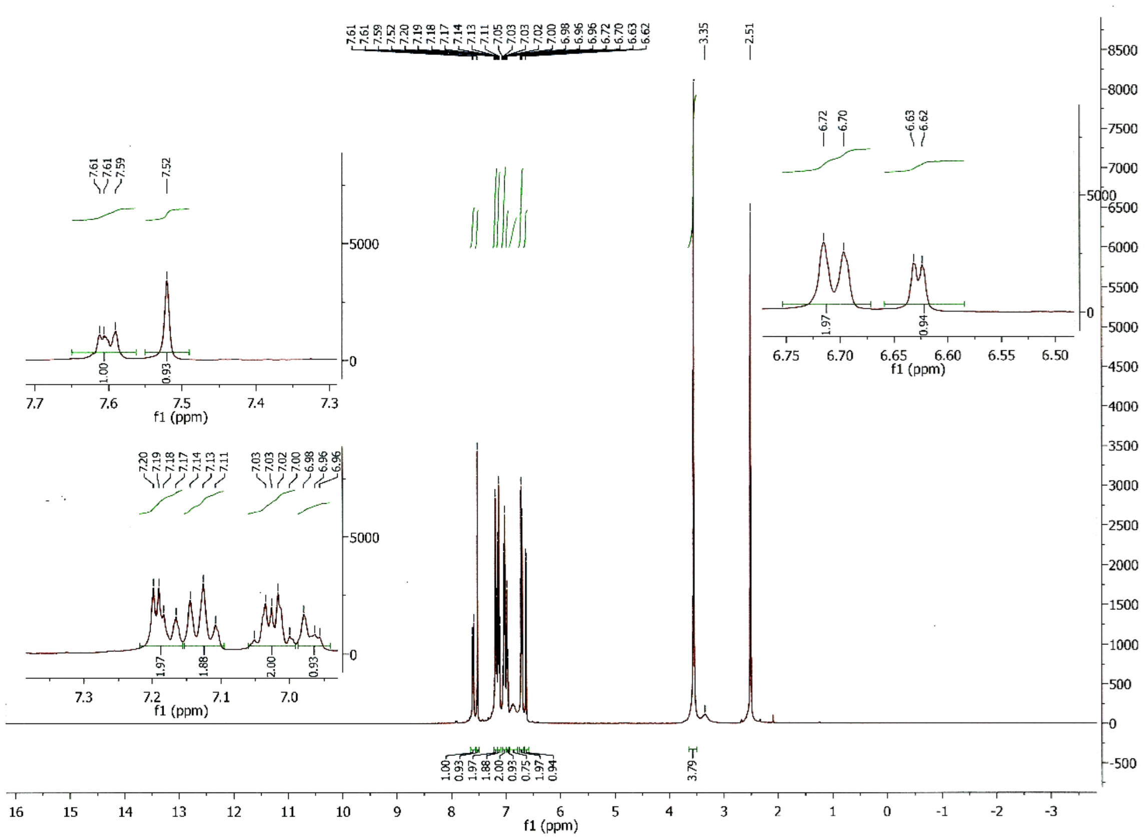

Figure S3. ^{13}C-NMR (100 MHz, DMSO-d_6) of (*Z*)-1-[1-(4,5-dihydro-1*H*-imidazol-2-yl)-2-(phenyl)vinyl]-1*H*-indole (**2a**).

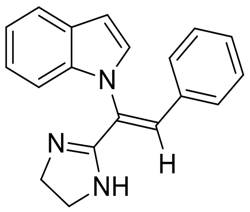

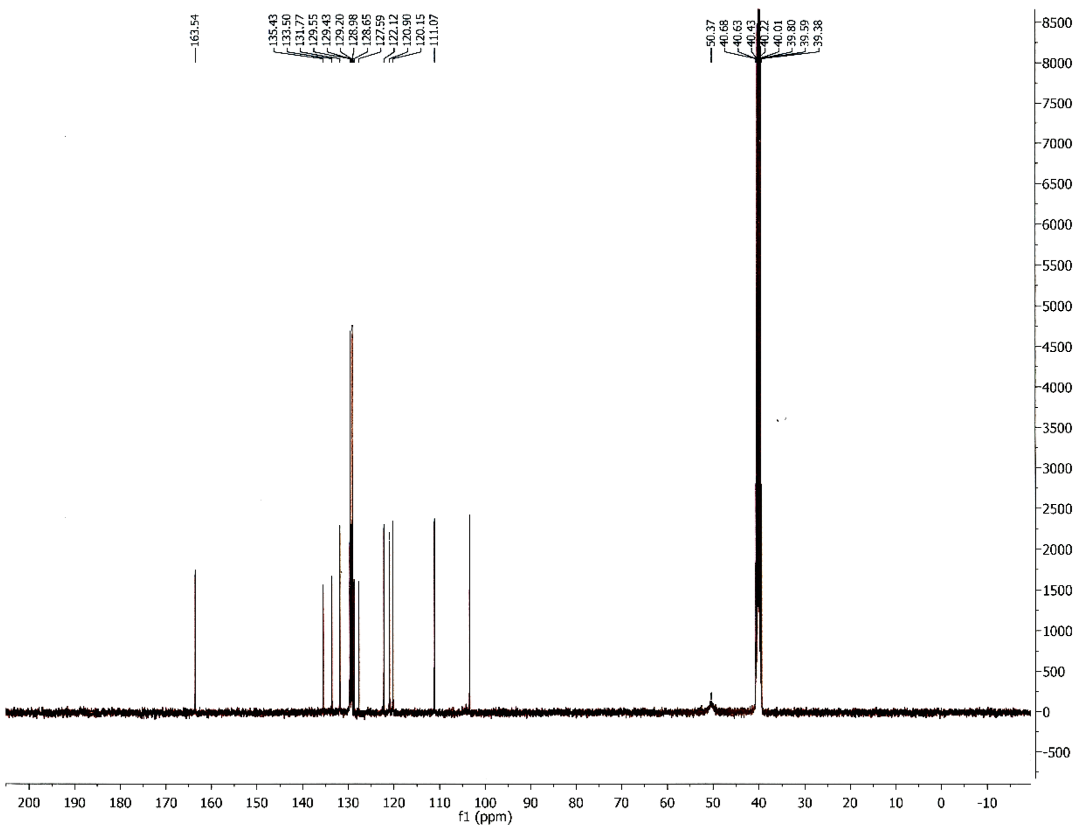

Figure S4. MS of (*Z*)-1-[1-(4,5-dihydro-1*H*-imidazol-2-yl)-2-(phenyl)vinyl]-1*H*-indole (**2a**).

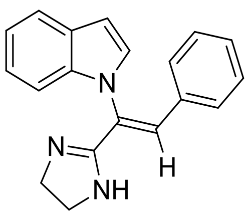

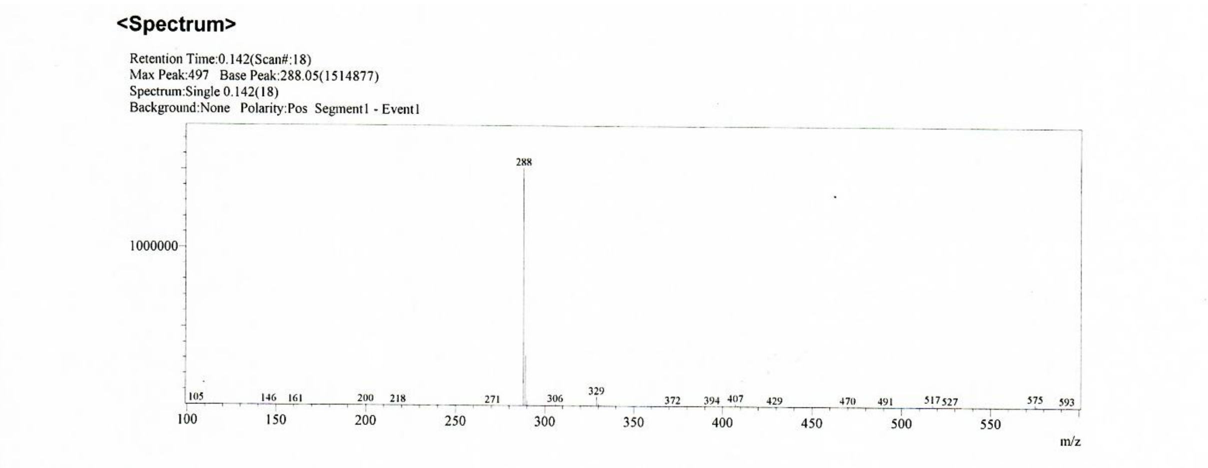

Figure S5. IR of (*Z*)-1-[1-(4,5-dihydro-1*H*-imidazol-2-yl)-2-(*p*-tolyl)vinyl]-1*H*-indole (**2b**).

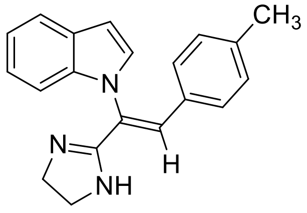

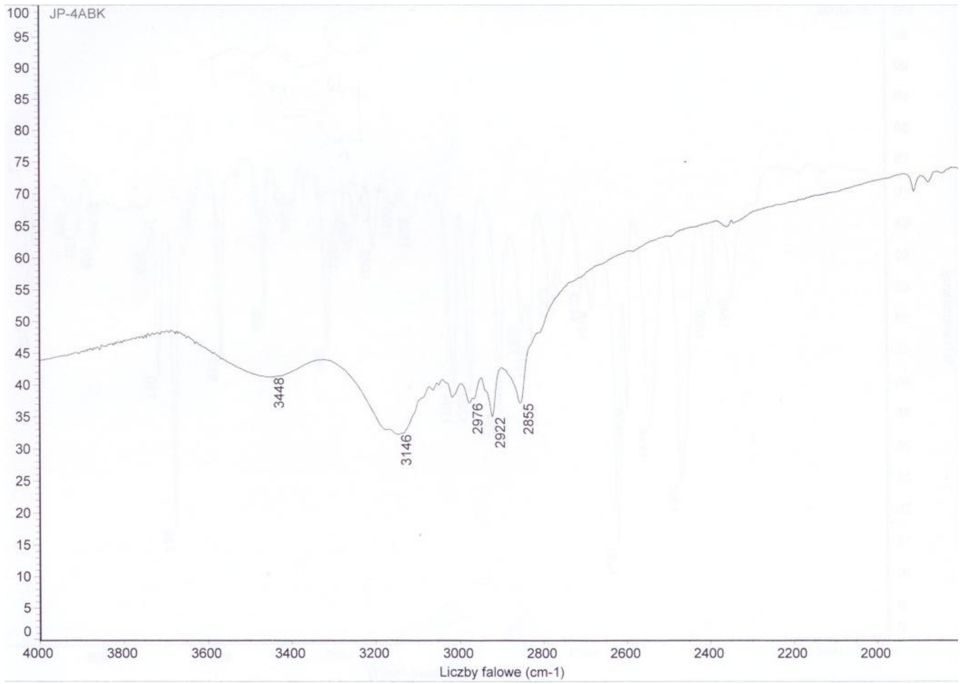

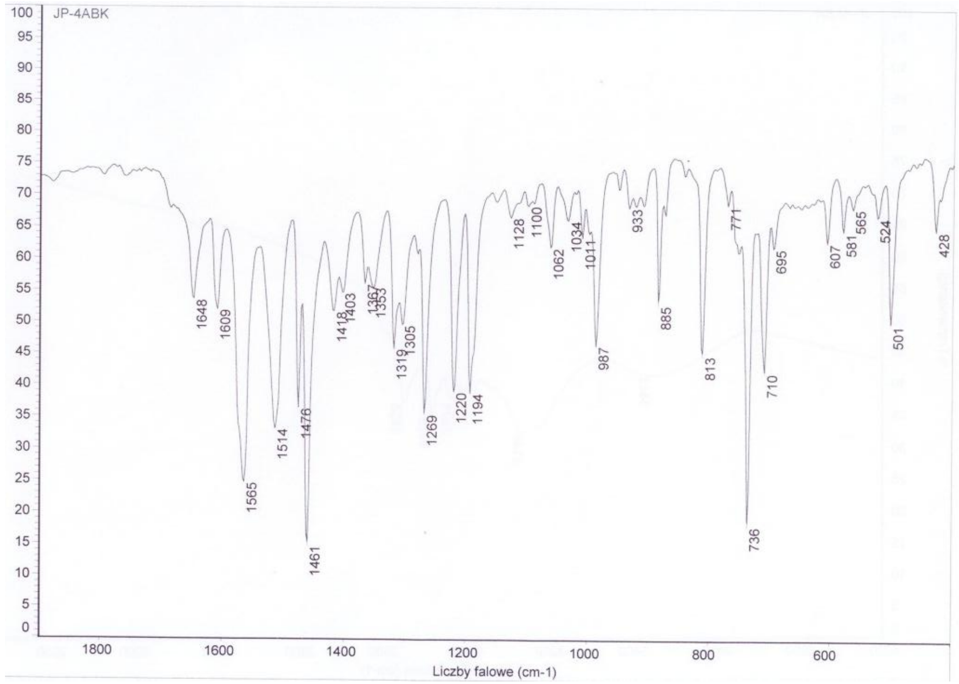

Figure S6. ^{1}H-NMR (400 MHz, DMSO-d_6) of (*Z*)-1-[1-(4,5-dihydro-1*H*-imidazol-2-yl)-2-(*p*-tolyl)vinyl]-1*H*-indole (**2b**).

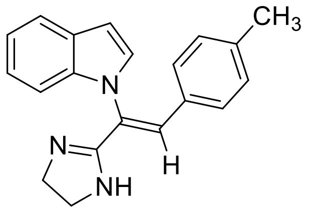

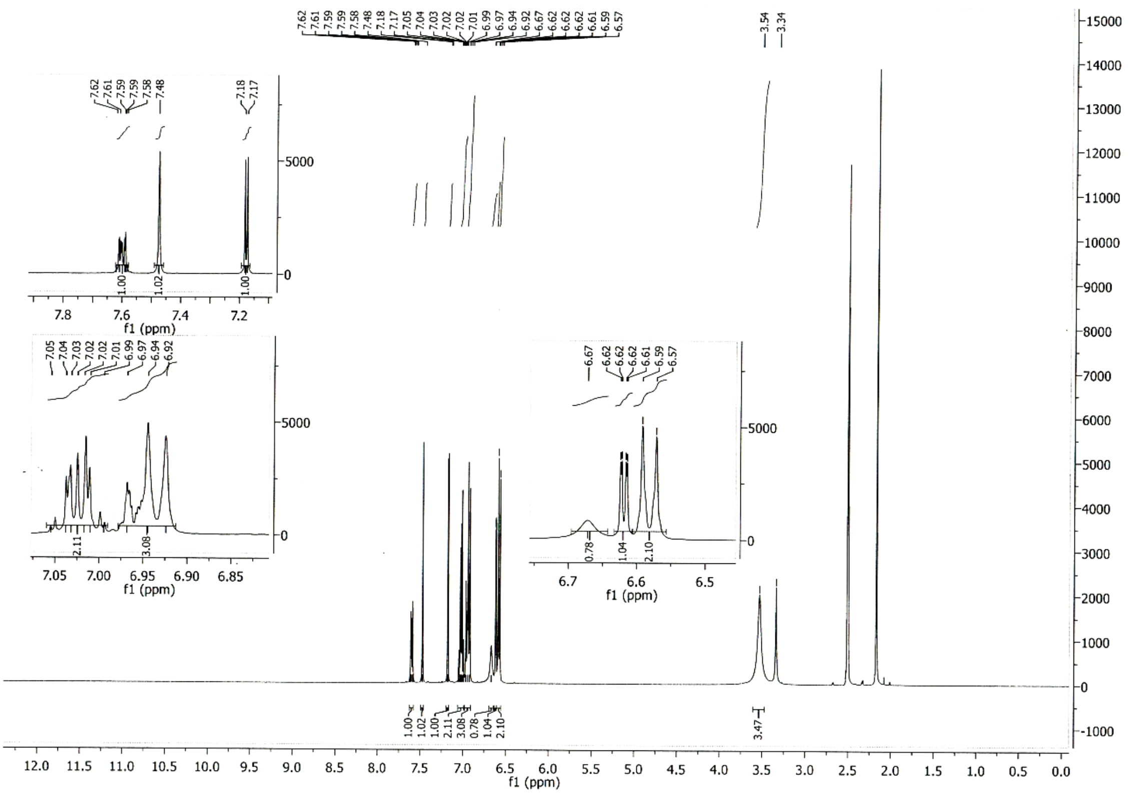

Figure S7. ^{13}C-NMR (100 MHz, DMSO-d_6) of (*Z*)-1-[1-(4,5-dihydro-1*H*-imidazol-2-yl)-2-(*p*-tolyl)vinyl]-1*H*-indole (**2b**).

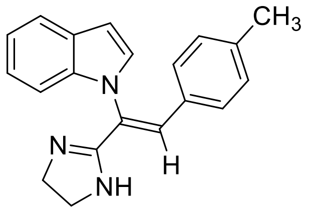

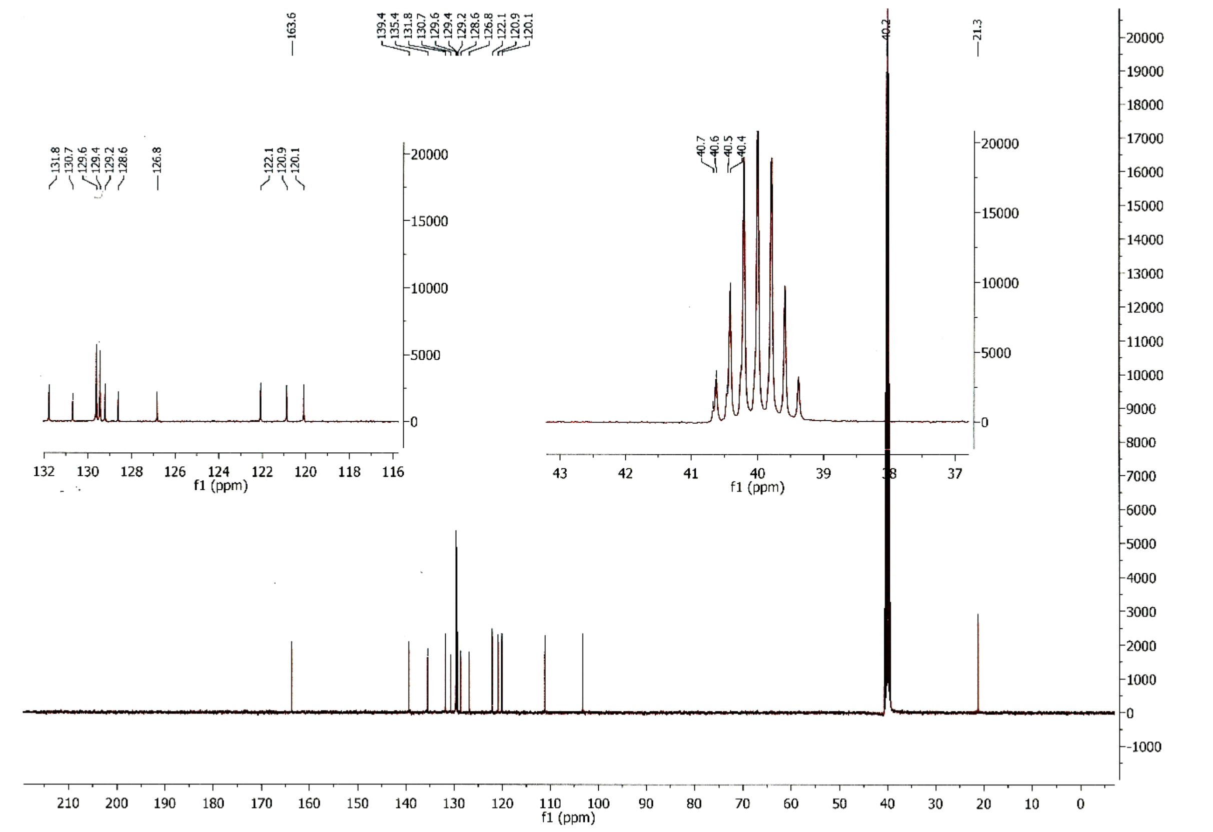

Figure S8. MS of (*Z*)-1-[1-(4,5-dihydro-1*H*-imidazol-2-yl)-2-(*p*-tolyl)vinyl]-1*H*-indole (**2b**).

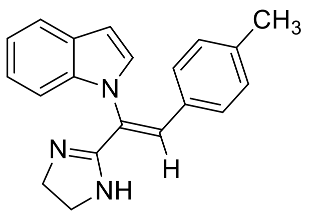

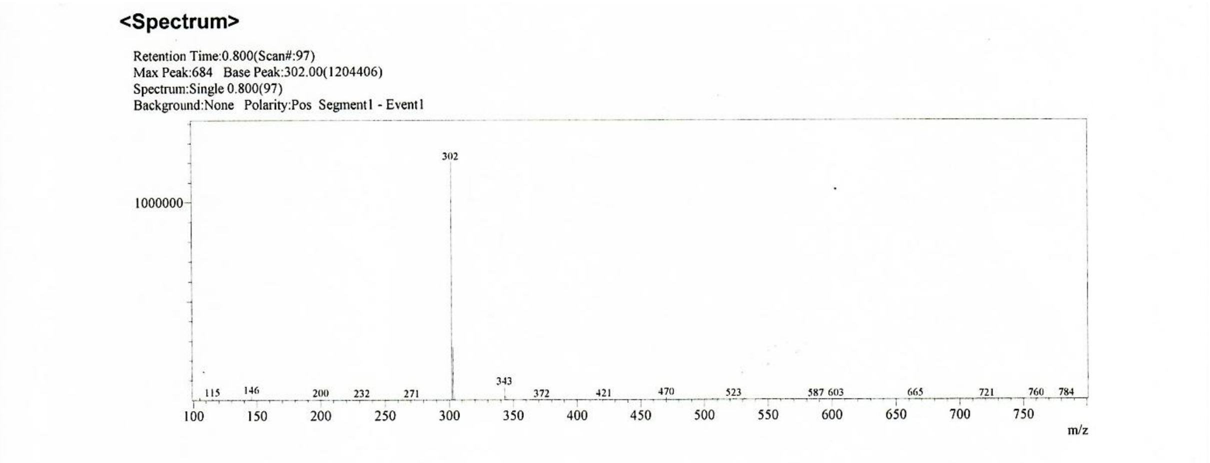

Figure S9. IR of (*Z*)-1-[1-(4,5-dihydro-1*H*-imidazol-2-yl)-2-(4-methoxyphenyl)vinyl]-1*H*-indole (**2c**).

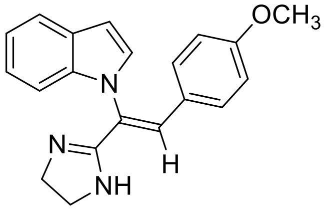

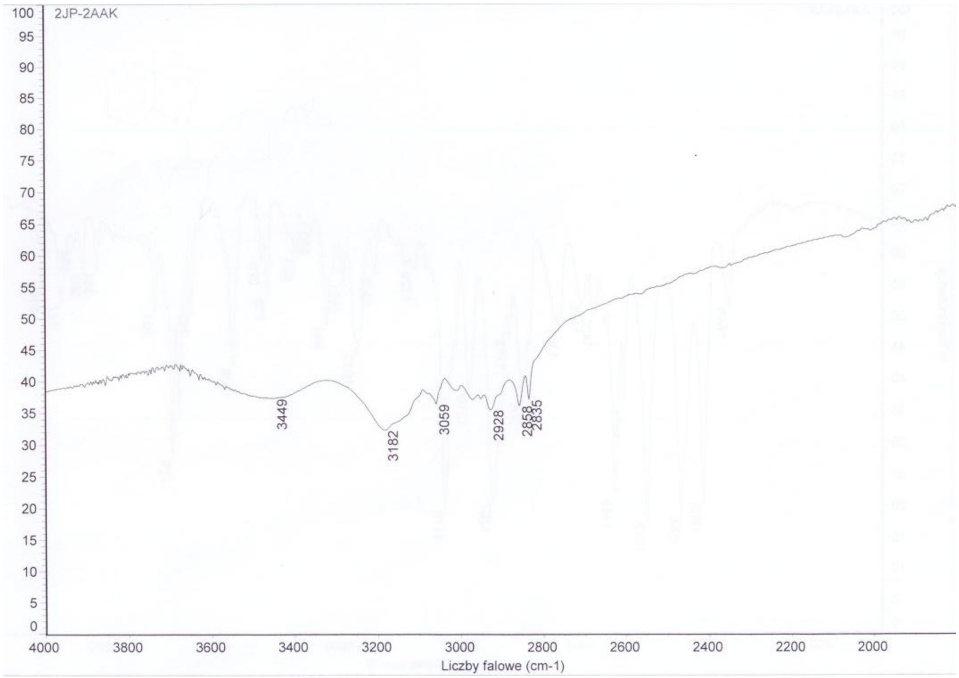

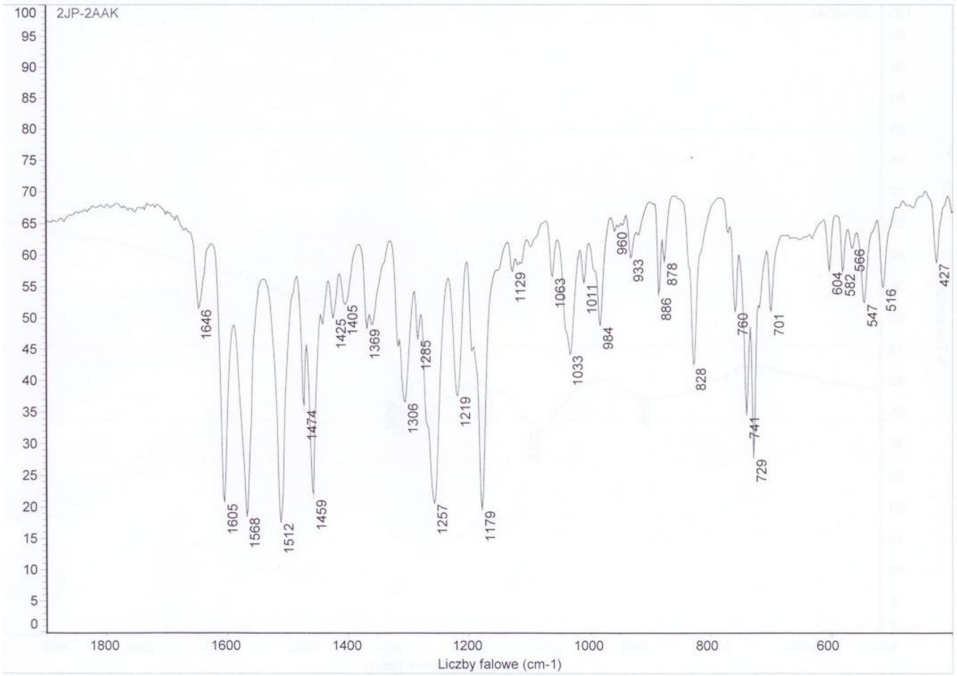

Figure S10. ^{1}H-NMR (400 MHz, DMSO-d_6) of (*Z*)-1-[1-(4,5-dihydro-1*H*-imidazol-2-yl)-2-(4-methoxyphenyl)vinyl]-1*H*-indole (**2c**).

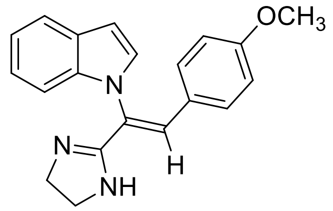

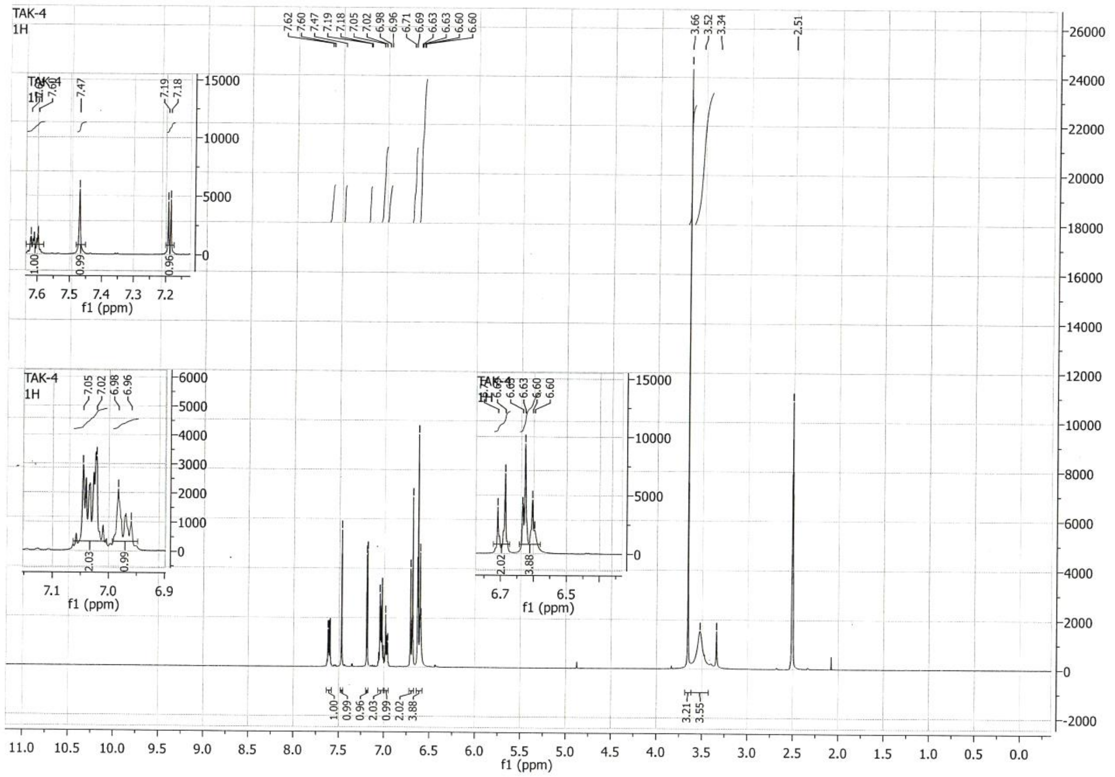

Figure S11. ^{13}C-NMR (100 MHz, DMSO-d_6) of (*Z*)-1-[1-(4,5-dihydro-1*H*-imidazol-2-yl)-2-(4-methoxyphenyl)vinyl]-1*H*-indole (**2c**).

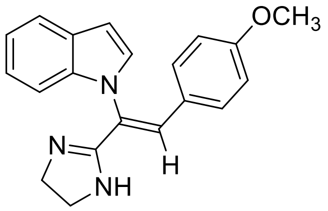

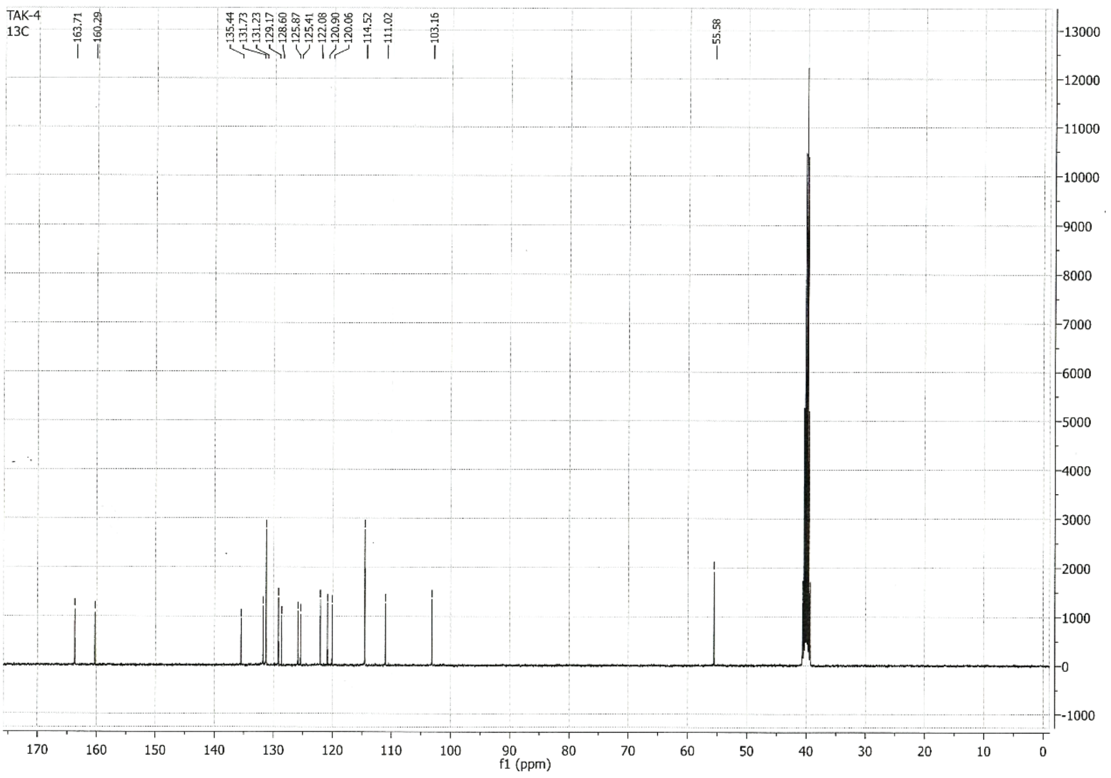

Figure S12. MS of (*Z*)-1-[1-(4,5-dihydro-1*H*-imidazol-2-yl)-2-(4-methoxyphenyl)vinyl]-1*H*-indole (**2c**).

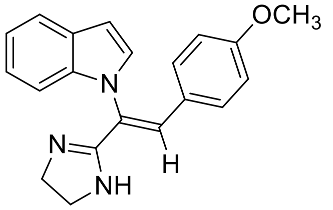

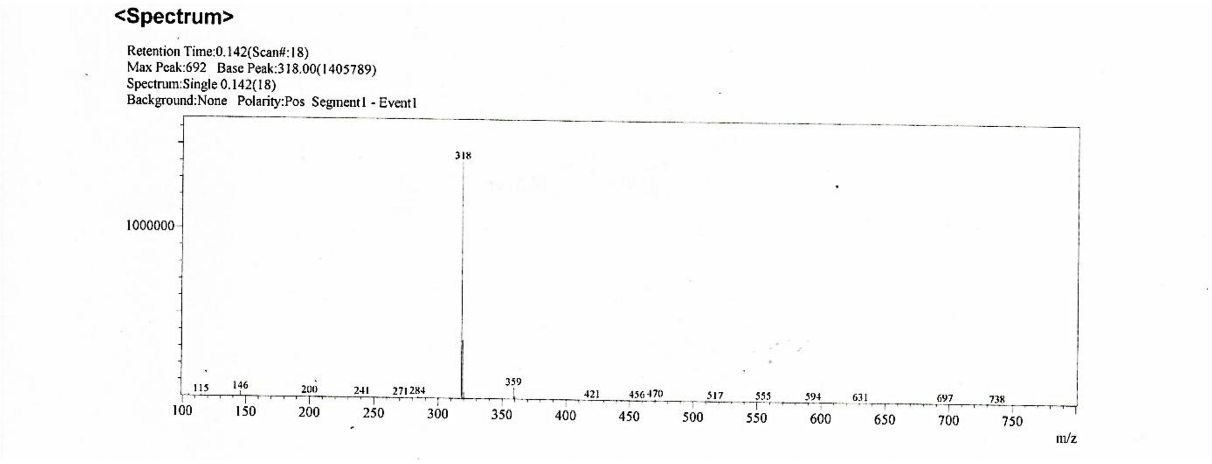

Figure S13. IR of (*Z*)-1-[1-(4,5-dihydro-1*H*-imidazol-2-yl)-2-(4-fluorophenyl)vinyl]-1*H*-indole (**2d**).

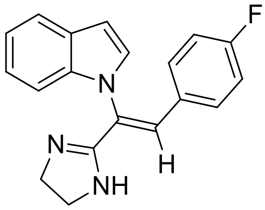

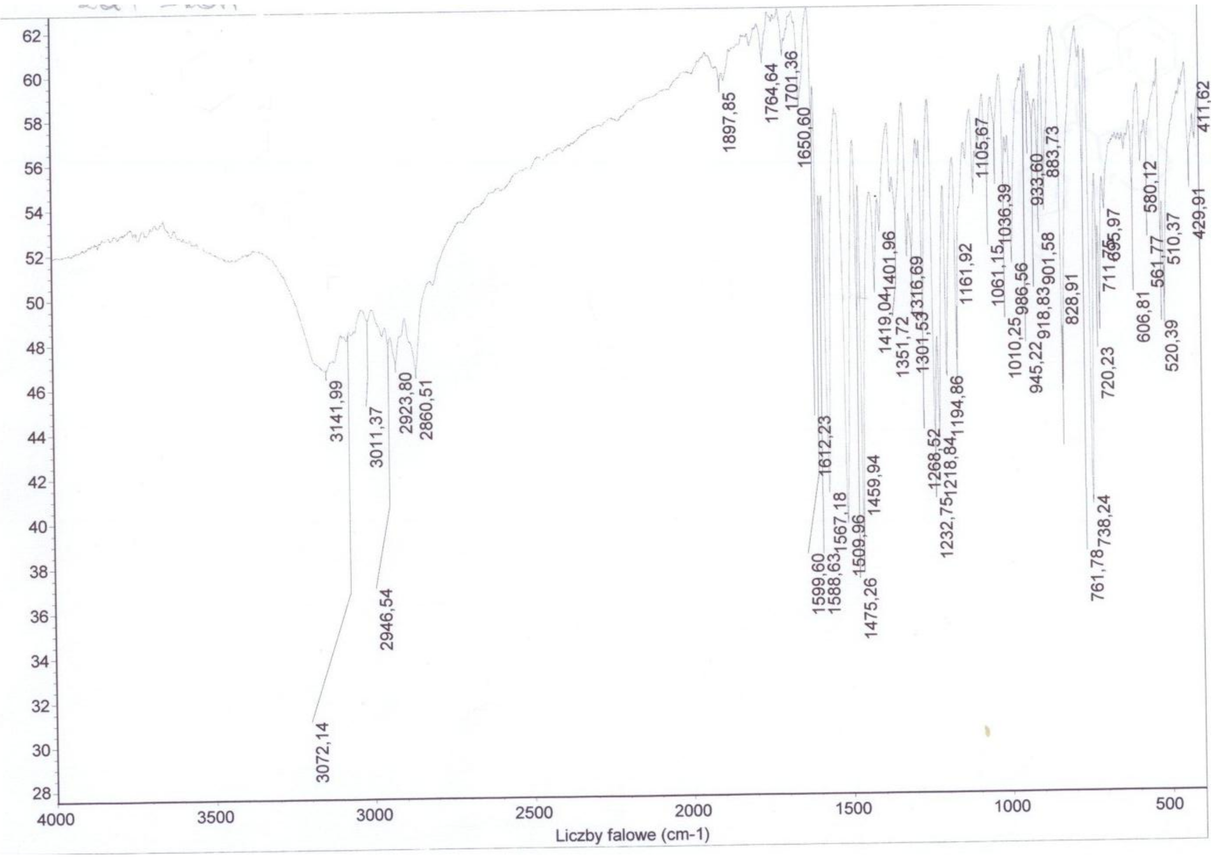

Figure S14. ^{1}H-NMR (400 MHz, DMSO-d_6) of (*Z*)-1-[1-(4,5-dihydro-1*H*-imidazol-2-yl)-2-(4-fluorophenyl)vinyl]-1*H*-indole (**2d**).

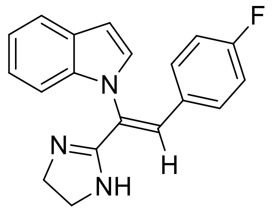

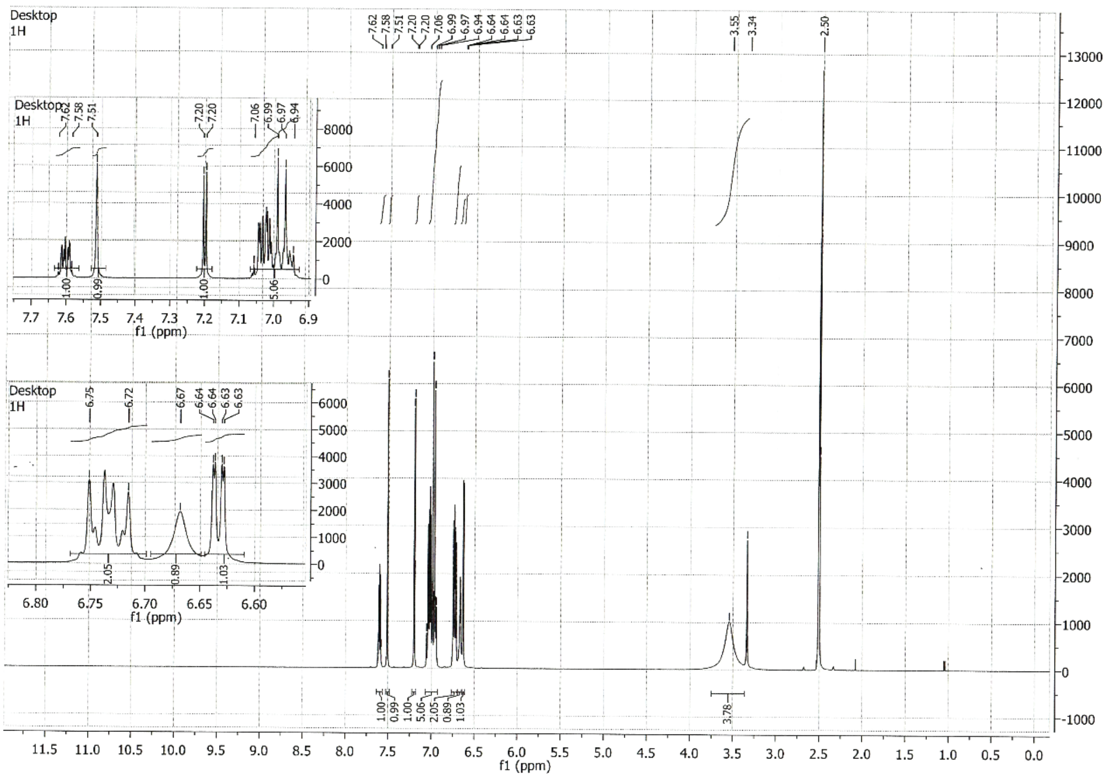

Figure S15. ^{13}C-NMR (100 MHz, DMSO-d_6) of (Z)-1-[1-(4,5-dihydro-1H-imidazol-2-yl)-2-(4-fluorophenyl)vinyl]-1H-indole (**2d**).

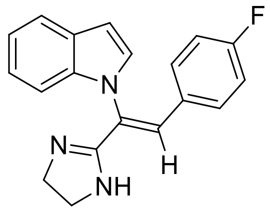

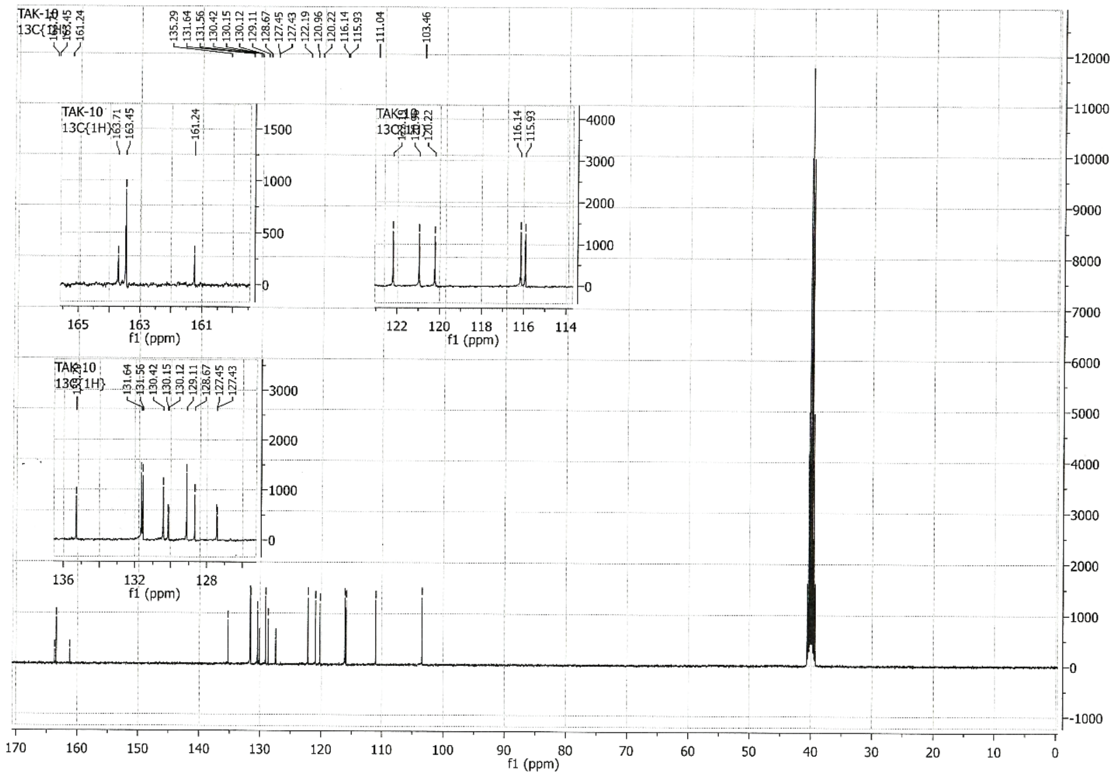

Figure S16. MS of (*Z*)-1-[1-(4,5-dihydro-1*H*-imidazol-2-yl)-2-(4-fluorophenyl)vinyl]-1*H*-indole (**2d**).

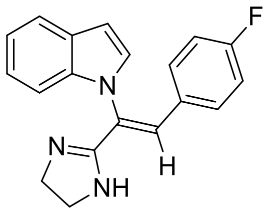

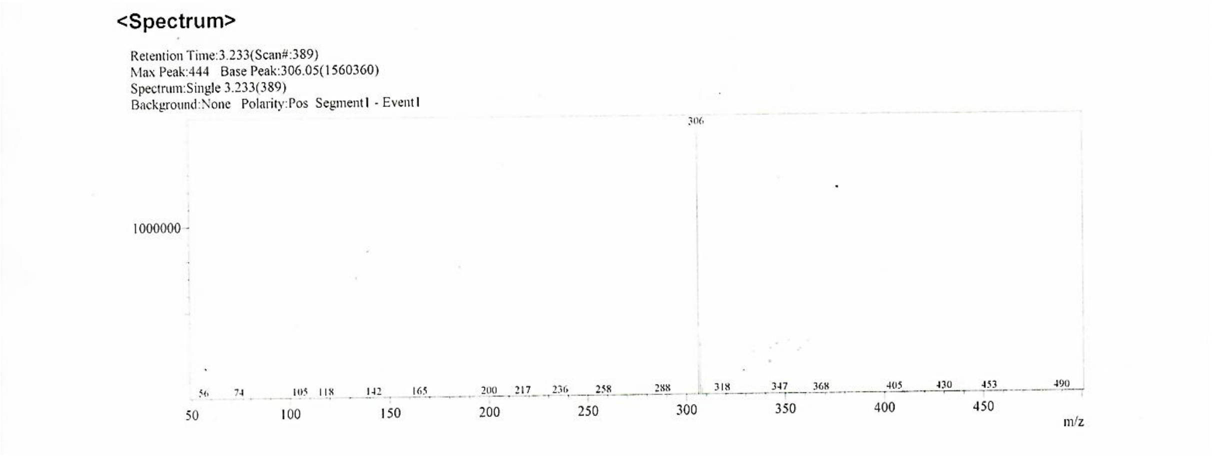

Figure S17. IR of (*Z*)-1-[2-(4-chlorophenyl)-1-(4,5-dihydro-1*H*-imidazol-2-yl)vinyl]-1*H*-indole (**2e**).

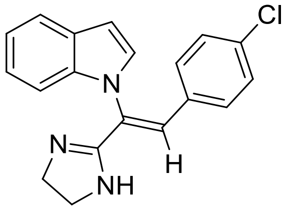

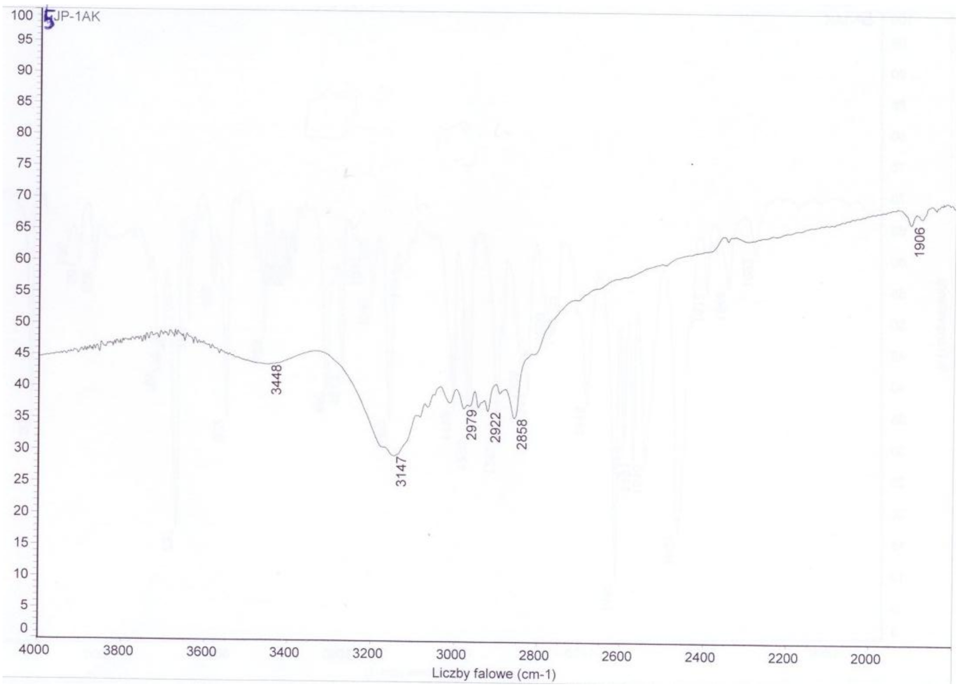

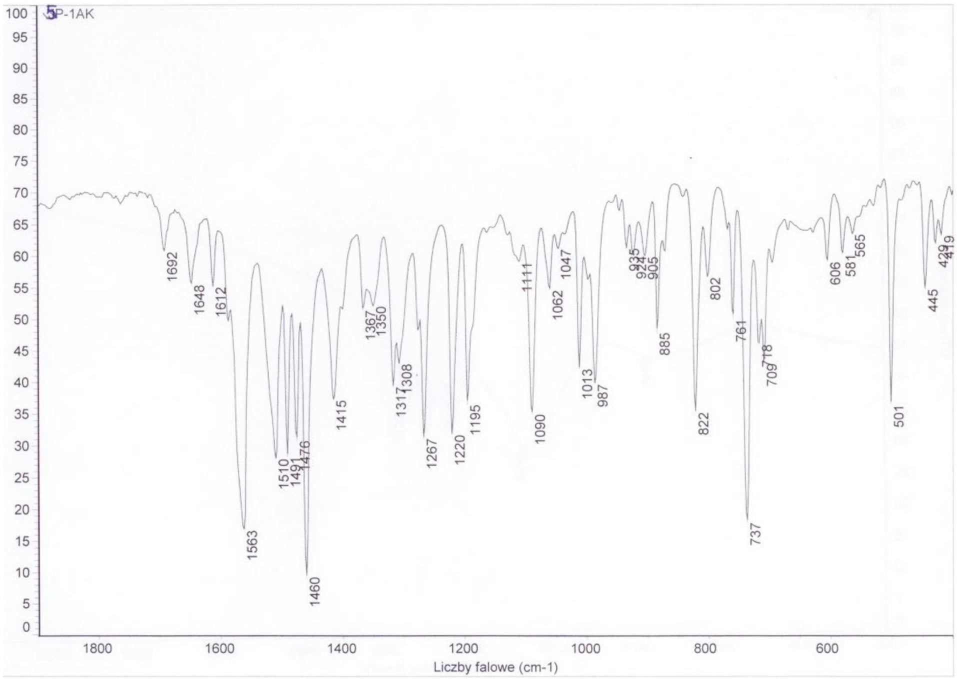

Figure S18. ^{1}H-NMR (400 MHz, DMSO-d_6) of (*Z*)-1-[2-(4-chlorophenyl)-1-(4,5-dihydro-1*H*-imidazol-2-yl)vinyl]-1*H*-indole (**2e**).

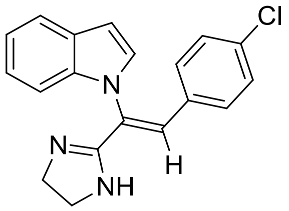

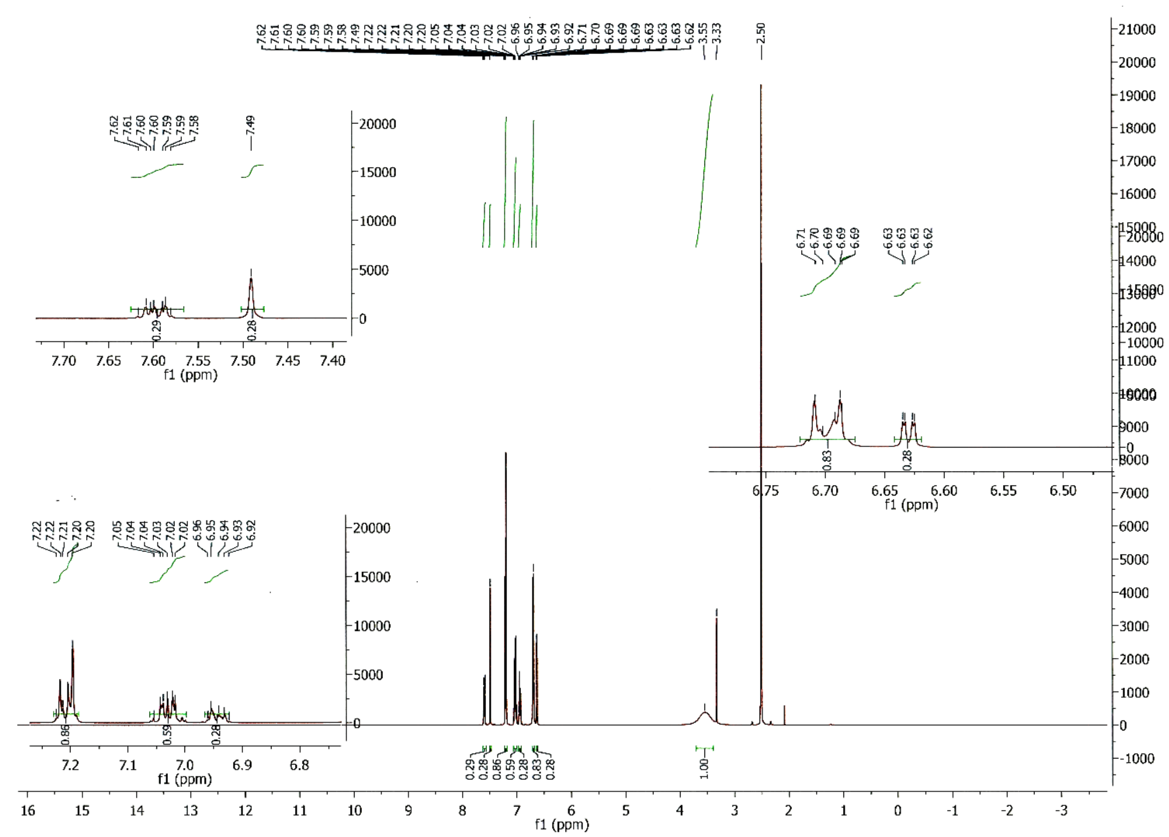

Figure S19. ^{13}C-NMR (100 MHz, DMSO-d_6) of (*Z*)-1-[2-(4-chlorophenyl)-1-(4,5-dihydro-1*H*-imidazol-2-yl)vinyl]-1*H*-indole (**2e**).

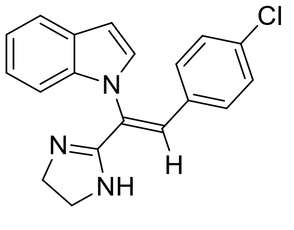

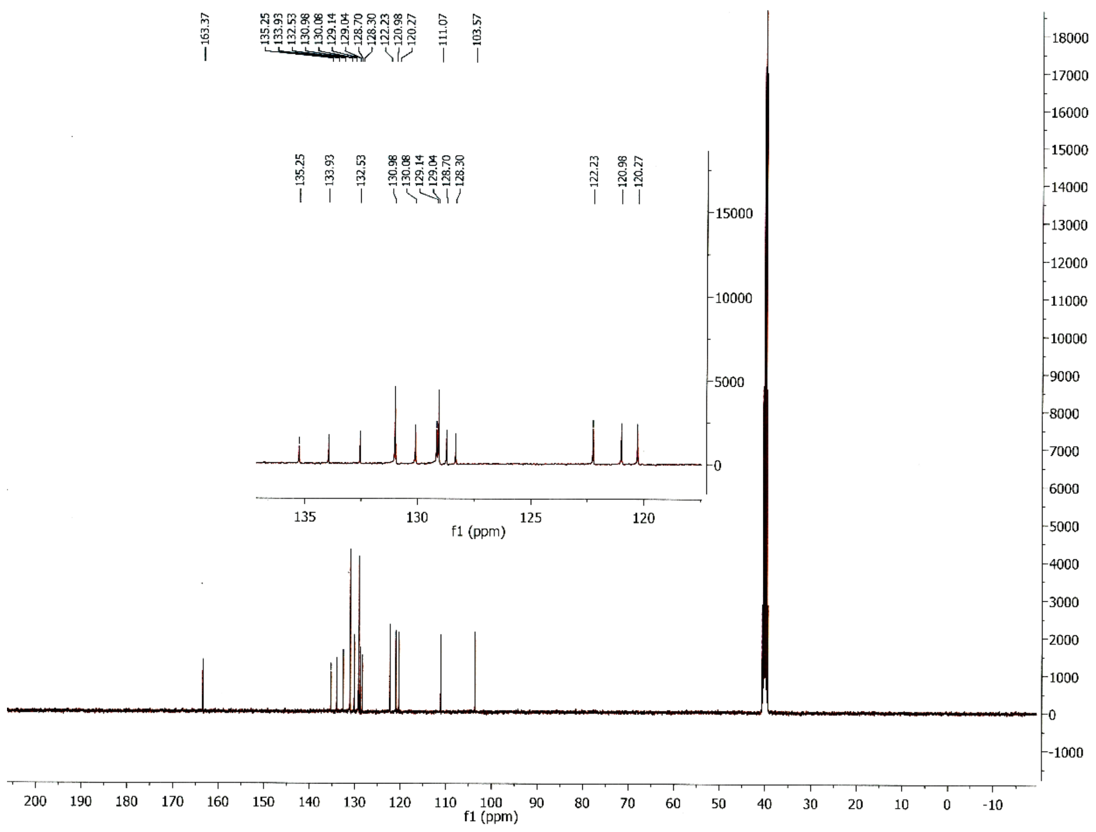

Figure S20. MS of (*Z*)-1-[2-(4-chlorophenyl)-1-(4,5-dihydro-1*H*-imidazol-2-yl)vinyl]-1*H*-indole (**2e**).

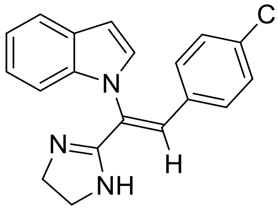

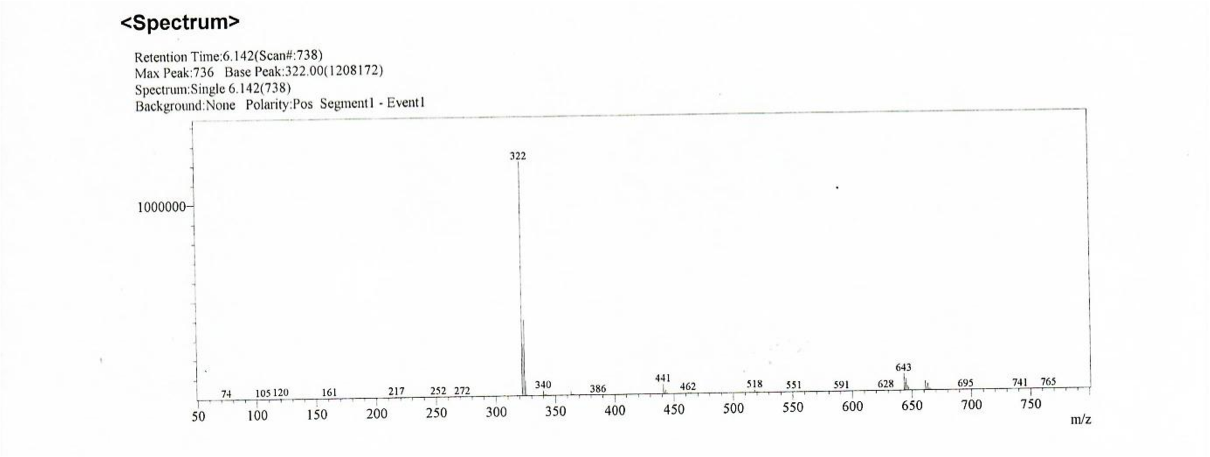

Figure S21. IR of (*Z*)-1-[2-(4-bromophenyl)-1-(4,5-dihydro-1*H*-imidazol-2-yl)vinyl]-1*H*-indole (**2f**).

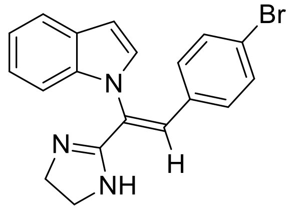

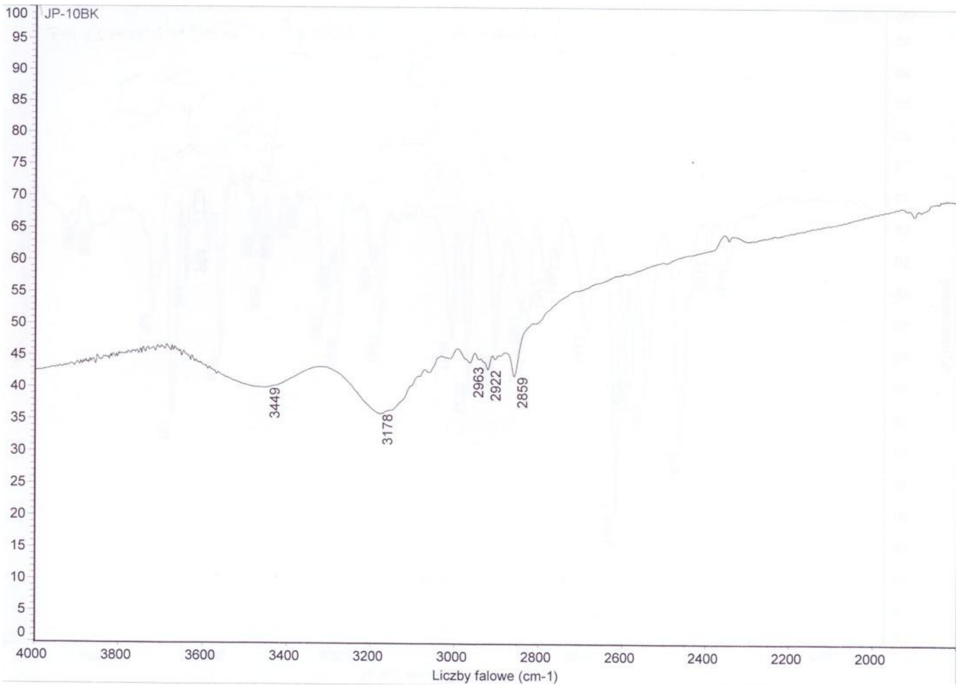

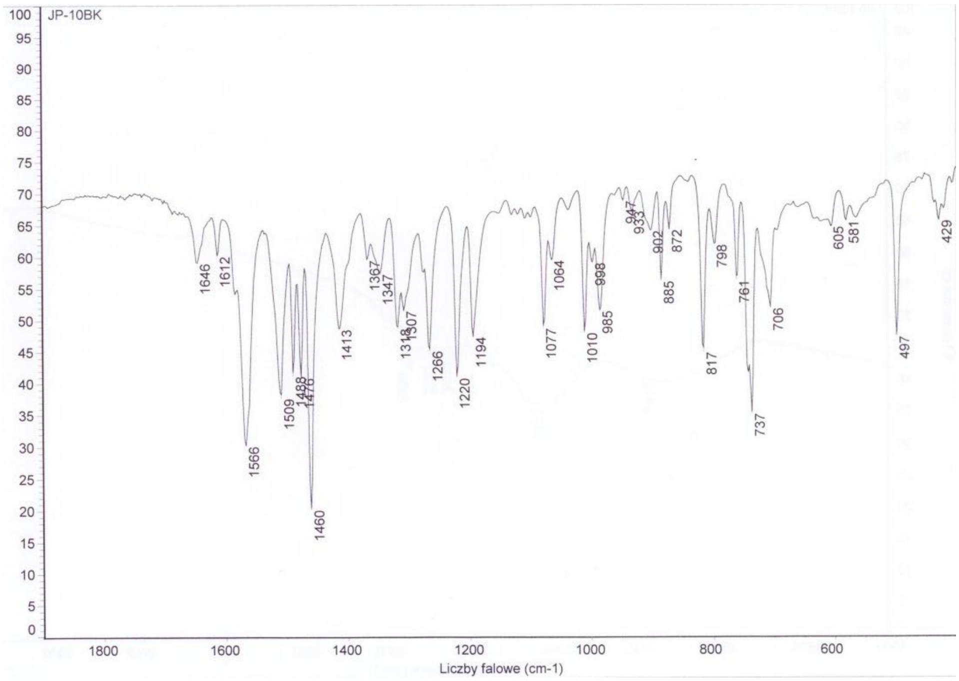

Figure S22. ^{1}H-NMR (400 MHz, DMSO-d_6) of (*Z*)-1-[2-(4-bromophenyl)-1-(4,5-dihydro-1*H*-imidazol-2-yl)vinyl]-1*H*-indole (**2f**).

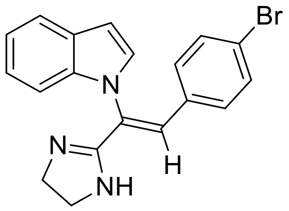

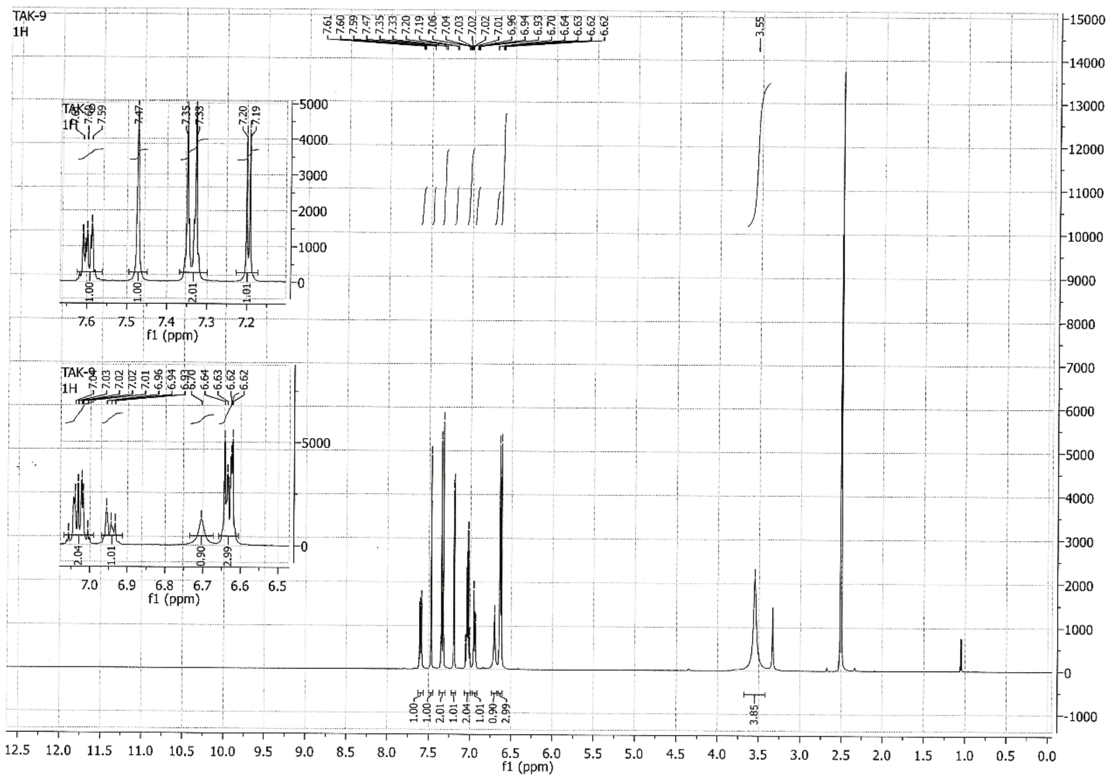

Figure S23. ^{13}C-NMR (100 MHz, DMSO-d_6) of (*Z*)-1-[2-(4-bromophenyl)-1-(4,5-dihydro-1*H*-imidazol-2-yl)vinyl]-1*H*-indole (**2f**).

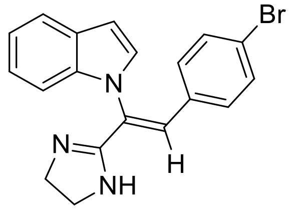

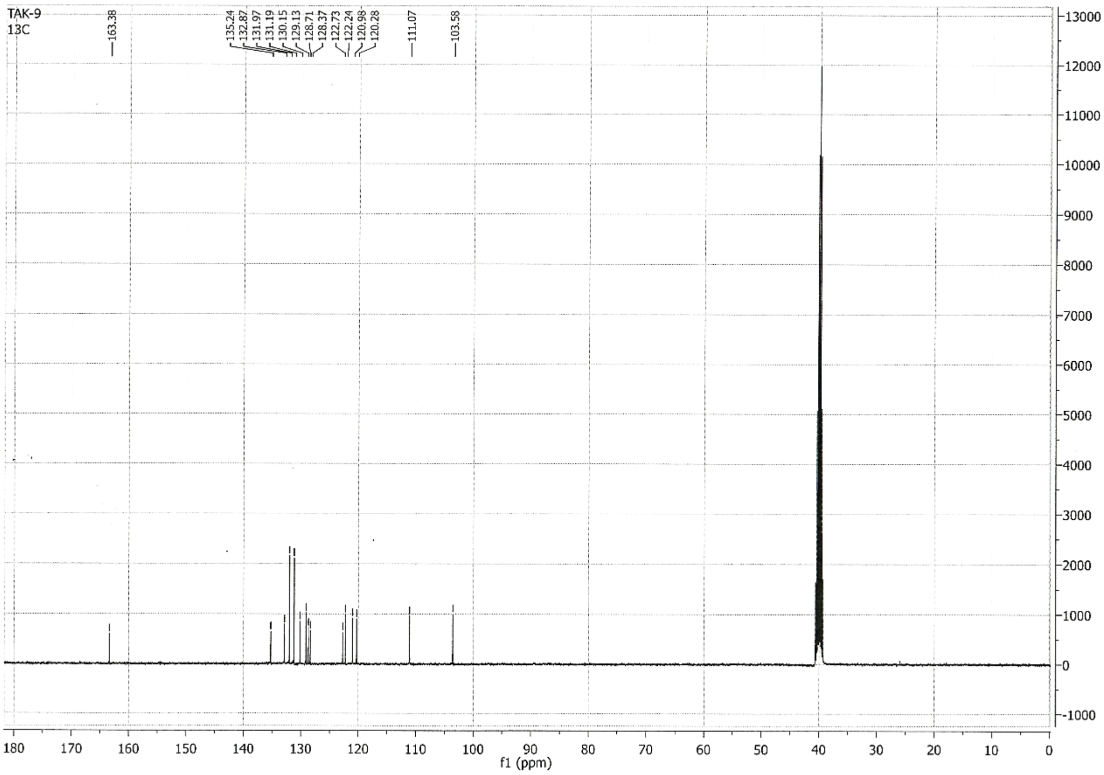

Figure S24. MS of (*Z*)-1-[2-(4-bromophenyl)-1-(4,5-dihydro-1*H*-imidazol-2-yl)vinyl]-1*H*-indole (**2f**).

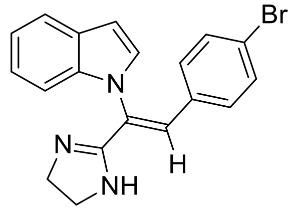

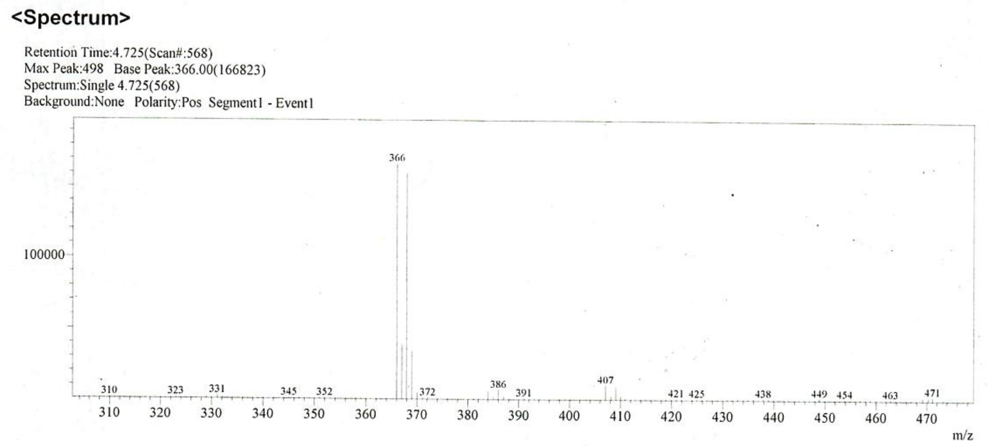

Figure S25. IR of (*Z*)-1-[1-(4,5-dihydro-1*H*-imidazol-2-yl)-2-(4-nitrophenyl)vinyl]-1*H*-indole (**2g**).

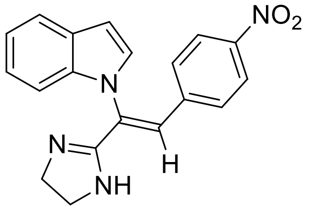

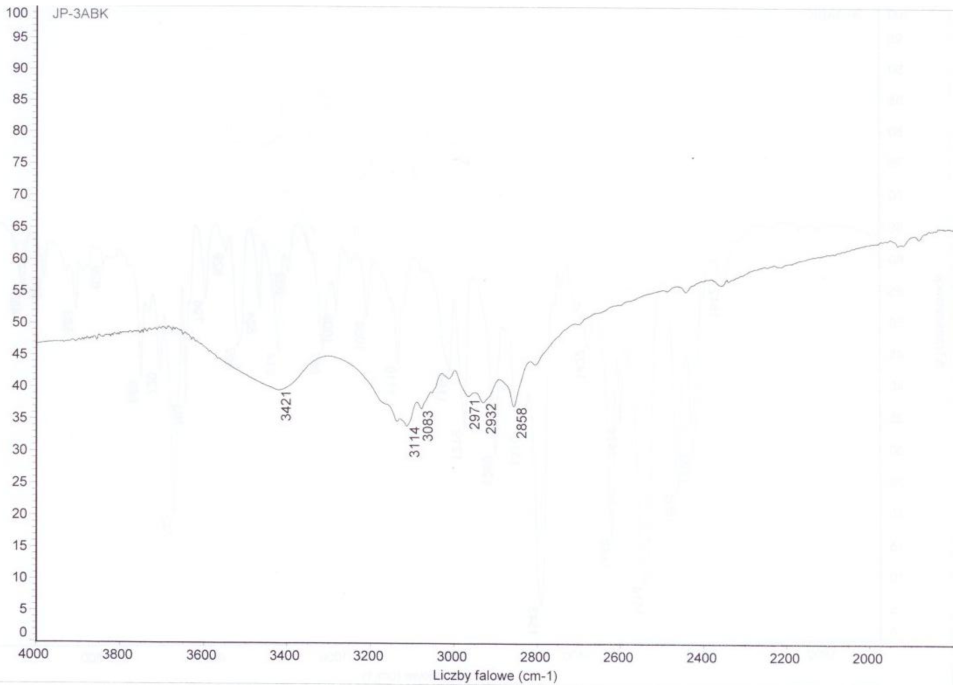

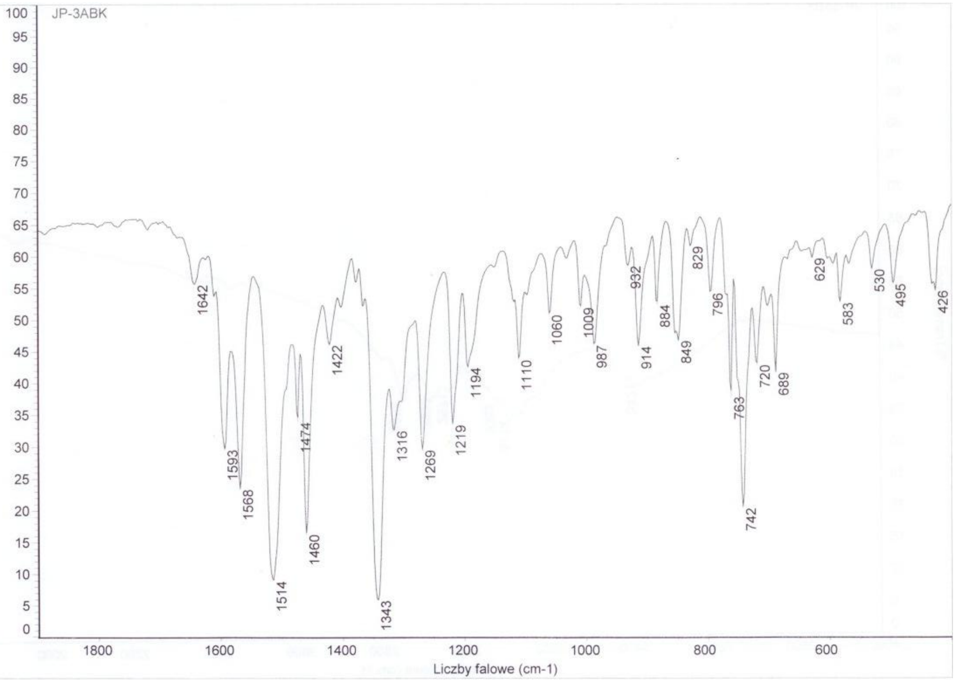

Figure S26. ^{1}H-NMR (400 MHz, DMSO-d_6) of (*Z*)-1-[1-(4,5-dihydro-1*H*-imidazol-2-yl)-2-(4-nitrophenyl)vinyl]-1*H*-indole (**2g**).

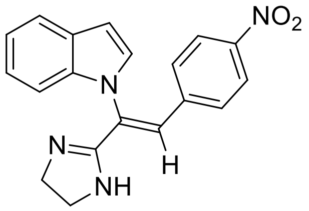

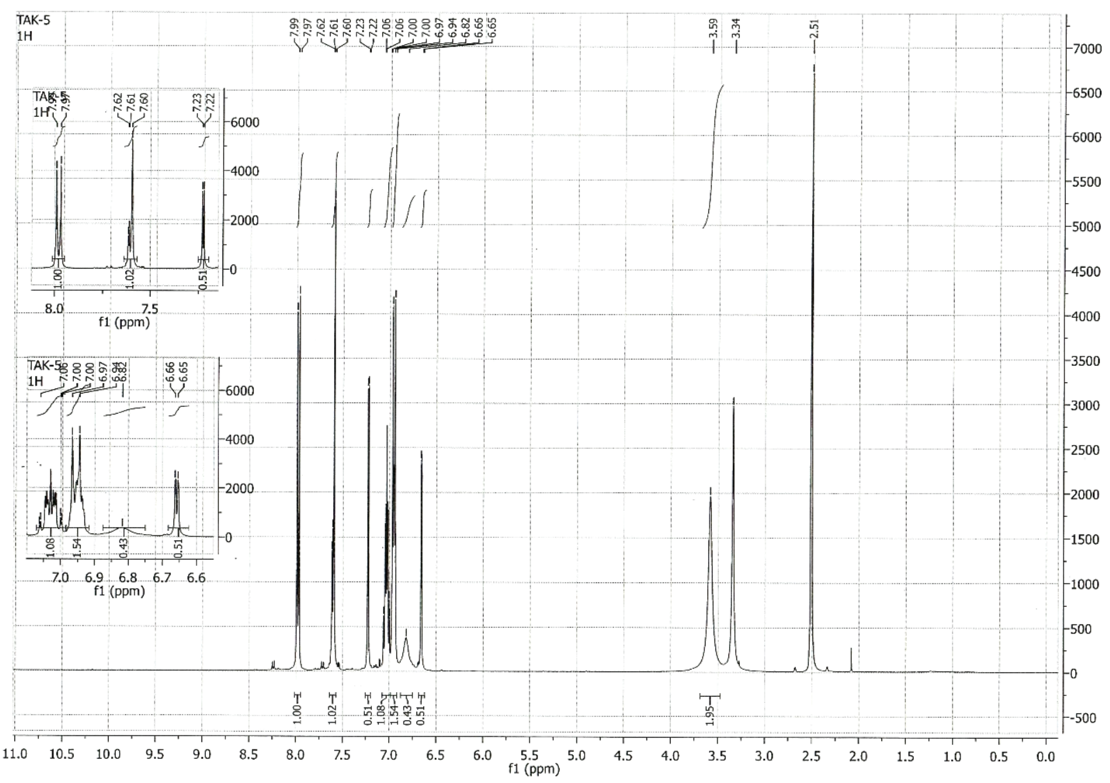

Figure S27. ^{13}C-NMR (100 MHz, DMSO-d_6) of (*Z*)-1-[1-(4,5-dihydro-1*H*-imidazol-2-yl)-2-(4-nitrophenyl)vinyl]-1*H*-indole (**2g**).

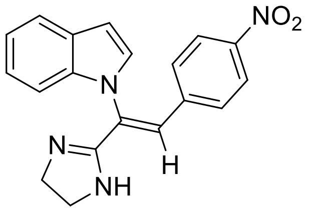

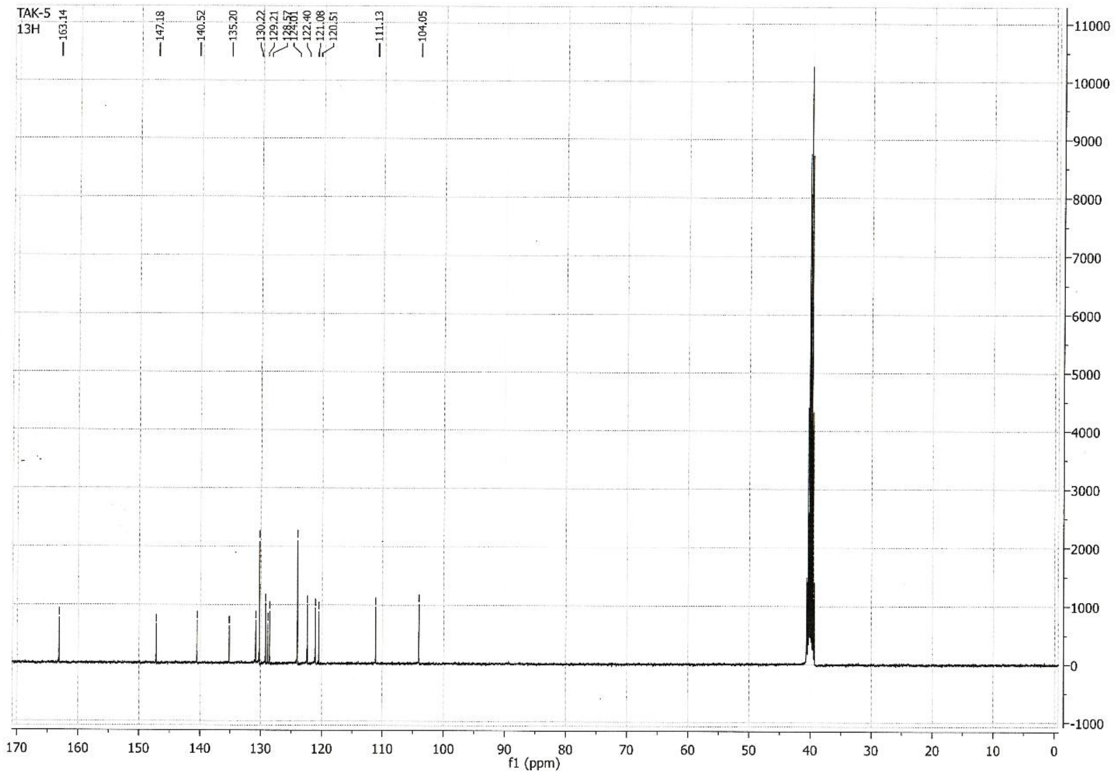

Figure S28. ^{1}H-NMR (500 MHz, DMSO-d_6 + TFA) of (*Z*)-1-[1-(4,5-dihydro-1*H*-imidazol-2-yl)-2-(4-nitrophenyl)vinyl]-1*H*-indole (**2g/A**).

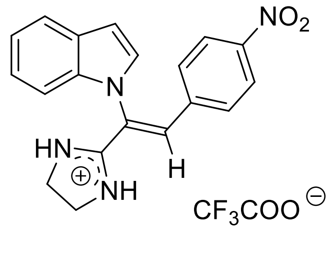

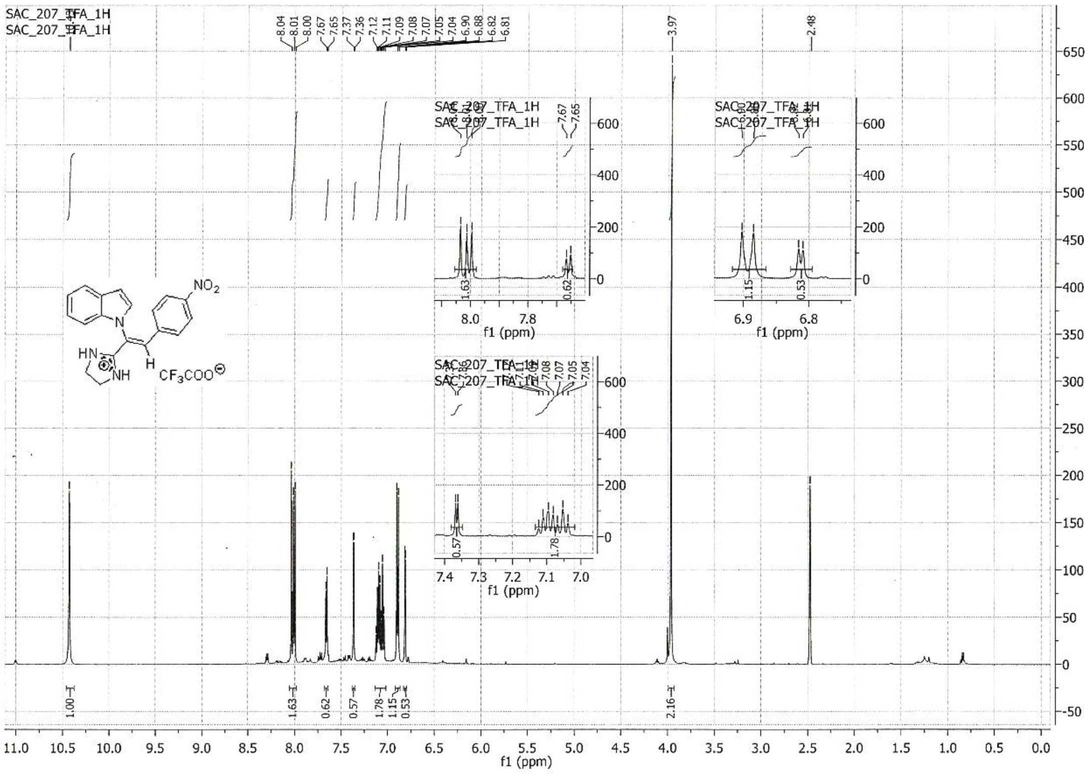

Figure S29. ^{13}C-NMR (125 MHz, DMSO-d_6 + TFA) of (*Z*)-1-[1-(4,5-dihydro-1*H*-imidazol-2-yl)-2-(4-nitrophenyl)vinyl]-1*H*-indole (**2g/A**).

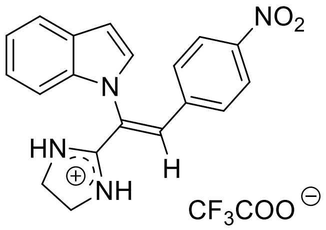

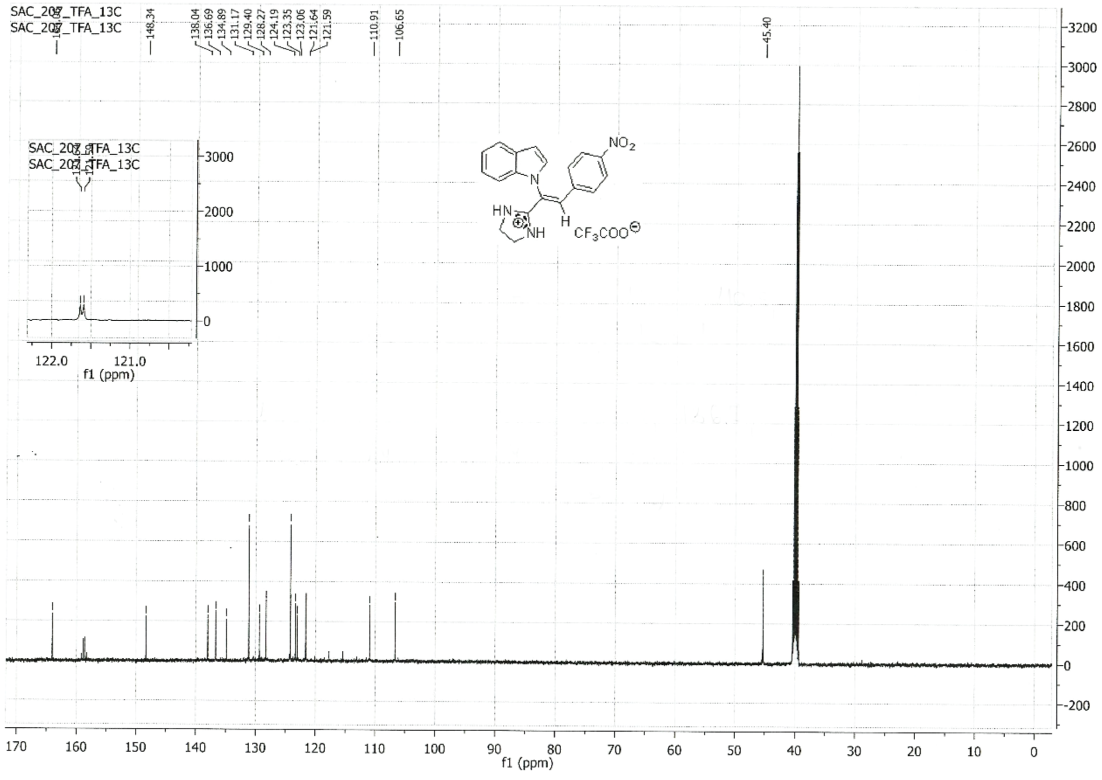

Figure S30. HSQC (DMSO-d_6 + TFA) of (*Z*)-1-[1-(4,5-dihydro-1*H*-imidazol-2-yl)-2-(4-nitrophenyl)vinyl]-1*H*-indole (**2g/A**).

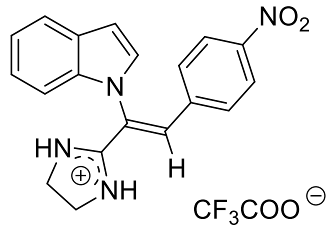

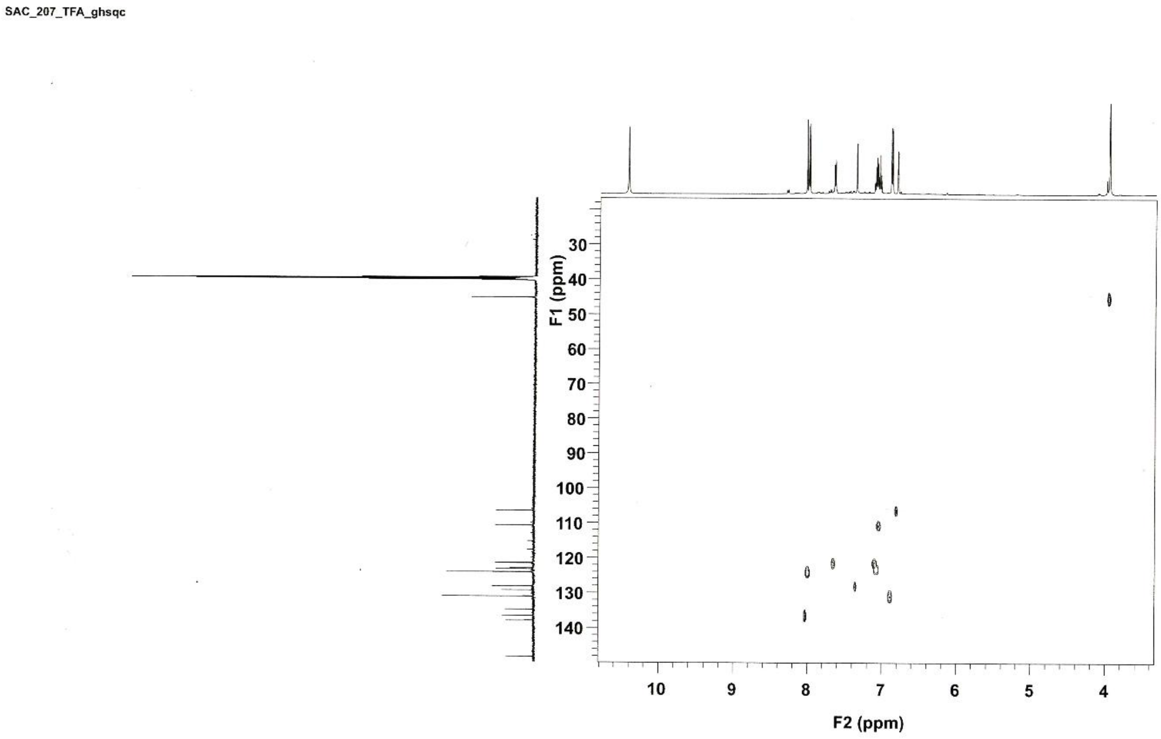

Figure S31. HMBC (DMSO-d_6 + TFA) of (*Z*)-1-[1-(4,5-dihydro-1*H*-imidazol-2-yl)-2-(4-nitrophenyl)vinyl]-1*H*-indole (**2g/A**).

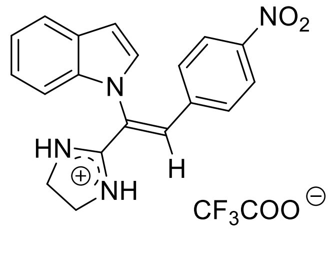

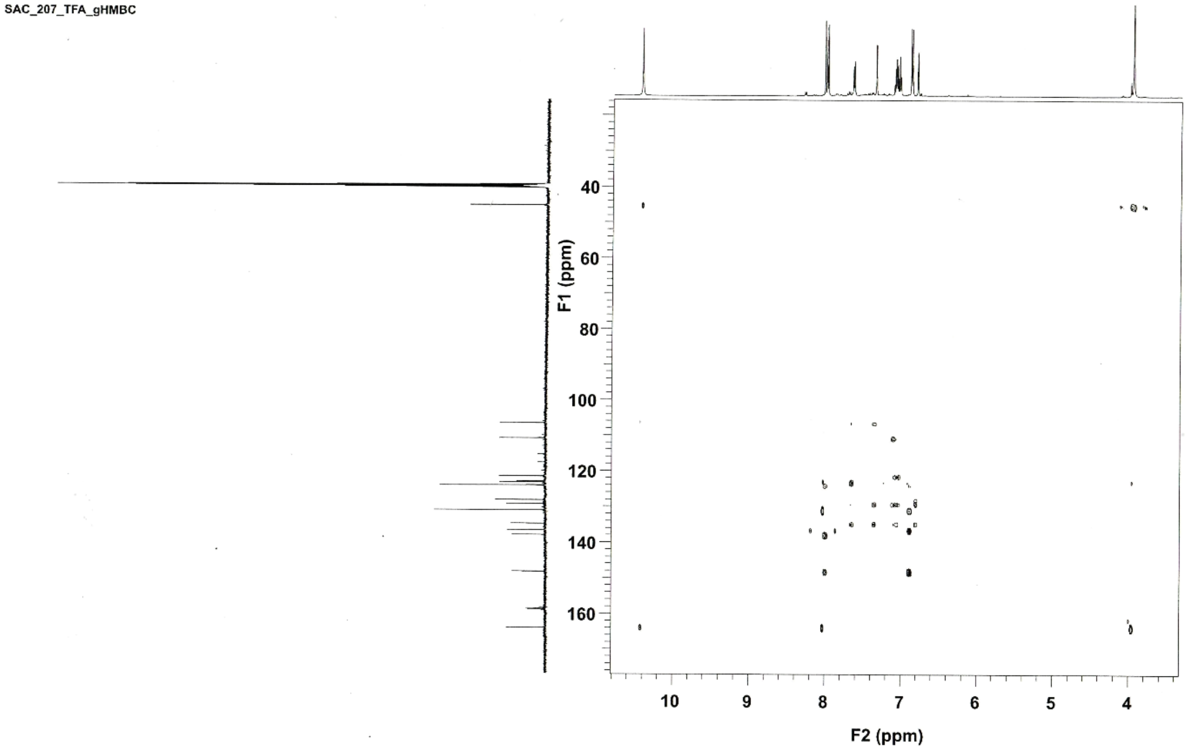

Figure S32. MS of (*Z*)-1-[1-(4,5-dihydro-1*H*-imidazol-2-yl)-2-(4-nitrophenyl)vinyl]-1*H*-indole (**2g**).

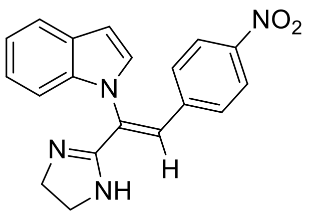

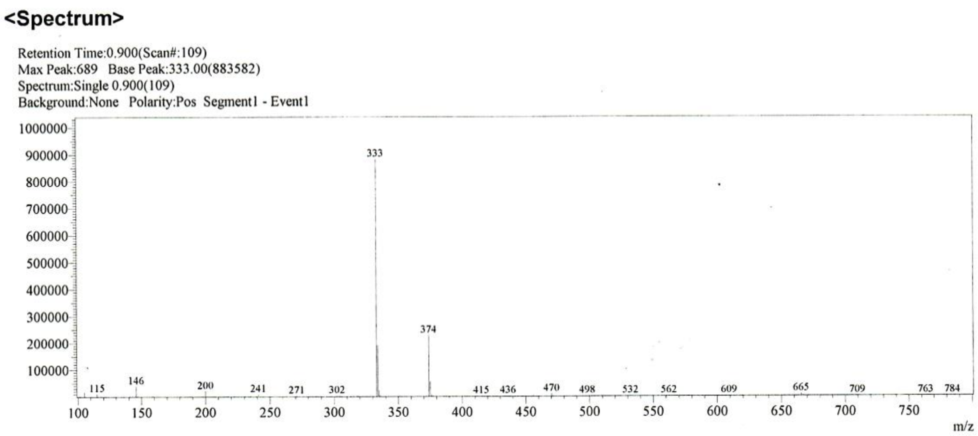

Figure S33. IR of (*Z*)-1-[1-(4,5-dihydro-1*H*-imidazol-2-yl)-2-(naphthalen-1-yl)vinyl]-1*H*-indole (**2h**).

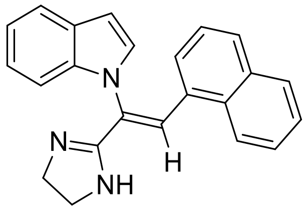

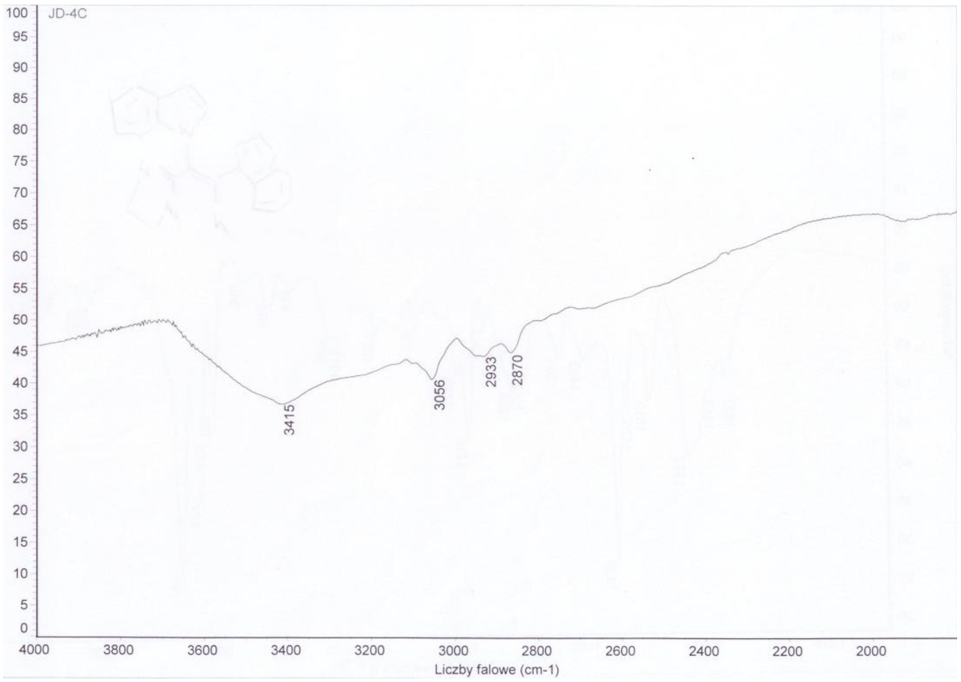

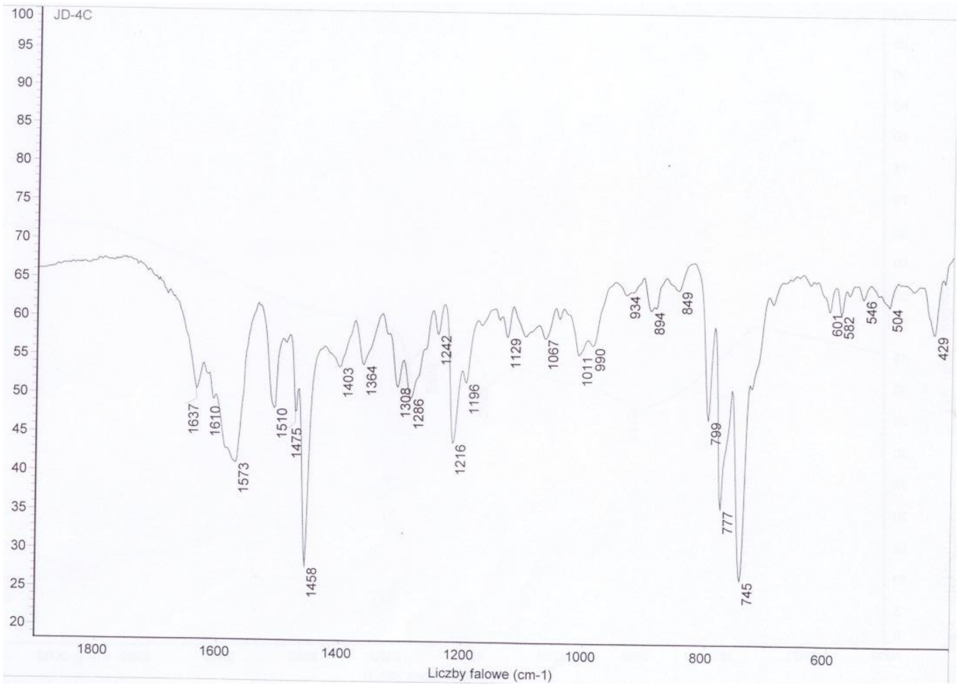

Figure S34. ^{1}H-NMR (400 MHz, DMSO-d_6) of (*Z*)-1-[1-(4,5-dihydro-1*H*-imidazol-2-yl)-2-(naphthalen-1-yl)vinyl]-1*H*-indole (**2h**).

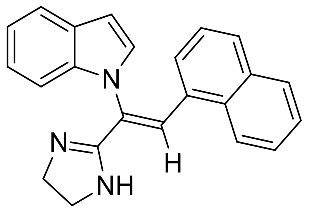

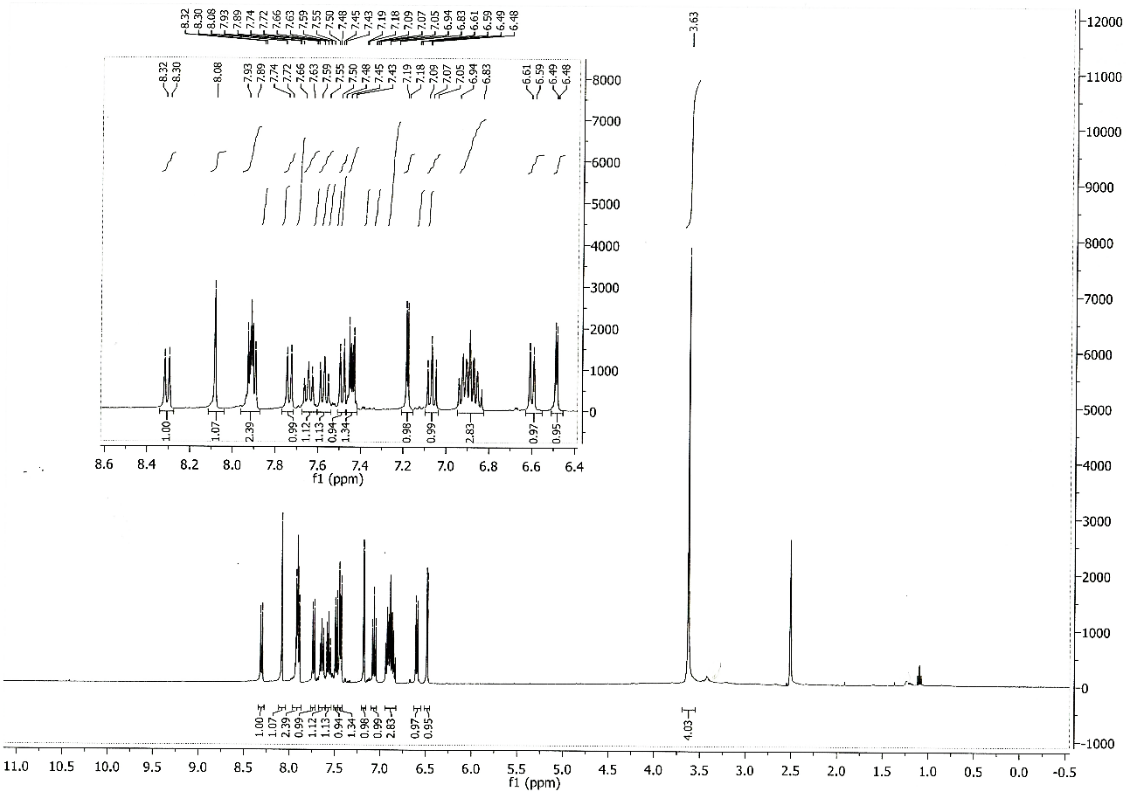

Figure S35. ^{13}C-NMR (100 MHz, DMSO-d_6) of (*Z*)-1-[1-(4,5-dihydro-1*H*-imidazol-2-yl)-2-(naphthalen-1-yl)vinyl]-1*H*-indole (**2h**).

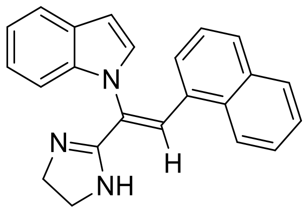

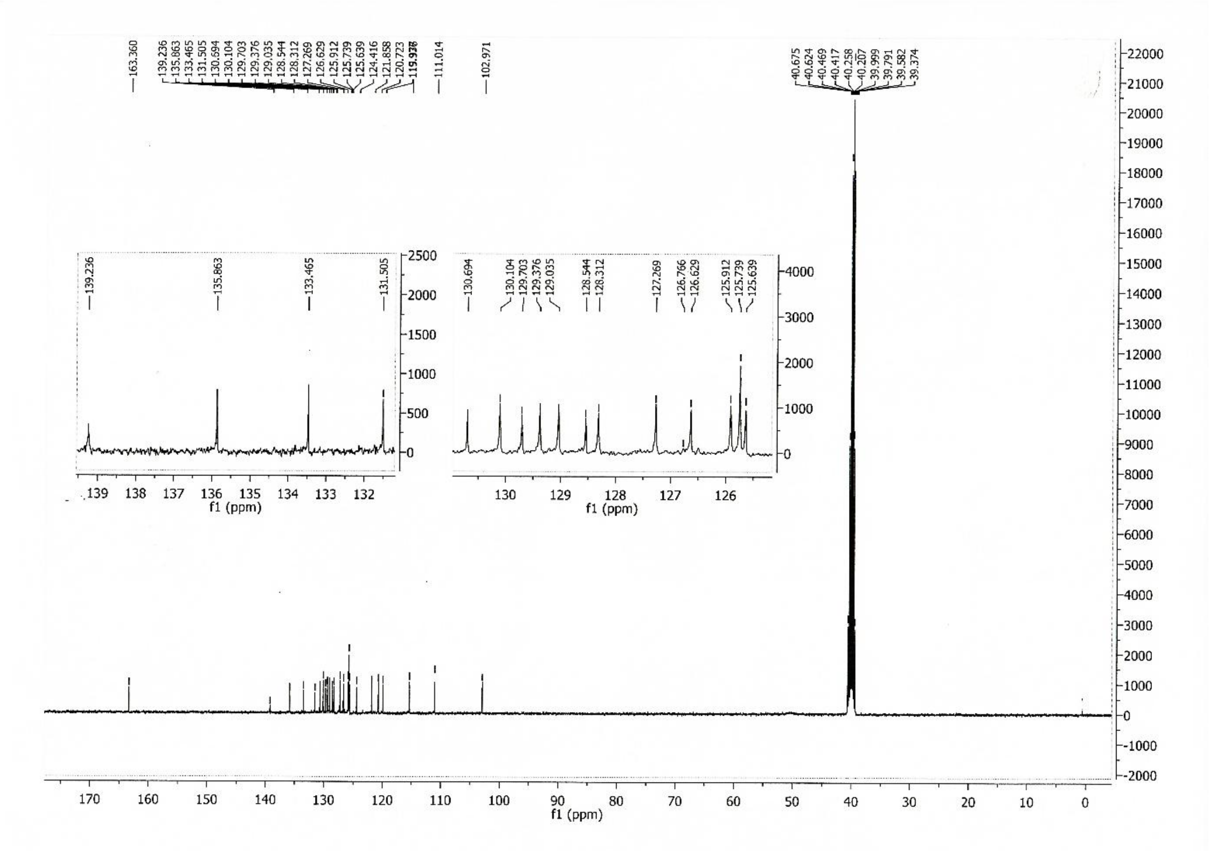

Figure S36. MS of (*Z*)-1-[1-(4,5-dihydro-1*H*-imidazol-2-yl)-2-(naphthalen-1-yl)vinyl]-1*H*-indole (**2h**).

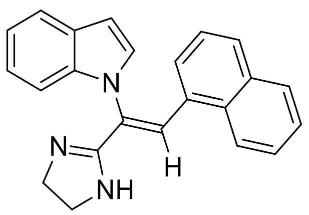

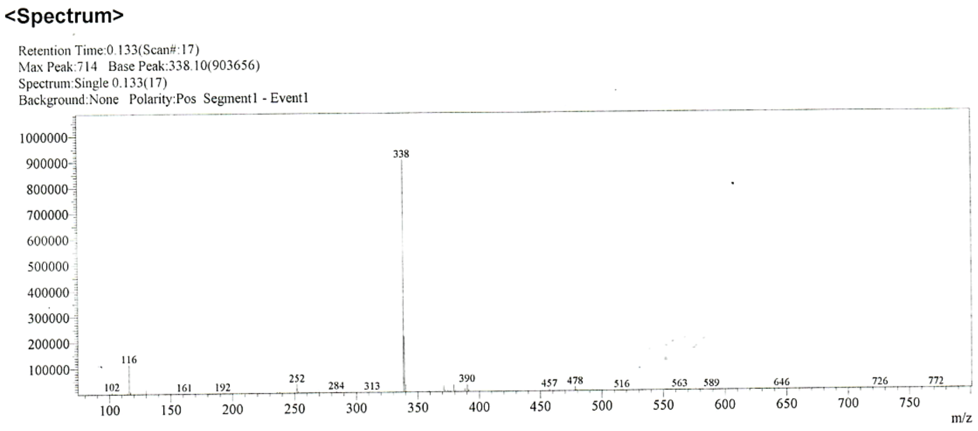

Figure S37. IR of (*Z*)-1-[1-(4,5-dihydro-1*H*-imidazol-2-yl)-2-(naphthalen-2-yl)vinyl]-1*H*-indole (**2i**).

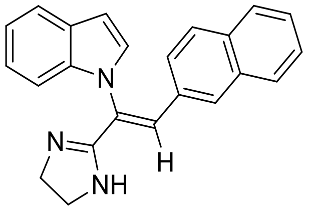

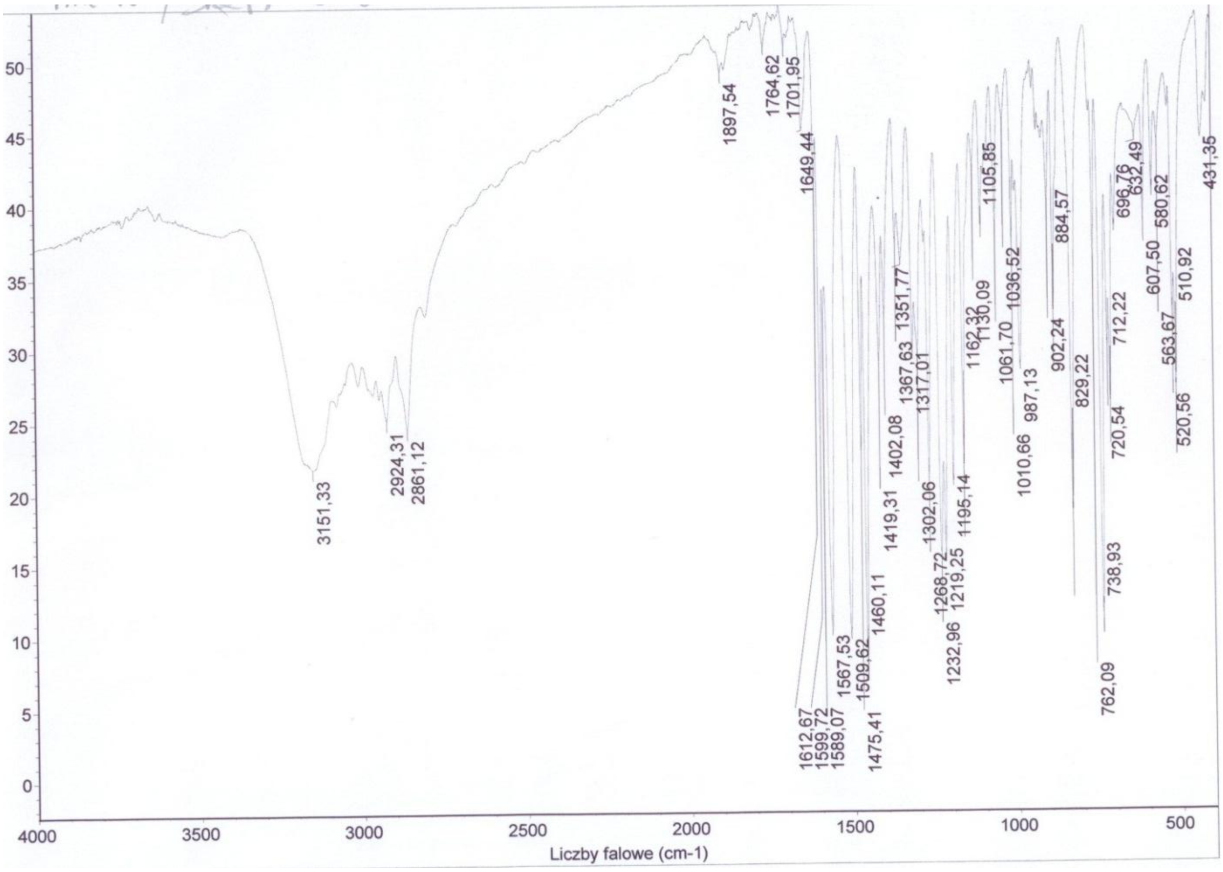

Figure S38. ^{1}H-NMR (100 MHz, DMSO-d_6) of (*Z*)-1-[1-(4,5-dihydro-1*H*-imidazol-2-yl)-2-(naphthalen-2-yl)vinyl]-1*H*-indole (**2i**).

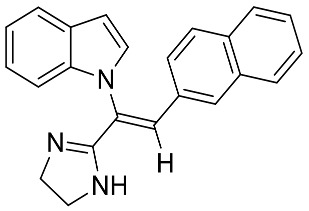

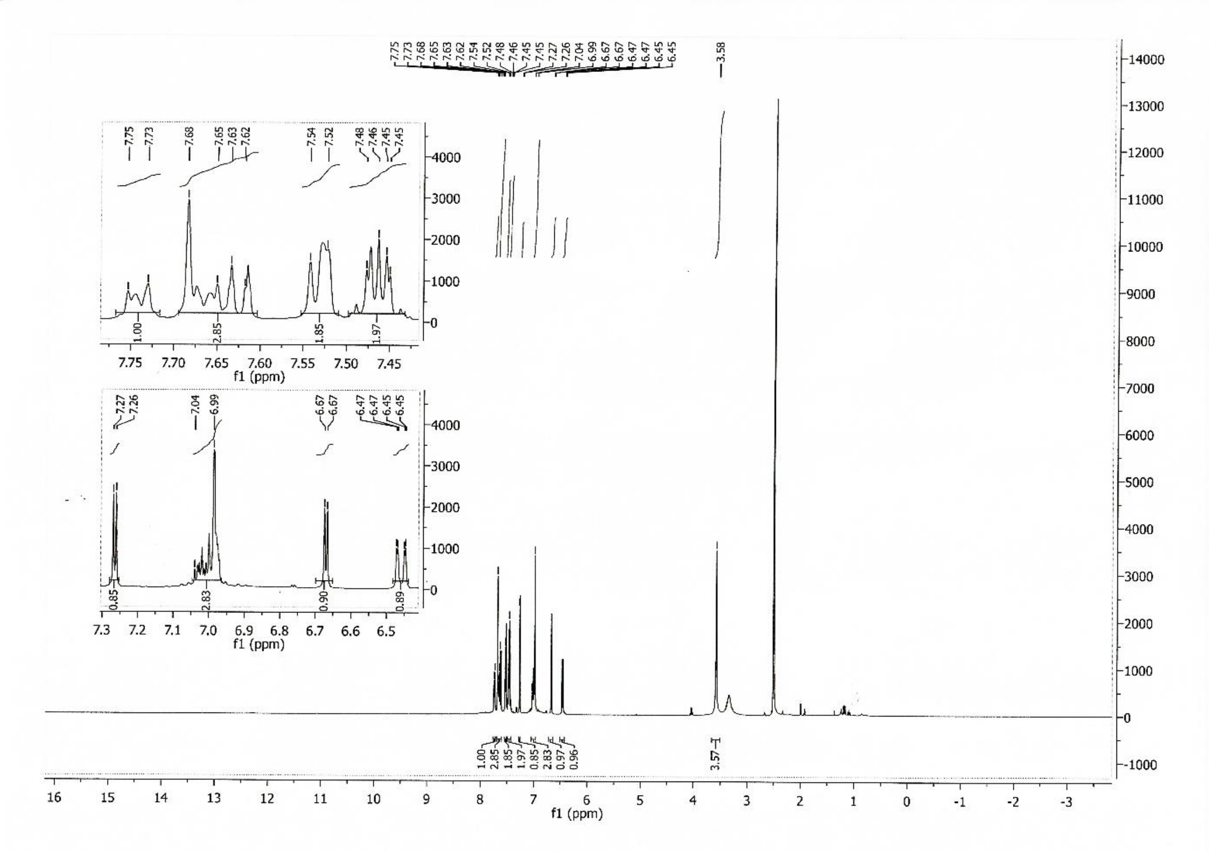

Figure S39. ¹³C-NMR (100 MHz, DMSO-d_6) of (*Z*)-1-[1-(4,5-dihydro-1*H*-imidazol-2-yl)-2-(naphthalen-2-yl)vinyl]-1*H*-indole (**2i**).

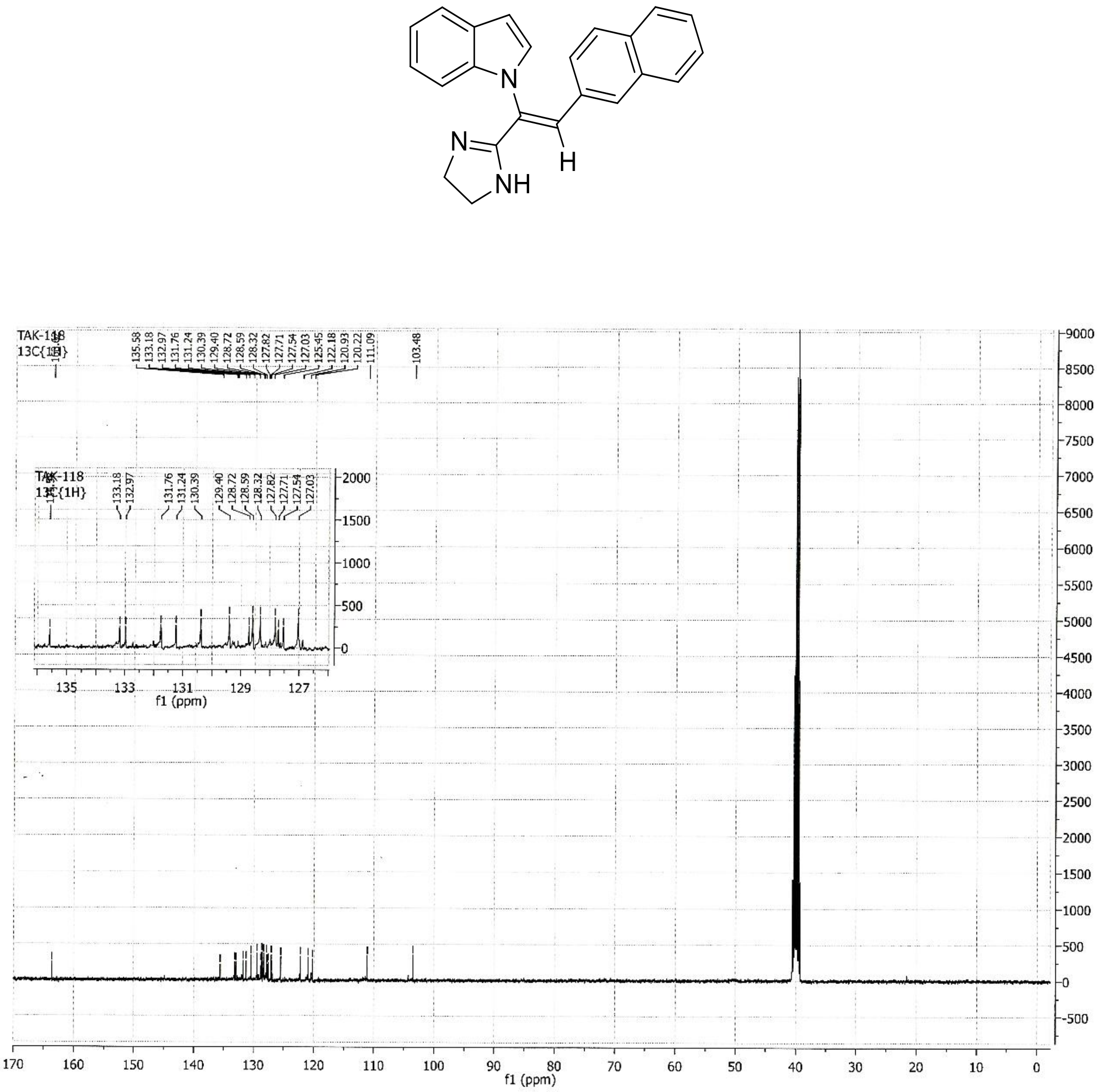

Figure S40. MS of (*Z*)-1-[1-(4,5-dihydro-1*H*-imidazol-2-yl)-2-(naphthalen-2-yl)vinyl]-1*H*-indole (**2i**).

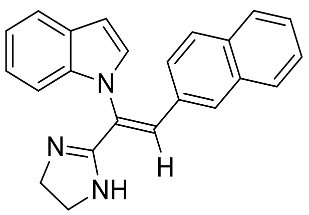

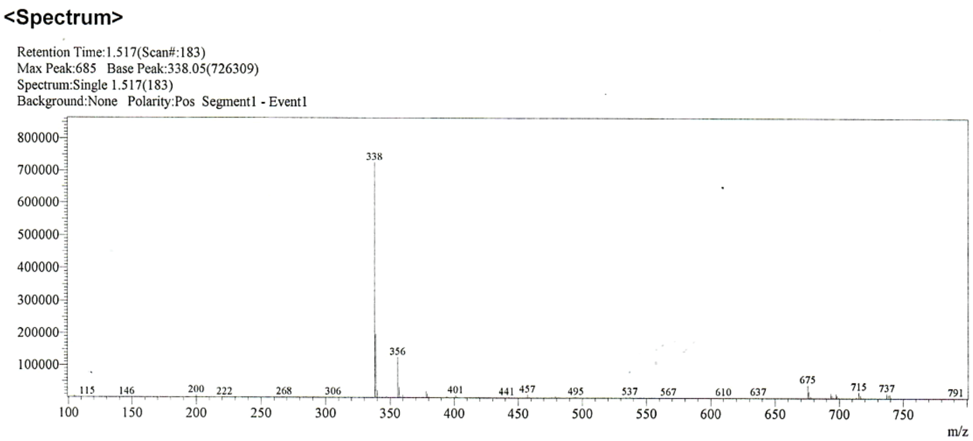

Figure S41. CheckCIF/PLATON report for (*Z*)-1-[1-(4,5-dihydro-1*H*-imidazol-2-yl)-2-(4-nitrophenyl)vinyl]-1*H*-indole (**2g**).

```
Bond precision:    C-C = 0.0026 A           Wavelength=0.71073

Cell:              a=16.4478(7)     b=10.7316(4)       c=9.8905(4)
                   alpha=90         beta=105.885(4)    gamma=90
Temperature:       295 K

                  Calculated                  Reported
Volume            1679.12(12)                 1679.12(12)
Space group       P 21/c                      P 21/c
Hall group        -P 2ybc                     -P 2ybc
Moiety formula    C19 H16 N4 O2               C19 H16 N4 O2
Sum formula       C19 H16 N4 O2               C19 H16 N4 O2
Mr                332.36                      332.36
Dx,g cm-3         1.315                       1.315
Z                 4                           4
Mu (mm-1)         0.089                       0.089
F000              696.0                       696.0
F000'             696.28
h,k,lmax          20,13,12                    20,13,12
Nref              3180                        3175
Tmin,Tmax         0.958,0.965                 0.980,1.000
Tmin'             0.956

Correction method= # Reported T Limits: Tmin=0.980 Tmax=1.000
AbsCorr = MULTI-SCAN

Data completeness= 0.998           Theta(max)= 25.682

                                                  wR2(reflections)=
R(reflections)= 0.0458( 2374)                     0.1105( 3175)

S = 1.025                   Npar= 271
```

```
Alert level C
PLAT220_ALERT_2_C NonSolvent    Resd 1  C   Ueq(max)/Ueq(min) Range        3.1 Ratio
PLAT242_ALERT_2_C Low    'MainMol' Ueq as Compared to Neighbors of         N23 Check
PLAT906_ALERT_3_C Large K Value in the Analysis of Variance ......       6.439 Check
PLAT910_ALERT_3_C Missing FCF Reflection(s) Below Theta(Min)[Deg]=        3.20 Note
                1  0  0,   1  1  0,   2  0  0,  -1  1  1,   0  1  1,
```

```
Alert level G
PLAT002_ALERT_2_G Number of Distance or Angle Restraints on AtSite          22 Note
PLAT172_ALERT_4_G The CIF-Embedded .res File Contains DFIX Records           2 Report
PLAT175_ALERT_4_G The CIF-Embedded .res File Contains SAME Records           1 Report
PLAT176_ALERT_4_G The CIF-Embedded .res File Contains SADI Records           4 Report
PLAT186_ALERT_4_G The CIF-Embedded .res File Contains ISOR Records           1 Report
PLAT187_ALERT_4_G The CIF-Embedded .res File Contains RIGU Records           1 Report
PLAT301_ALERT_3_G Main Residue  Disorder .............(Resd    1)          40% Note
PLAT411_ALERT_2_G Short Inter H...H Contact  H21      ..H7A      .        2.11 Ang.
                                                  x,y,1+z  =      1_556 Check
PLAT811_ALERT_5_G No ADDSYM Analysis: Too Many Excluded Atoms ....           ! Info
PLAT860_ALERT_3_G Number of Least-Squares Restraints ............          227 Note
PLAT883_ALERT_1_G Absent Datum for _atom_sites_solution_primary ..     Please Do !
PLAT969_ALERT_5_G The 'Henn et al.' R-Factor-gap value ..........        5.967 Note
              Predicted wR2: Based on SigI**2  1.85 or SHELX Weight 10.78
PLAT978_ALERT_2_G Number C-C Bonds with Positive Residual Density.           1 Info
```

```
 0 ALERT level A = Most likely a serious problem - resolve or explain
 0 ALERT level B = A potentially serious problem, consider carefully
 4 ALERT level C = Check. Ensure it is not caused by an omission or oversight
13 ALERT level G = General information/check it is not something unexpected

 1 ALERT type 1 CIF construction/syntax error, inconsistent or missing data
 5 ALERT type 2 Indicator that the structure model may be wrong or deficient
 4 ALERT type 3 Indicator that the structure quality may be low
 5 ALERT type 4 Improvement, methodology, query or suggestion
 2 ALERT type 5 Informative message, check
```
